# Supplementary material for: Protocol for a seamless phase 2A-phase 2B randomized double-blind placebo-controlled trial to evaluate the safety and efficacy of benfotiamine in patients with early Alzheimer’s disease (BenfoTeam)
Source: PLoS One. 2024 May 29;19(5):e0302998. doi: 10.1371/journal.pone.0302998 (PMC11135745; doi:10.1371/journal.pone.0302998)
Supplement: S1 File — (DOCX) [file pone.0302998.s002.docx]

**Statistical Methods: Transition from Phase 2A to Phase 2B**

To inform the bounds of an acceptable adverse event rate, we compared data from the high dose arm to the placebo to a recently completed double-blind randomized trial, which tested 18 months’ treatment with 300 participants with mild to moderate AD [1]. Over the first 8 months of the trial, with 20 participants and about 100 person-months of exposure in each arm, the tolerability event (TE) rate in the high dose arm was found to be about 0.21 events per subject per month, a factor of 3.2 times higher than the TE rate in the placebo (0.067 events per subject per month). Most of these events were moderate adverse events and none were serious, with a few dropouts, providing an example of a well-tolerated therapeutic agent. Thus, we will take the highest acceptable rate of TEs in the high dose benfotiamine arm to be 3.2 times higher than the placebo arm rate, reasoning that this should provide an ample safety and tolerability margin.

Table 1 shows the number of events needed for the conditional binomial test to have 80% power to detect a given rate ratio, for two assumed event rates in the placebo arm. Note that the number of events needed depends on the rate ratio, but not the baseline rate of TEs in the placebo arm. To detect an elevated rate ratio of 3.2 times or higher we will need to see about 21 total events before we conduct the test. Note that the number of person months needed to accumulate the desired number of events is less at higher event rates. Thus, we will lock the data and conduct the primary analysis when either 21 events have occurred, or when 160 person-months of exposure have occurred in each of the high dose and placebo arms, whichever is sooner. This assures that, in case the event rate is higher than expected, the analysis will be conducted as soon as it is adequately powered. However, if event rates are lower than expected, so that by 160 person-months of exposure in each arm fewer than 21 total events have occurred, no safety concerns are expected. Thus, it will be appropriate to conduct the test even with fewer than 21 events, and to send a full safety and tolerability report forward for Data Safety and Monitoring Board review. From our accrual models, we expect to accumulate the needed exposure time by about calendar month 9 of the trial or earlier.

**Table 1.** **Number of events needed for adequate power, and expected timing of the analysis**


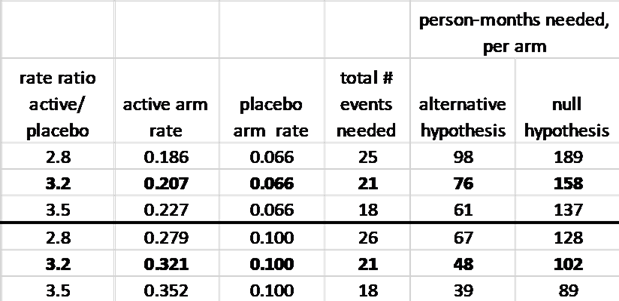


**References**

1. Schneider, L.S., et al., *Safety and Efficacy of Edonerpic Maleate for Patients With Mild to Moderate Alzheimer Disease: A Phase 2 Randomized Clinical Trial.* JAMA Neurol, 2019.
