# Supplementary material for: Protocol for a seamless phase 2A-phase 2B randomized double-blind placebo-controlled trial to evaluate the safety and efficacy of benfotiamine in patients with early Alzheimer’s disease (BenfoTeam)
Source: PLoS One. 2024 May 29;19(5):e0302998. doi: 10.1371/journal.pone.0302998 (PMC11135745; doi:10.1371/journal.pone.0302998)
Supplement: S2 File — (DOCX) [file pone.0302998.s003.docx]

**Data Availability Statement**

This protocol does not report experimental results and data collection is ongoing. De-identified data will be made available to qualified researchers upon study completion, following publication of the primary manuscript. URL: https://www.adcs.org/data-sharing/
