# Supplementary material for: Protocol for a seamless phase 2A-phase 2B randomized double-blind placebo-controlled trial to evaluate the safety and efficacy of benfotiamine in patients with early Alzheimer’s disease (BenfoTeam)
Source: PLoS One. 2024 May 29;19(5):e0302998. doi: 10.1371/journal.pone.0302998 (PMC11135745; doi:10.1371/journal.pone.0302998)

CLINICAL STUDY PROTOCOL

**Study Title:**	A Seamless Phase 2A-Phase 2B Randomized Double-Blind Placebo-Controlled Trial to Evaluate the Safety and Efficacy of Benfotiamine in Patients with Early Alzheimer's Disease (BenfoTeam)
**Funding Entity:**	National Institutes of Health (NIH)
**Protocol Number:**	ADC-061-BENFO
**US IND #:**	123557

Project Directors:

Gary E. Gibson, PhD
Burke Neurological Institute
Weill Cornell Medicine
785 Mamaroneck Ave
White Plains, NY 10605
ggibson@med.cornell.edu

Howard Feldman, MDCM, FRCP (C)
Alzheimer's Disease Cooperative Study
University of California, San Diego
9500 Gilman Drive, MC 0949
La Jolla, CA 92093-0949
hhfeldman@ucsd.edu

José A. Luchsinger, MD MPH
Columbia University Irving Medical Center
630 West 168th Street
New York, NY 10032
jal94@cumc.columbia.edu

Coordinating Center:

Howard Feldman, MDCM, FRCP (C)
Director and Principal Investigator
Alzheimer's Disease Cooperative Study
University of California, San Diego

Medical Monitor:

Gabriel C. Léger, MD
Director, Medical Safety Core
Alzheimer's Disease Cooperative Study
University of California, San Diego

SIGNATURE PAGE
(SIGNATURES ON FILE AT ADCS)

____ ____________________

Gary E. Gibson, PhD
Project Director
Burke Neurological Institute
Weill Cornell Medicine

Date (DDMMMYYYY)

____ ____________________

José A. Luchsinger, MD, MPH
Project Director
Columbia University Irving Medical Center

Date (DDMMMYYYY)

____ ____________________

Gabriel C. Léger, MD
Medical Monitor
Director, Medical Safety Core
Alzheimer's Disease Cooperative Study
University of California, San Diego

Date (DDMMMYYYY)

____ ____________________

Howard Feldman, MD
Director and Principal Investigator
Alzheimer's Disease Cooperative Study
University of California, San Diego

Date (DDMMMYYYY)

Study Summary

**Title**	A Seamless Phase 2A-Phase 2B Randomized Double-Blind Placebo-Controlled Trial to Evaluate the Safety and Efficacy of Benfotiamine in Patients with Early Alzheimer's Disease (BenfoTeam)
**Background and Rationale**	Effective treatments for Alzheimer's disease (AD) are urgently needed, as currently approved treatments have at best a small symptomatic benefit of limited duration, benefit only a subset of patients, and have no demonstrated effect on the underlying neuropathological processes.<br><br>Benfotiamine provides an important novel therapeutic direction in AD that has potential for additive or synergistic effects beyond current mainstream approaches. It has a unique mechanism of action, raising blood thiamine 50-100 times to pharmacological levels. In AD, it addresses and treats a well-characterized tissue thiamine deficiency and related changes in glucose metabolism as well as post-translational modifications that are linked to thiamine-dependent processes including neuroinflammation, abnormalities of advanced glycation end products (AGEs), plaques and tangles, and downstream neurodegeneration. Importantly, it further addresses the US National Alzheimer's Project Act (NAPA) therapeutic goal of combining targets.<br><br>Results from a small single-site pilot trial (with treatment arms of 35 participants) of 12 months in persons with early AD, demonstrated that benfotiamine was safe and well tolerated, with encouraging pharmacokinetic (PK) and pharmacodynamic (PD) responses. The trial also provided preliminary evidence of efficacy of benfotiamine on cognitive and functional outcomes. While the Alzheimer's Disease Assessment Scale-Cognitive Subscale (ADAS-Cog) did not reach nominal significance, it was trending in the correct direction and with a clear numerical benefit, with a 43% lesser mean decline in benfotiamine (-1.39) vs placebo (-3.26) p-values: mixed effect model ($p = 0.071$), GEE ($p = 0.137$), and a non-parametric Wilcoxon rank sum test ($p = 0.098$). Particularly important in the interpretation of this trial, is the converging evidence across outcome measures where the Clinical Dementia Rating (CDR) showed a strong significant benefit on this composite of cognition and everyday function ($p=0.034$) with other treatment effects in the correct direction across measures including a strong effect on patterns of glucose utilization ($p=0.002$). We take this as encouraging pilot efficacy data for a first phase 2 study.<br><br>The preliminary results of the pilot study provide proof of principle that justify testing the efficacy of benfotiamine in a larger seamless phase 2A-phase 2B randomized controlled trial (RCT) to investigate the safety, tolerability, and efficacy of benfotiamine in early AD. This trial will provide the next level of necessary evidence to establish "Proof of Concept" for

	benfotiamine as a widely available and clinically important treatment for early AD.
**Study Design & Number of Participants**	This is a randomized, double-blind, placebo-controlled 18-month clinical trial of benfotiamine in early AD. This trial will include a seamless phase 2A-2B design with a randomized total sample of 406 participants.<br><br>Participants who are randomized but drop out prior to study drug exposure will be replaced.
**Number of Clinical Sites**	Recruitment and enrollment of participants will occur through the Alzheimer's Disease Cooperative Study (ADCS) network in approximately 50 sites in the U.S.
**Target Population**	Participants with early AD who are screened to be amyloid positive, including participants with MCI due to AD and with Mild dementia due to AD (NIA-AA Diagnostic Criteria, 2011), will be included in the study population.<br><br>Key inclusion criteria include:<br>• Aged 50-89 (inclusive) at screening<br>• Mini-Mental State Examination (MMSE) total score 20-30, inclusive<br>• Montreal Cognitive Assessment (MoCA) < 26<br>• CDR global score of 0.5 or 1 (MCI=0.5, mild AD 0.5-1.0) with memory score ≥ 0.5 at screening<br>• Positive plasma AD biomarker signature:<br>▪ Plasma test (C_2N PrecivityAD2) that incorporates Aβ1-42 and Aβ1-40 and their ratio, age and p-tau217 into its predictive model. To qualify participants must have a High Amyloid Probability Score (APS2) .<br>▪ For participants who are clinically symptomatic and who have been treated with anti-amyloid therapy, and no longer meet the threshold for High APS according to the PrecivityAD2 test, a previous positive AD biomarker result (pre-treatment), including amyloid Positron-Emission Tomography (PET) scan with an FDA approved agent and a formal read, or AD fluid biomarker result of CSF or plasma consistent with the laboratory-based criteria for that particular assay, are eligible for inclusion.

**Dose Strategy & Treatment Duration**	Phase 2A of the trial will randomize approximately 150 participants total.<br>• 1:1:1 to treatment with 1200 mg/day benfotiamine, 600 mg/day benfotiamine or placebo.<br>At the end of phase 2A, the highest safe and well-tolerated dose of benfotiamine will be carried forward to phase 2B efficacy studies.<br>• The Phase 2A dose selection analysis will be conducted when 160 person-months of exposure have accumulated in each of the high dose and placebo arms, or alternatively when 21 total TEs have been observed across the high dose arm and the placebo arms combined, whichever is earlier.<br>The highest tolerated dose of benfotiamine will be carried forward from phase 2A to phase 2B. At the start of phase 2B, all participants enrolled in the two phase 2A active dose arms will receive a new supply of benfotiamine at the selected phase 2B dose. All phase 2A participants will be included in the phase 2 intent-to-treat efficacy population, as assigned to active or placebo treatment.<br>• Newly enrolled participants will be randomized 1:2 active arm to placebo arm until parity is achieved between numbers of active and placebo arm participants (approximately 150 participants total) and randomized 1:1 thereafter (approximately 100 participants).<br>• Total Treatment duration: 72 weeks
**Phase 2A Primary Objective**	The primary objective of phase 2A is to determine the highest safe and well-tolerated dose of benfotiamine (600 mg or 1200 mg), as evaluated by the rate of tolerability events (TEs), for advancement to long-term 72 week exposure.
**Phase 2A Secondary Objective**	The secondary objective of phase 2A is to evaluate other measures of safety and tolerability in multiple doses of benfotiamine (600 mg or 1200 mg).
**Phase 2B Primary Objective**	The primary objective of phase 2B is to assess efficacy of benfotiamine on global function and cognition over 72 weeks.
**Phase 2B Secondary Objectives**	The secondary objectives of phase 2B are:<br>• To evaluate longer-term safety and tolerability of benfotiamine treatment over 72 weeks.<br>• To evaluate the effects of benfotiamine on other measures of cognition, and its PK relationships to primary clinical outcome measures, imaging measures and PD biomarkers of AD, over 72 weeks.<br>• To evaluate preservation or potential improvement in everyday activities of daily living, as well as changes and potential benefits on other measures of cognitive function.

**Phase 2B Exploratory Objectives**	The exploratory objectives of phase 2B are:<br>• To evaluate the downstream biological effects of treatment with benfotiamine on measures of neurodegeneration (cortical thickness MRI, neurofilament light chain, total tau), neuroinflammation (glial fibrillary acid protein) and AD pathophysiology (p-tau 231, Aβ 42/40) over 72 weeks.<br>• To evaluate the effects of benfotiamine on neuropsychiatric symptoms and measures of cognition and function, including cognitive-functional composite measures, over 72 weeks<br>• To evaluate the effects of benfotiamine on measures of cognition administered remotely, and to compare the sensitivity of remote and in-person cognitive assessments over 72 weeks.
**Phase 2A Primary Endpoints**	The primary safety endpoint in phase 2A is the rate of tolerability events (TEs) compared between active and placebo arms, at each dose.<br><br>A TE is defined as any of the following:<br>• Participant early discontinuation of study drug related to intolerability<br>• A post-randomization moderate or severe adverse event (AE) that is possibly, probably or definitely related to study drug
**Phase 2A Secondary Endpoints**	Secondary Safety and Tolerability endpoints for phase 2A include:<br>• AEs summarized by grade, attribution, and body systems, by number of events and by participant<br>• Physical exam abnormalities<br>• Vital signs (weight, blood pressure, pulse rate, temperature, respiration rate)<br>• Standard 12-lead resting Electrocardiogram (ECG) measures<br>• Summaries of safety laboratory tests<br>• Participant withdrawal rates<br>• Drug discontinuation rates
**Phase 2B Primary Endpoints**	The primary cognitive endpoint in phase 2B is the within-participant change from baseline to 72 weeks compared between active arm and placebo on the ADAS-Cog-13.<br><br>The primary functional endpoint in phase 2B is the within-participant change from baseline to 72 weeks compared between active arm and placebo on the CDR-SB.

**Phase 2B Secondary Safety and Tolerability Endpoints**	Secondary Safety and tolerability endpoints for phase 2B include:<br>• AEs summarized by grade, attribution and body system, by number of events and by participant<br>• Physical exam abnormalities<br>• Vital signs (weight, blood pressure, pulse rate, temperature, respiration rate)<br>• Standard 12-lead resting ECG measures<br>• Summaries of safety laboratory tests<br>• Participant withdrawal rates<br>• Drug discontinuation rates
**Secondary PK Endpoints**	PK measures of thiamine and its esters thiamine diphosphate (ThDP) and thiamine monophosphate (ThMP), as well as the activation of transketolase by ThDP will be assessed as blood markers of efficacy of drug delivery measured by blood samples collected at baseline, week 8, and week 72.
**Phase 2B Secondary Efficacy Endpoints**	Additional efficacy endpoints of benfotiamine in phase 2B will be assessed by the within-participant change from baseline to week 72 between active and placebo on the following:<br>• The Alzheimer's Disease Cooperative Study – Activities of Daily Living Scale for use in Mild Cognitive Impairment (ADCS-ADL-MCI) to assesses the competence of patients with Alzheimer's Disease (AD) in basic and instrumental activities of daily living<br>• MoCA to evaluate changes and potential benefits in cognitive function
**Phase 2B Exploratory Endpoints**	The following exploratory endpoints in phase 2B are the within-participant longitudinal change between active and placebo in:<br>• Volumetric measures of hippocampal, whole brain, and ventricles as well as regional cortical thickness measured by cranial MRI<br>• Neuropsychiatric Inventory (NPI) to evaluate changes and potential benefits in neuropsychiatric symptoms<br>• Neuropsychological Test Battery (NTB; Remote subtests and In-Person subtests)<br>• ADAS-Cog-Exec<br>• Plasma biomarkers Aβ42/ Aβ40 ratio, total tau, p-tau 231, NfL and GFAP<br>• Increased AGE levels measured in plasma as an exploratory endpoint of PD.<br>• PK relationships to PD biomarkers and to clinical outcome measures.<br><br>Exploratory endpoints will be analyzed by disease severity groups, that is, by MCI due to AD and Mild dementia due to AD.

STUDY SCHEMATIC

Seamless Phase 2A-2B

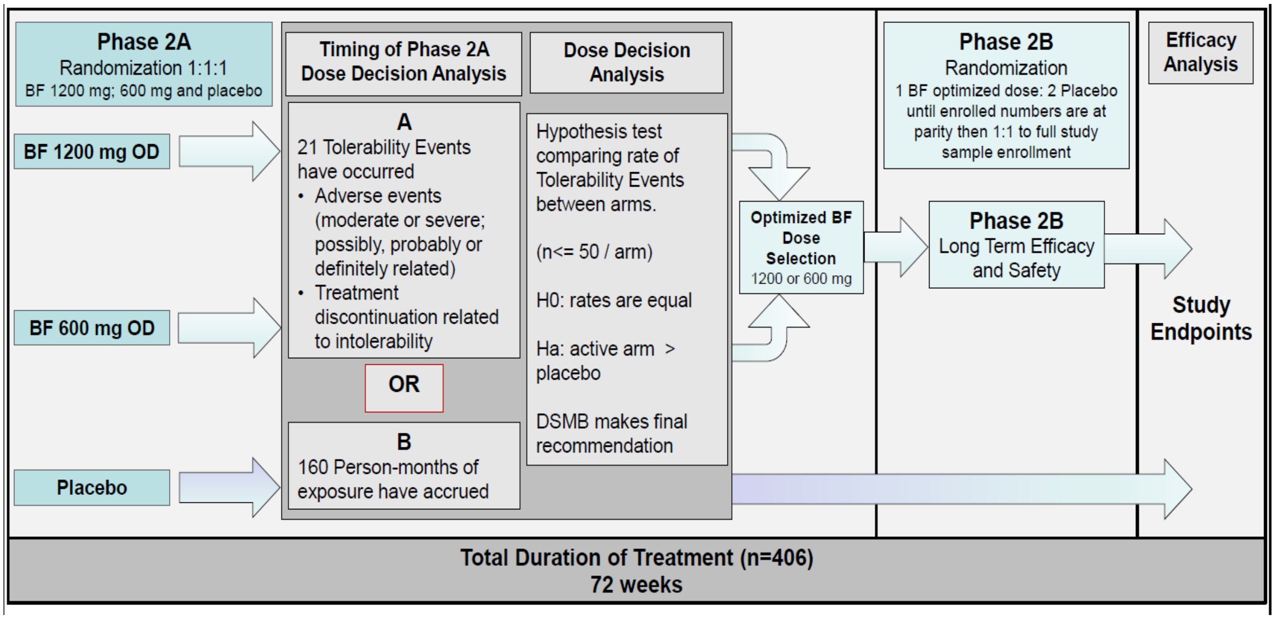

TABLE OF CONTENTS

1 INTRODUCTION 16
1.1 Background 16
1.2 Preclinical & Scientific Rationale 16
1.3 Clinical Data 20
1.3.1 Adverse Event Profile 20
1.3.2 Dose Selection 21
2 STUDY DESIGN 22
3 OBJECTIVES 23
3.1 Overall Goal 23
3.2 Phase 2A 23
3.2.1 Primary Objective 23
3.2.2 Secondary Objective 23
3.3 Phase 2B 23
3.3.1 Primary Objective 23
3.3.2 Secondary Objectives 23
3.3.3 Exploratory Objectives 23
4 ENDPOINTS 24
4.1 Phase 2A 24
4.1.1 Primary Endpoint 24
4.1.2 Secondary Endpoints 24
4.2 Phase 2B 24
4.2.1 Primary Endpoints 24
4.2.2 Secondary Endpoints 25
4.2.3 Exploratory Endpoints 25
5 ETHICS AND REGULATORY CONSIDERATIONS 26
5.1 Good Clinical Practice 26
5.2 Institutional Review Board 26
5.3 Informed Consent and HIPAA Compliance 27
5.4 Patient Confidentiality | HIPAA 28
5.5 Potential Risks and Benefits Associated with this Study 28
5.5.1 Potential Risks 28
5.5.2 Potential Benefits 28
6 INVESTIGATIONAL PRODUCT 28
6.1 Investigational Product 28
6.1.1 Investigational Product Intake 29
6.2 Coding, Packing and Labeling 29
6.3 Randomization and IP Ordering System 29
6.4 Phase 2A Dose Decision 30
6.5 Missed Doses 30
6.6 Blinding 31
6.7 Drug Accountability 31
7 PATIENT SELECTION AND CONCOMITANT MEDICATIONS 31
7.1 Inclusion Criteria 31

7.2 Exclusion Criteria 33
7.3 Concomitant Medications 34
7.3.1 Concomitant AD Medication 34
7.3.2 Other Prohibited Concomitant Medications 35
7.3.3 Prohibited Concomitant Medications for Optional Lumbar Puncture 35
8 STUDY PROCEDURES 36
8.1 Study Visits 36
8.1.1 Screening (within 42 days prior to baseline) 36
8.1.2 Baseline (Week 0) 37
8.1.3 Week 4 (+ 7 days) 38
8.1.4 Week 8 (+ 7 days) 39
8.1.5 Week 12 (+ 7 days) 39
8.1.6 Week 24 (+ 7 days) 40
8.1.7 Week 36 (+ 7 days) 40
8.1.8 Week 48 (+ 7 days) 41
8.1.9 Week 60 (+ 7 days) 41
8.1.10 Week 72 or Early Termination (+ 7 days) 42
8.1.11 Week 76 or Post-Treatment Safety Follow-up (+ 7 days) 43
8.1.12 Unscheduled Visits 43
8.2 Laboratory Safety Assessments 43
8.3 Plasma Assessments for PK, PD and Biomarkers 44
8.3.1 C_2N PrecivityAD2™ Test 45
8.4 Optional Lumbar Puncture and CSF Samples 45
8.5 Physical and Neurological Examination 46
8.5.1 Vital Signs 46
8.6 Electrocardiogram 46
8.7 Cranial MRI Assessments 47
9 STUDY-SPECIFIC INSTRUMENTS 48
9.1 Cognitive Measures 48
9.1.1 Neuropsychological Test Battery (NTB) 48
9.1.2 ADAS Cognitive Subscale-13 (ADAS-Cog-13) 49
9.1.3 Mini-Mental State Examination (MMSE) 50
9.1.4 Montreal Cognitive Assessment (MoCA) 50
9.1.5 ADAS-Cog-Exec 50
9.2 Behavioral and Functional Measures 50
9.2.1 Clinical Dementia Rating (CDR) Scale – Sum of Boxes (SOB) 50
9.2.2 The Alzheimer's Disease Cooperative Study – Activities of Daily Living Scale for use in Mild Cognitive Impairment (ADCS-ADL-MCI) 51
9.2.3 Neuropsychiatric Inventory (NPI) 51
9.2.4 Modified Hachinski Ischemic Scale (MHIS) 51
9.2.5 Columbia-Suicide Severity Rating Scale (C-SSRS) 51
9.2.6 CAGE-AID Substance Abuse Screening Tool 51
9.3 Research Satisfaction Survey 52
9.4 Treatment Blinding Questionnaire 52
10 EARLY DISCONTINUATION/WITHDRAWAL PROCEDURES 52
11 DEFINITION OF ADVERSE EVENTS 53

11.1 Evaluation and Reporting of Adverse Events 54
11.2 Assessment of Adverse Events 54
11.3 Collection and Reporting of Serious Adverse Events 55
11.3.1 Suspected Unexpected Serious Adverse Reactions 56
11.4 Overdose Reporting Requirements 56
11.5 Clinical Laboratory Abnormalities and Other Abnormal Assessments 57
11.6 Pregnancy and Breast Feeding 57
12 STATISTICAL METHODS 57
12.1 Analysis Populations and sensitivity analyses 57
12.2 Phase 2A 58
12.2.1 Overview 58
12.2.1 Phase 2A safety and tolerability analysis. 58
12.3 Transition from Phase 2A to Phase 2B 59
12.4 Phase 2B 60
12.4.1 Overview 60
12.4.2 Sample Size, Power and Effect Sizes 61
12.4.3 Analysis Plan 63
12.5 Safety Analysis 65
12.5.1 Adverse Events 65
12.5.2 Laboratory Parameters 65
12.5.3 Other Safety Parameters 65
12.6 Protocol Deviations, Data Blind Review, and Unblinding 66
13 RECORDING AND COLLECTION OF DATA 66
13.1 Case Report Form 66
13.2 Study Files and Patient Source Documents 67
13.3 Rater Training 67
13.4 Monitoring 67
13.5 Audit 68
13.6 Retention of Data 68
13.7 Reporting of Study Results 68
13.8 Quality Assurance/Quality Control 68
14 DATA SAFETY MONITORING BOARD 69
15 PUBLICATIONS POLICY AND SHARING OF DATA 69
16 APPENDICES 77
16.1 APPENDIX I: STUDY PLAN AND PROCEDURES AT EACH VISIT 77
16.2 APPENDIX II – NIA/AA DIAGNOSTIC GUIDELINES 80
16.3 APPENDIX III – CONSORT CHECKLIST & DIAGRAM (v2010) 83

List of Abbreviations:

Aβ	Beta-amyloid
AchEI	Acetylcholinesterase Inhibitors
AD	Alzheimer's Disease
ADAS- Cog13	Alzheimer's Disease Assessment Scale-Cognitive Subscale 13
ADCS-ADL-MCI	The Alzheimer's Disease Cooperative Study – Activities of Daily Living Scale for use in Mild Cognitive Impairment
ADNI	Alzheimer's Disease Neuroimaging Initiative
AE	Adverse Event
AGE	Advanced Glycation End products
AICDs	Automatic Implanted Cardioverter Defibrillators
AIDS	Acquired Immunodeficiency Syndrome
APOE	Apolipoprotein E
APP	Amyloid Precursor Protein
ATP	Adenosine Triphosphate
BACE1	Beta-secretase 1
BID	Twice a day
CDR	Clinical Dementia Rating
CDR-SB	Clinical Dementia Rating Scale-Sum of Boxes
CEL	Carboxyethyl-Lysine
CFR	Code of Federal Regulations
CIEDs	Cardiac Implantable Electronic Devices
CJD	Creutzfeldt-Jakob disease
CML	Carboxymethyl-Lysine
CNS	Central Nervous System
CONSORT	Consolidated Standards of Reporting Trials
C-SSRS	Columbia Suicide Severity Rating Scale
CSF	Cerebrospinal Fluid
DSMB	Data Safety Monitoring Board
DMP	Data Management Plan
EDC	Electronic Data Capture
ECG	Electrocardiogram
eCRF	Electronic Case Report Form
CRF	Case Report Form
FDA	Food and Drug Administration
FDGPET	Fluorodeoxyglucose Positron Emission Tomography
FDG	Fluorodeoxyglucose
GCP	Good Clinical Practice
GEE	Generalized Estimating Equation
GFAP	Glial Fibrillary Acidic Protein
GLO-1	Glyoxalase-1
GLP-1	Glucose Like Peptide 1
HAV	Hepatitis A Virus

HbA1c	Hemoglobin A1c
HBV	Hepatitis B Virus
HCV	Hepatitis C Virus
HDPE	High-Density Polyethylene
HIPAA	Health Insurance Portability and Accountability Act
HIV	Human Immunodeficiency Virus
Hb	Hemoglobin
HCT	Hematocrit
HED	Human Equivalent Dose
ICH	International Conference on Harmonization
ICMJE	International Committee of Medical Journal Editors
ITT	Intent-to-Treat
IRB	Institutional Review Board
IRT	Interactive Response Technology
KGDHC	α-Ketoglutarate Dehydrogenase Complex
LAR	Legally Authorized Representative
LBD	Lewy Body Dementia
LP	Lumbar Puncture
MCH	Mean Corpuscular Hemoglobin
MCHC	Mean Corpuscular Hemoglobin Concentration
MCI	Mild Cognitive Impairment
MCV	Mean Corpuscular Volume
MHIS	Modified Hachinski Ischemic Scale
mITT	Modified Intention-to-Treat
MMSE	Mini-Mental State Examination
MoCA	Montreal Cognitive Assessment Score
MRI	Magnetic Resonance Imaging
NAPA	National Alzheimer’s Project Act
NCRAD	National Cell Repository for Alzheimer’s Disease
NIA	National Institute on Aging, under the NIH
NIA-AA	National Institute on Aging and Alzheimer's Association
NIH	National Institutes of Health
NPH	Normal Pressure Hydrocephalus
NPI	Neuropsychiatric Inventory
OHRP	Office for Human Research Protections
PD	Pharmacodynamic
PDHC	Pyruvate Dehydrogenase Complex
PET	Positron Emission Tomography
PHI	Protected Health Information
PI	Principal Investigator
PK	Pharmacokinetic
PLT	Platelet Count
POC	Proof of Concept
PS	Phosphatidylserine

PSP	Progressive Supranuclear Palsy
PTM	Post- Translational protein Modification
QUARC	Quantitative Anatomic Regional Change
RAGE	Receptor of Advanced Glycation End products
RBC	Red Blood Cell ount
RCT	Randomized Controlled Trial
RPR	Rapid Plasma Reagin
SAE	Serious Adverse Event
SAP	Statistical Analytical Plan
SD	Standard Deviation
SOP	Standard Operating Procedures
t-tau	total tau
TCA	Tricarboxylic Acid
TE	Tolerability Events
ThDP	Thiamine Diphosphate
ThMP	Thiamine Monophosphate
TK	Transketolase
TPP	Thiamine Pyrophosphate
TSH	Thyroid Stimulating Hormone
WBC	White Blood Cell Count
WK	Wernicke-Korsakoff

1 INTRODUCTION

1.1 Background

Alzheimer's disease (AD) is a progressive neurologic disorder that is the most common cause of dementia with devastating socioeconomic implications. More than 5 million people are afflicted with AD and related dementias in the United States, and by 2050 this number could rise as high as 16 million, with annual costs of over $1 trillion [1]. The emotional and financial burden of AD to patients, family members, and society is enormous, and is predicted to grow exponentially as the median population age increases. The potential to preserve, or even improve, cognition in adults at high risk of cognitive decline due to AD clearly has important implications, not only for the affected individual, but also for the support system that bears the social and financial burdens of long-term caregiving.

Benfotiamine provides an important novel therapeutic direction in AD that has potential for additive or synergistic effects beyond current mainstream approaches. It has a unique mechanism of action, raising blood thiamine 50-100 times to pharmacological levels. In AD, it addresses and treats a well-characterized tissue thiamine deficiency and related changes in glucose metabolism as well as post-translational modifications including plaque [2] and tangle formation [3], neuroinflammation [4, 5], neurodegeneration [6], and advanced glycation end products (AGEs) [7]. Importantly, it further addresses the US National Alzheimer's Project Act (NAPA) therapeutic goal of combining targets [8].

1.2 Preclinical & Scientific Rationale

Thiamine (vitamin B1) dependent processes are critical in glucose metabolism. Normal brain function depends upon a continuous supply of glucose, which requires three thiamine dependent enzymes: transketolase (TK), the pyruvate dehydrogenase complex (PDHC) and the α-ketoglutarate dehydrogenase complex (KGDHC). TK is a critical enzyme in the pentose shunt. PDHC links glycolysis to the tricarboxylic acid (TCA) cycle. KGDHC is arguably the rate-limiting step in the tricarboxylic acid (TCA) cycle. Thus, thiamine regulates essential aspects of brain glucose metabolism. Thiamine is phosphorylated to thiamine diphosphate ester (frequently referred to as thiamine pyrophosphate, TPP), which is the form of thiamine that is required by these enzymes.

Thiamine → Thiamine Monophosphate (ThMP) → Thiamine Diphosphate (ThDP or TPP).

The critical value of thiamine for normal brain function has led to careful characterization of the blood-brain transport of thiamine [9, 10]. Brain has both a saturable transport system and a non-saturable import system – perhaps diffusion. Thus, benfotiamine, which increases plasma levels longer and higher than thiamine itself, also elevate brain thiamine more than thiamine itself.

Thiamine-dependent processes are diminished in the periphery and brains of patients with AD. Measurement in peripheral tissues and brain suggests that thiamine-dependent processes may be altered specifically in AD [11]. Plasma thiamine levels are diminished in AD patients and not in Parkinson patients [12]. The TPP effect on transketolase, a classic measure of functional thiamine deficiency, also reveals functional thiamine deficiency in AD patients

compared with controls [11].The only way to assess changes in thiamine- dependent enzymes in human brain is at autopsy, and those results suggest that diminished activities of thiamine-dependent enzymes underlie the glucose deficits. Dr. Gibson's group and others have demonstrated an AD-related reduction in all thiamine-dependent processes including transketolase, PDHC and KGDHC [11, 13-15]. We propose that these reductions underlie the decline in glucose metabolism. The reduction in these enzymes is highly correlated with the clinical dementia rating (CDR) score in AD patients prior to death (r= 0.77) [16]. Furthermore, evidence suggests that this is a functional thiamine deficiency. Of the enzymes examined, the protein levels do not decline [11, 13-15] and TPP increases thiamine-dependent enzymes more in brain samples from AD brain than from controls [17].

Increasing thiamine levels may promote proper glucose utilization by the brain in AD. The essential role of thiamine-dependent processes in critical steps of metabolism, the decline of thiamine dependent processes in AD brain tissue, and the decline of on Fluorodeoxyglucose (FDG) Positron Emission Tomography (PET) in AD, suggest that increasing thiamine might improve function by promoting the proper utilization of glucose. Merely increasing glucose entry into the brain as measured by FDG PET, may not be adequate, because thiamine deficiency alters the pathways of glucose utilization (i.e., pentose shunt, glycolysis and tricarboxylic acid cycle). Thus, administering thiamine differs from other approaches, including approaches that use drugs to promote glucose availability by manipulation of peripheral glucose such as studies of Liraglutide, a glucose like peptide 1 (GLP-1) agonist or other insulin-like drugs [18, 19]. Raising thiamine is a novel approach, which promotes the possibility for the brain to use glucose properly.

Benfotiamine increases blood/brain thiamine to much higher levels than thiamine. Pharmacokinetic and pharmacodynamic (PK/PD) studies show that benfotiamine, a thiamine prodrug, is much better at increasing thiamine bioavailability than thiamine [20]. A comparison of the area under the concentration time curve for blood following benfotiamine (4948 μg thiamine over time) or thiamine (101 μg thiamine over time), shows that benfotiamine is 49 times more effective at raising blood thiamine levels. In Dr. Gibson's pilot clinical trial, one-year treatment with benfotiamine increased thiamine 161 times over baseline [7]. Furthermore, in an animal model of tangles, benfotiamine increased brain thiamine levels 25 times more than thiamine [21]. PK studies in healthy humans demonstrate a strong dose-response increase in thiamine and its esters [22].

Brain thiamine deficiency can exist without nutritional thiamine deficits. Thus, we propose to increase thiamine levels to pharmacologically high levels independent of nutritional status. Assessing thiamine status is difficult because requirements differ from person to person [23]. Our well-developed approach is to measure the values of thiamine, TPP and ThMP, as well as the TPP effect on transketolase activity [24-26]. Multiple conditions can alter thiamine transport to the brain or its utilization or mobilization in or between cellular compartments without altering blood thiamine status. Conditions leading to tissue-level thiamine deficiency include: reduced thiamine intake (e.g., gluten free diet, dialysis, celiac disease, bariatric surgery, excessive vomiting); increased thiamine excretion (e.g., diabetes); altered ability to use thiamine (including references to at least 30 drugs); genetics (Organic cation transporter modulates multiple cardiometabolic traits through effects on thiamine content); and virus (feline leukemia virus

inhibits thiamine transporter) [27]. High peripheral thiamine can overcome these abnormalities by increasing tissue thiamine availability an approach we are proposing with benfotiamine.

Thiamine dependent post-translational modifications link metabolism to AD pathology; in particular, KGDHC-regulated succinylation links brain glucose metabolism to AD pathophysiology. The link between thiamine-dependent processes and the associated pathology has remained elusive, but it is well documented to not simply be adenosine triphosphate (ATP). Using cells, isolated proteins, mice, and autopsied brains, we discovered that succinylation, a newly discovered post- translational protein modification (PTM), links metabolism to plaque and tangle formation [6]. In autopsied brains, succinylation of amyloid precursor protein (APP) and tau co-occur at brain regions critical to plaque and tangle formation. Transgenic mouse studies demonstrate that succinylated APP (Tg19959 mice) and succinylated tau (P301S) are detected in the hippocampus concurrent with Beta-amyloid (Aβ) oligomers and insoluble fiber assemblies. Succinylation is therefore a first single PTM across multiple substrates, promoting amyloidosis, tauopathy, and glucose hypometabolism [6]. Since succinylation is controlled by KGDHC13 correcting thiamine dependent processes, which regulate succinylation provides an attractive and novel therapeutic target in AD.

Additionally, thiamine-dependent PTM's link thiamine deficiency to the pathophysiology of AD. Transketolase regulates the production of AGEs which are elevated in AD [28, 29]. We also demonstrated that PDHC can regulate acetylation, which has been linked to phosphorylation of tau and tangle formation [30].

We will measure changes in biomarkers of amyloid, tau, and neurodegeneration, in line with the Diagnostic Guidelines of the National Institute on Aging and Alzheimer's Association (NIA-AA) research framework [31], based on existing data linking thiamine deficiency to these AD constructs.

APP and plaque formation are sensitive to thiamine levels and benfotiamine decreases plaques in APP/presenilin-1 mice. Thiamine deficiency exacerbated amyloid plaque pathology making Tg19959 transgenic mice (over expressing a double mutant form of the APP) and enlarged the area occupied by plaques in the cortex, hippocampus, and thalamus by 50%, 200% and 200%, respectively. Thiamine deficiency increased Aβ1-42 levels by about three-fold and [beta]-secretase (BACE1) protein levels by 43% [32]. Reductions of the thiamine dependent KGDHC increases Aβ42 [33] and promotes plaque formation in mice [34]. KGDHC dependent succinylation blocks alpha secretase to promote plaque formation and to promote Aβ aggregation [6]. Free radical production due to reduced KGDHC activates BACE [34]. The most compelling evidence of thiamine's role in plaque formation is that benfotiamine causes a dose-dependent reduction in plaque formation and improvement in memory [2]. Thus, we propose to use plasma markers of Aβ42/ Aβ40 to monitor the effectiveness of benfotiamine in the proposed trial.

Total tau and tau phosphorylation are sensitive to thiamine levels and benfotiamine decreases tangles in P301S mice. Thiamine deficient humans (Wernicke Korsakoff patients) have tangles [35, 36]. In mice, thiamine deficiency increases phosphorylation of tau [33]. Our recent studies demonstrate that abnormalities in mitochondria lead to succinylation of tau by the thiamine dependent enzyme KGDHC. The succinylation blocks binding to microtubules and

promotes aggregation of tau [6]. Treatment with the thiamine prodrug benfotiamine diminishes phosphorylation of tau. This has been demonstrated in at least three different animal models of AD. Benfotiamine diminished the phosphorylation of tau in a mouse model bearing mutant human APP [2]. In an animal model of tangle formation (P301S), benfotiamine dose-dependently diminished tangles and improved behavioral outcomes [2, 3]. In P301S mice, we determined thiamine status i.e., transketolase activity of the brain, which demonstrated that the brains of the transgenic mice were significantly, thiamine deficient [3]. This was rather surprising since tangles in these models are associated with mutant genes. Benfotiamine increases thiamine pyrophosphate in the hippocampus in streptozotocin models of AD in rats, and diminishes phosphorylation of tau [37]. Consequently, in the current proposal, phosphorylation of p-tau 231 will be used as a plasma biomarker.

AGES are increased in AD, are sensitive to thiamine deficiency, and are decreased by benfotiamine. Elevated AGEs and their receptor, RAGE, occur in the brain [38] and periphery of AD patients [39-44] and are found in both plaques and tangles [45]. High concentrations of AGE are predictive of long-term decline in cognition-related daily living performance in patients with AD as measured by CDR or Mini-Mental State Examination (MMSE) [39]. Some AGEs such as pentosidine, a cross-linking AGE, seem directly linked to cognitive symptoms [46, 47]. AGEs have also been linked to Apolipoprotein E (APOE) genotype, with APOE4 carriers holding greater AGE, and the APOE4 molecule binding with greater affinity to AGE [48-52]. Thiamine deficiency increases AGEs, whereas elevating thiamine diminishes AGEs [53, 54]. Even marginal thiamine deficiency increases AGEs [54]. Benfotiamine/thiamine diminishes AGE by activating the thiamine-dependent enzyme transketolase. This effect first reported by Hammes, et al [28] has been very influential, with over a thousand citations. The activation of transketolase accelerates the precursors of AGEs towards the pentose phosphate pathway thereby reducing the production of carboxymethyllysine and pentosidine [29]. In addition to activating transketolase, thiamine increases transcription of transketolase [55]. A second well-established pathway for thiamine to diminish AGE is through the increased expression of the enzymes involved in the glyoxalase system, particularly glyoxalase 1 (GLO-1), which breaks down AGE precursors, primarily methylglyoxal [5] AGEs, MG-H1 and carboxyethllysine (CML), are produced from methylglyoxal. This means that benfotiamine, by reducing methylglyoxal, reduces the AGEs that are produced by this pathway. The most compelling evidence that these interactions are important, is that benfotiamine diminished AGE in Dr. Gibson’s pilot trial [7].

Thiamine also affects inflammation and glial activity and we will measure biomarkers of this important process in AD. Thiamine deficiency promotes inflammation whereas thiamine/benfotiamine diminish inflammation. Thiamine deficiency increases Glial fibrillary acidic protein (GFAP) and inflammation in parallel with neuronal loss [56]. Astrocytes as measured by GFAP are a major target of thiamine deficiency [33, 57] and in APP mutant mice, increases in GFAP parallel increases in plaques [33] and cause dramatic increases in brain p53. Pro-inflammatory cytokines inhibit thiamine uptake [4]. Thiamine/benfotiamine diminishes GFAP. In the streptozotocin model of AD, benfotiamine reverses the inflammation, diminishes GFAP, and is protective [37]. Thiamine is “an anti-inflammatory factor” and has an important effect on the activity of the p53 suppressor protein [58]. Thiamine connection to inflammation is demonstrated by 12,500 references in google scholar. Thus, we will measure plasma GFAP as a biomarker.

Thiamine also affects neurofilaments. We will measure biomarkers of this important process in AD. In non-transgenic mice, thiamine deficiency causes accumulation of neuritic clusters containing neurofilaments [32]. In APP/PS1 transgenic mice, the thiamine deficiency induced abnormalities in neurites co-localized with APP-like protein and neurofilament [59]. Thiamine deficiency induces a loss of axonal brain neurofilaments [60, 61]. Over 8,500 papers in Google Scholar address thiamine deficiency and central nervous system (CNS) degeneration. Benfotiamine/thiamine reverses the abnormal neurofilaments and neurites in tangles [3] and plaques [2].

1.3 Clinical Data

Results from a small single-site pilot trial (with treatment arms of 35 participants) of 12 months in persons with early AD, demonstrated that benfotiamine was safe and well tolerated, with encouraging PK and PD responses [7]. The treatment delivery was efficacious as shown by a 161-fold mean increase in blood thiamine. The trial also provided preliminary evidence of efficacy of benfotiamine on cognitive and functional outcomes. While the Alzheimer's Disease Assessment Scale-Cognitive Subscale (ADAS-Cog) did not reach nominal significance, it was trending in the correct direction and with a clear numerical benefit, with a 43% lesser mean decline in benfotiamine (-1.39) vs placebo (-3.26) p-values: mixed effect model ($p = 0.071$), GEE ($p = 0.137$), and a non-parametric Wilcoxon rank sum test ($p = 0.098$). Particularly important in the interpretation of this trial, is the converging evidence across outcome measures where the CDR showed a strong significant benefit on this composite of cognition and everyday function ($p=0.034$) with other treatment effects in the correct direction across measures including a strong effect on patterns of glucose utilization ($p=0.002$). Exploratory analyses revealed that the benfotiamine group showed better brain glucose uptake FDG PET patterning scores ($p=0.0329$). Plasma AGEs were reduced ($p=0.044$) [7].

The preliminary results of the pilot study provide proof of principle that justify testing the efficacy of benfotiamine in a larger seamless phase 2A-phase 2B randomized controlled trial (RCT) to investigate the safety, tolerability, and efficacy of benfotiamine in early AD. This trial will provide the next level of necessary evidence to establish “Proof of Concept” for benfotiamine as a widely available and clinically important treatment for early AD.

1.3.1 Adverse Event Profile

The safety of benfotiamine in humans has already been tested. Benfotiamine has been tested in multiple clinical trials because of its beneficial effect in the diabetic peripheral neuropathy. One study of 40 patients with 400 mg per day for three weeks reported no side effects [62]. In a second study, Stracke et al., [63] administered benfotiamine to 165 patients in ten different centers in Germany for six weeks. In both the 300 mg/day and 600 mg/day benfotiamine groups, the number of patients with adverse events was comparable to placebo demonstrating the good tolerability of a daily dose of 300 mg or 600 mg benfotiamine. Information related to treatments for Wernicke-Korsakoff syndrome (WK) indicates that mice are able to tolerate 22 times more benfotiamine by body weight than common vitamin B-1. After decades of use as a dietary supplement by many people, no adverse effects have been reported [64].

In Dr. Gibson's pilot trial, no adverse events related to the 600 mg (2×300 mg) benfotiamine per day were observed and patients did not complain about the medication in the single-site 12 month pilot trial of persons with early AD [7] (described above). Studies suggest that higher dosage is also likely safe in humans. Adult humans given 1200 mg of benfotiamine show no adverse events [22]. Multiple trials in diabetic participants also suggest higher dosages will be safe. Dosages of 300 to 900 mg per day caused no adverse events [63]. In a second trial, 31 people with Type 2 diabetes received 900 mg/day of benfotiamine or a placebo for 6 weeks. The therapy was well tolerated, and no drug-related serious adverse events occurred [65]. In several other studies in diabetics, 900 mg/day has been studied for up to 12 weeks with good safety and tolerability [66, 67].

1.3.2 Dose Selection

While the results of the pilot study of 600 mg of pharma- benfotiamine are encouraging, both preclinical studies and pharmacokinetic studies in healthy individuals suggest that a higher dose will have greater effect. In APP/PS1 transgenic mice, there is clear evidence of dose response relationships on both behavioral (memory on water maze) and pathological measures (reduced plaques) with human equivalent doses (HED) of 1200 mg having better results than 600 mg [2]. Powerful beneficial effects of benfotiamine on cognitive impairment and amyloid deposition were found in APP/presenilin-1 transgenic mice [2]. Pan et al. [2], showed that the 200 mg/kg reduced plaques more than 100 mg/kg. Similarly, 200 mg/kg improved memory better than 100 mg/kg in a water maze using crossing index and quadrant occupancy as indices.

Mouse dosages in mg/kg were converted to HED. The following rationale was used with body surface area to convert. As shown in Figure 1, the animal dose was multiplied to the scaling factor for mice dose (0.08) and multiplied by 70 for a 70 kg person. Thus, the mouse dosages convert to 300 mg, 600 mg, and 1200 mg, respectively. The mouse studies suggest doubling the dosage from 600 mg per day to 1200 mg per day will provide additional protection.

Figure 1.

Calculation of human equivalent dosage				
mg/kg mouse			mg per 70 kg human	mg per 70 kg rounded
50	70	0.08	280	300
100	70	0.08	560	600
200	70	0.08	1120	1200

Human dose range data in healthy human participants show that 1200 mg has superior PK and PD properties in raising blood thiamine, thiamine pyrophosphate, and thiamine monophosphate and that this is likely going to be safe and well-tolerated [22]. The results in Figure 2 show that 1200 mg of benfotiamine administered to healthy humans increases blood thiamine, ThDP and ThMP more than 600 mg. However, a dose of 1200 mg has not yet been tested in AD, and satisfactory safety and tolerability need to be established to avoid any unexpected challenges within the trial.

Figure 2.

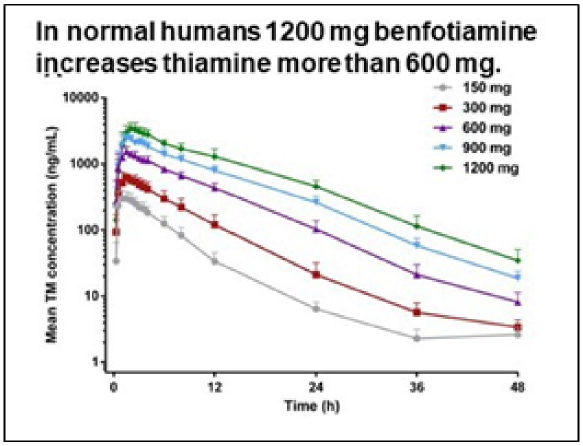

Through an innovative adaptive dose decision rule, we will efficiently evaluate safety and tolerability very early in the trial, thus optimizing the exposure to the highest and best-tolerated dose.

2 STUDY DESIGN

This is a randomized, seamless phase 2A-2B, double-blind, placebo-controlled 18-month clinical trial of benfotiamine in early AD with a maximum total sample size of 406 participants.

Phase 2A will determine the highest safe and well-tolerated dose of benfotiamine to be carried forward to phase 2B. Phase 2A will randomize approximately 150 participants 1:1:1 to treatment with 1200 mg/day benfotiamine, 600 mg/day benfotiamine or placebo. The randomization stratification factor will be site.

The Phase 2A safety and dose selection analysis will be conducted when 160 person-months of exposure have accumulated in each of the high dose and placebo arms, or alternatively when 21 total TEs have been observed across both the high dose arm and the placebo arm, whichever is earlier.

Phase 2B will assess efficacy and longer-term safety of benfotiamine in a larger group of participants through 72 weeks of treatment, at the selected dose. Throughout the remainder of the trial, all participants will be randomized to the phase 2A selected dose or placebo. At the start of phase 2B, all participants enrolled in the two phase 2A active dose arms will receive a new supply of benfotiamine at the selected phase 2B dose. All phase 2A participants will be included in the phase 2 intent-to-treat efficacy population, as assigned to active or placebo treatment.

3 OBJECTIVES

3.1 Overall Goal

The overall goal of this study is to conduct a seamless phase 2A-2B proof of concept (POC) trial investigating tolerability, safety, and efficacy of benfotiamine, a prodrug of thiamine as a first-in-class small molecule treatment for early Alzheimer's disease (AD).

3.2 Phase 2A

3.2.1 Primary Objective

The primary objective of phase 2A is to determine the highest safe and well-tolerated dose of benfotiamine (600 mg or 1200 mg), as evaluated by the rate of tolerability events (TEs), for advancement to long-term 72 week exposure.

3.2.2 Secondary Objective

The secondary objective of phase 2A is to evaluate other measures of safety and tolerability in multiple doses of benfotiamine (600 mg or 1200 mg).

3.3 Phase 2B

3.3.1 Primary Objective

The primary objective of phase 2B is to assess efficacy of benfotiamine on global function and cognition over 72 weeks.

3.3.2 Secondary Objectives

The secondary objectives of phase 2B are:

- To evaluate longer-term safety and tolerability of benfotiamine treatment over 72 weeks.
- To evaluate the effects of benfotiamine and its PK relationships to primary clinical outcome measures, imaging measures and PD biomarkers of AD (plasma thiamine, and thiamine esters in whole blood, including thiamine diphosphate (ThDP) and thiamine monophosphate (ThMP)), over 72 weeks.
- To evaluate preservation or potential improvement in everyday activities of daily living, as well as changes and potential benefits on other measures of cognitive function.

3.3.3 Exploratory Objectives

The exploratory objectives of phase 2B are:

- To evaluate the downstream biological effects of treatment with benfotiamine on measures of neurodegeneration (cortical thickness MRI, neurofilament light chain, total

tau), neuroinflammation (glial fibrillary acid protein) and AD pathophysiology (p-tau 231, Aβ 42/40) over 72 weeks.
- To evaluate the effects of benfotiamine on neuropsychiatric symptoms and measures of cognition and function, including cognitive-functional composite measures, over 72 weeks
- To evaluate the effects of benfotiamine on measures of cognition administered remotely, and to compare the sensitivity of remote and in-person cognitive assessments over 72 weeks.

4 ENDPOINTS

4.1 Phase 2A

4.1.1 Primary Endpoint

The primary safety outcome in phase 2A is the rate of tolerability events (TEs) compared between active and placebo arms, at each dose.

A TE is defined as any of:
- Participant early discontinuation of IP related to intolerability
- A post-randomization moderate or severe AE that is possibly, probably or definitely related to IP

4.1.2 Secondary Endpoints

Secondary safety and tolerability endpoints for phase 2A include:
- AEs summarized by grade, attribution, and body systems, by number of events and by participant
- Physical exam abnormalities
- Vital signs (weight, blood pressure, pulse rate, temperature, respiration rate)
- Standard 12-lead resting ECG measures
- Summaries of safety laboratory tests
- Participant withdrawal rates
- Drug discontinuation rates

4.2 Phase 2B

4.2.1 Primary Endpoints

The primary cognitive endpoint is the within-participant change from baseline to 72 weeks compared between active arm and placebo on the ADAS-Cog-13.

The primary functional endpoint is the within-participant change from baseline to 72 weeks compared between active arm and placebo on the CDR-SB.

4.2.2 Secondary Endpoints

4.2.2.1 Safety and Tolerability

Secondary safety and tolerability endpoints for phase 2B include:

- AEs summarized by grade, attribution and body systems, by number of events and by participant
- Physical exam abnormalities
- Vital signs (weight, blood pressure, pulse rate, temperature, respiration rate)
- Standard 12-lead resting ECG measures
- Summaries of safety laboratory tests
- Participant withdrawal rates
- Drug discontinuation rates

4.2.2.2 PK Endpoints

PK measures of thiamine and its esters thiamine diphosphate (ThDP), thiamine monophosphate (ThMP), as well as the ability of ThDP to activate transketolase will be assessed as blood markers of efficacy of drug delivery measured by blood samples collected at baseline, week 8, and week 72.

4.2.2.3 Secondary Efficacy Endpoints

Additional efficacy endpoints of benfotiamine will be assessed by the within-participant change from baseline to week 72 between active and placebo on the following:

- The Alzheimer's Disease Cooperative Study – Activities of Daily Living Scale for use in Mild Cognitive Impairment (ADCS-ADL-MCI) to evaluate preservation or potential improvement in everyday activities of daily living
- Montreal Cognitive Assessment (MoCA) to evaluate changes and potential benefits in cognitive function

4.2.3 Exploratory Endpoints

Exploratory endpoints in phase 2B are the within-participant longitudinal change between active and placebo in:

- Volumetric measures of hippocampal, whole brain, and ventricles as well as regional cortical thickness measured by cranial MRI
- Neuropsychiatric Inventory (NPI) to evaluate changes and potential benefits in neuropsychiatric symptoms
- Neuropsychological Test Battery (NTB)
    - The following NTB subtests will be conducted remotely:
        - Rey Auditory Verbal Learning Immediate Recall
        - Rey Auditory Verbal Learning Delayed Recall
        - Number Span Forward

        - Number Span Backward
        - Category Fluency (average of Animal and Vegetable Fluency)
        - Letter Fluency (F & L)
    - The following NTB subtests will be conducted in-person:
        - Trail Making A
        - Trail Making B
        - Digit Symbol Substitution
        - Boston Naming Test (30 item)
- ADAS-Cog-Exec
- The plasma biomarkers Aβ42/Aβ40 ratio, total tau, p-tau 231, NfL and GFAP
- Increased advanced glycation end products (AGE) levels measured in plasma as an exploratory endpoint of PD.
    - AGE levels will be measured on plasma sample and four AGEs by mass spectrometry (N(6)-carboxymethyl-lysine (CML), pentosidine, N(6)-carboxymethyl-lysine (CEL), methylglyoxal-derived hydroimidazolone) will be quantified to measure all specific modes of action of benfotiamine.
- PK relationships to PD biomarkers and to clinical outcome measures.

Exploratory endpoints will be analyzed by disease severity groups, that is, by MCI due to AD and Mild dementia due to AD.

5 ETHICS AND REGULATORY CONSIDERATIONS

5.1 Good Clinical Practice

This study will be conducted in accordance with:

- Principles of the Declaration of Helsinki (revised version of Fortaleza, Brazil October of 2013).

- Good Clinical Practice (GCP) guidelines, as defined by the International Conference on Harmonization (ICH) Guideline, Topic E6(R2), the United States Code of Federal Regulations, Title 21, Part 50 (21CFR50) – Protection of Human Participants and Part 56.

- Institutional Review Boards (IRBs), Health Insurance Portability and Accountability Act (HIPAA), and all other applicable local regulatory requirements and laws.

Study personnel involved in conducting this study will be qualified by education, training and experience to perform their respective task(s) in accordance with GCP.

5.2 Institutional Review Board

Review and approval of this protocol and the associated informed consent documents and recruitment materials will be conducted by a central Institutional Review Board (IRB) registered with the Office for Human Research Protections (OHRP). Participating sites are required to rely

on the selected central IRB for this trial, Advarra. The study will not commence at any site until written approval for investigational product release and approval to enroll is obtained from ADCS Regulatory Affairs.

The investigator must obtain approval from the central IRB for all protocol amendments and, when warranted, changes to the informed consent document. Protocol and Informed Consent Form (ICF) amendments can be made only with the prior approval of the ADCS. The investigator may not implement any protocol deviation except when necessary to eliminate an immediate hazard to study participants, or when change(s) involve only logistical or administrative aspects of the trial, i.e. change of monitor(s) or telephone number(s) (ICH 4.5.2). The investigator shall notify the central IRB of deviations from the protocol or serious adverse events occurring at the site, in accordance with IRB and local policies and procedures.

5.3 Informed Consent and HIPAA Compliance

No study-specific procedure will be undertaken on an individual patient until that patient or the patient's legally authorized representative (LAR) has given written informed consent to take part in the study. The study partner must also participate in the consenting process.

It will be made clear to each potential participant, their LAR if applicable, and study partner that informed consent may be withdrawn at any time without needing to give a reason and that such withdrawal will not compromise the relationship between the patient and the Investigator nor the patient's future treatment.

Informed consent will be obtained in accordance with US 21 CFR 50.25 and ICH Good Clinical Practice. Applicable HIPAA privacy notifications will be implemented, and HIPAA authorizations signed before protocol procedures are carried out. Information should be given in both oral and written form.

Consent forms must be in a language fully comprehensible to the prospective participants and/or their LARs, and study partners, and ample opportunity must be given to inquire about the details of the study. Prior to a patient's participation in the trial, the written informed consent form should be signed and personally dated by the patient or by the patient's LAR, and by the person who conducted the informed consent discussion. Patients or their LAR must be provided a copy of the signed ICF. Study partners may also be required to sign the informed consent prior to study participation at the discretion of the responsible IRB reviewing this research.

The consent for storage will include consent to access stored data, biological samples, and imaging data for secondary analyses. Consent forms will specify that DNA and biomarker samples are for research purposes only; the tests on the DNA and biomarker samples are not diagnostic in nature and participants will not receive results.

Abnormal findings during the study including clinical laboratory results and MRI scan findings of clinical significance can be shared with participants or their treating physician per site clinician discretion with consent of the participant.

5.4 Patient Confidentiality │ HIPAA

Information about study participants will be kept confidential and managed according to the requirements of HIPAA. Those regulations require a signed patient HIPAA Authorization informing the patient of the following:

- What protected health information (PHI) will be collected from participants in this study
- Who will have access to that information and why
- Who will use or disclose that information
- The rights of a research patient to revoke their authorization for use of their PHI

If a participant revokes authorization to collect or use PHI, the investigator, by regulation, retains the ability to use all information collected prior to the revocation of participant authorization. Each site PI, under the guidance of his/her IRB, is responsible for ensuring that all applicable HIPAA regulations and state laws are met.

5.5 Potential Risks and Benefits Associated with this Study

5.5.1 Potential Risks

Risks associated with study participation are the potential for adverse reactions to the IP (see Section 1.3.1), concomitant medications, invasive study assessments like blood draws and lumbar puncture, and risks related to the process of undergoing brain MRI scans and neuropsychological testing.

5.5.2 Potential Benefits

There is no anticipated direct benefit to participants in this clinical trial and its related research, although there may be a symptomatic benefit. However, the results of this trial will provide evidence to support or refute the potential of therapeutic benefit from benfotiamine in AD and/or related disorders. This could make a major contribution towards the approval of new treatments for AD, which could have a substantial impact on public health. Also, participation in trials may enhance the public's understanding of interventions aimed at preventing or treating symptoms of AD and related disorders, and therefore, benefit future patients with AD or at risk for AD.

6 INVESTIGATIONAL PRODUCT

6.1 Investigational Product

Benfotiamine capsules, 300 mg, 600 mg, or placebo are opaque dark green (chlorophyll) colored capsules that are intended for oral administration in investigational studies in humans. In the placebo group, the active compound benfotiamine will be replaced with microcrystalline cellulose. The other components, shape and color are identical to the treatment. The investigational products will be packaged in high-density polyethylene (HDPE) bottles, 70 capsules per bottle, with child-resistant closures.

Investigational product (IP) will be provided from Advanced Orthomolecular Research Inc. (AOR) 3900 12th St NE, Calgary, AB, Canada, T2E. AOR will provide full testing on preparation of placebo and benfotiamine, including verification that the capsules are microbial free and not contaminated with heavy metals. They will also provide testing on potency to verify that active capsules will have 300mg and 600mg benfotiamine and placebo will contain non-detectable benfotiamine.

6.1.1 Investigational Product Intake

Participants are required to take the assigned number of capsules twice a day (BID; once in the morning and once in the evening). The first capsule intake will occur in person during the participant's baseline clinic visit. Blood samples for PK measures will be taken two hours (+/- 30 minutes) later. The intake of the second dose should occur later on the same day of the baseline visit. Participants should then continue with the twice daily treatment regimen, taking the capsules at approximately the same time of day throughout the study, until directed otherwise by the treating investigator or other qualified study personnel. Capsules are to be swallowed whole and not to be crushed or broken.

Participant drug supply will be examined in clinic during study visits to assess compliance overall and in the last week. Sites must calculate IP compliance upon its return at each study visit.

6.2 Coding, Packing and Labeling

Benfotiamine (300 mg or 600 mg) or placebo capsules will be contained in bottles. Participants will receive a new supply for each dosing period between study visits.

The IP packaging will be labeled with a unique identifier for dispensing and drug accountability. Labels will be in accordance with all applicable regulatory requirements. Labels will contain the drug name, study number, storage conditions and a caution statement that the drug is for clinical investigational use only. The dosing schedule and storage requirements will be clearly explained to the participants before dispensing the IP, and it will be printed on the labels.

The IP will be securely stored at the study site in accordance with the conditions specified on the label, separately from other drugs. The IP may not be used for any purpose other than this study.

6.3 Randomization and IP Ordering System

In phase 2A of the trial, each participant will be randomly allocated in a 1:1:1 ratio into one of the 3 groups: 1200 mg/day benfotiamine, 600 mg/day benfotiamine or placebo. The randomization stratification factor will be site. At the end of phase 2A, the highest safe and well-tolerated dose of benfotiamine will be recommended to be carried forward to the phase 2B efficacy studies.

At the start of phase 2B, all participants enrolled in the two phase 2A active dose arms will receive a new supply of benfotiamine at the selected phase 2B dose. All participants enrolled in the phase 2A placebo arm will also receive a new supply of placebo. Newly enrolled

participants will be randomized 1:2 active arm to placebo arm until parity is achieved between numbers of active and placebo arm participants (approximately 150 participants total), randomized 1:1 thereafter (approximately 100 participants). The randomization will be stratified by site.

A randomization schedule will be generated and incorporated into an electronic Randomization and Trial Supplies Management (RTSM) system and the treatment group will be assigned as the site randomizes the participant.

To complete participant randomization, the Investigator will use the study Electronic Data Capture (EDC) system to enter screening information on each participant. The Investigator and ADCS must each confirm study eligibility in the EDC system prior to randomization. Participants will be randomized at the baseline visit, once screening is completed and it is determined that the participant is eligible for the study. For those participants who qualify, the system will issue a IP kit number. Participants who are randomized but drop out prior to IP exposure will be replaced.

6.4 Phase 2A Dose Decision

To evaluate safety and tolerability, phase 2A of the trial will randomize approximately 150 participants total 1:1:1 to treatment with 1200 mg/day benfotiamine, 600 mg/day benfotiamine or placebo. The randomization stratification factor will be site.

The phase 2A primary outcome will be the rate of TEs (see section 4.1.1), compared between active and placebo arms. The phase 2A primary analysis will compare the rate of tolerability events (TEs) between the 1200 mg/day arm and the placebo arm. The analysis will test whether the rate of TEs is unacceptably high in this high-dose arm compared to placebo, using a formal one-sided hypothesis test of equal rates, against the one-sided alternative that the high dose arm TE rate is greater than the placebo arm TE rate (see section 12.1.1 Statistical Methods).

The phase 2A dose selection analysis will be conducted when 160 person-months of exposure have accumulated in each of the high dose and the placebo arm, or alternatively when 21 total TEs have been observed across the high dose arm and the placebo arm combined, whichever is earlier.

If the high dose arm has a significantly elevated TE rate, then the 600 mg/day dose arm will be taken forward to phase 2B and tested against placebo for the duration of the study.

At the expected time of the dose selection analysis, approximately 30 participants in each arm will have been on treatment for 12 weeks or more according to our enrollment projections.

At the time of the phase 2A analysis, data will be locked and the unblinded safety statistician will prepare a report giving the details of the analysis and providing a full unblinded safety report. The final recommendation on dose decision will be made by the DSMB.

6.5 Missed Doses

Participants will be provided with a diary for tracking daily intake and timing of dosing. If a

participant misses taking a dose of IP at their normal time in the morning, the dose can be taken anytime within the morning hours (i.e. before noon). Otherwise, the dose should be skipped (i.e., should not be made up) and the dosing resumed **in the evening of that same day** at the normal time and dosage.

If a participant misses an evening dose of IP, the dose should be skipped (i.e., should not be made up) and dosing resumed **on the morning of the following day** at the normal times and dosage.

6.6 Blinding

This is a double-blind placebo-controlled trial. Participants and study personnel will be blinded to both treatment arm (IP or placebo) and dosage (300 mg BID, 600 mg BID). The blind will be maintained by use of matching placebo.

Sites will be informed when the dose decision in phase 2A is determined and when this selected optimal dose is to be implemented for phase 2B. At that time, study personnel will be so notified but will remain blinded to treatment arm (IP or placebo). A new supply of IP will be provided to all participants in the study.

Only in the case of an emergency, when knowledge of whether the participant has received the investigational product is essential for the clinical management or welfare of the participant, may the Investigator unblind a participant’s treatment assignment. Procedures for emergency unblinding are initiated by contacting the ADCS Medical Monitor.

6.7 Drug Accountability

The Investigator or his/her designated representatives will dispense IP only to participants enrolled in the study. At each study visit, participants should bring in all unused drug and empty or partially full containers.

The Investigator (or, as appropriate, pharmacist/individual who is designated by the Investigator/institution) must maintain records of the delivery of the IP to the trial site, the inventory at the site, the use by each participant, and the destruction or return of unused IP. Destruction at the study site must be performed in accordance with the participating site standard operating procedures (SOP), and with prior approval by the ADCS.

7 PATIENT SELECTION AND CONCOMITANT MEDICATIONS

7.1 Inclusion Criteria

1. Aged 50 to 89 (inclusive) at screening
2. Mild Cognitive Impairment (MCI) due to AD or Mild dementia due to AD according to workgroups of the Diagnostic Guidelines of the National Institute on Aging and Alzheimer's Association (NIA-AA) (see Appendix II)
3. Mini-Mental State Examination (MMSE) score 20-30 inclusive at screening

4. Montreal Cognitive Assessment score (MoCA) < 26 at screening
5. Clinical Dementia Rating (CDR) global score of 0.5 or 1 with memory score of greater or equal to 0.5 at screening
6. Positive plasma AD biomarker signature:
    a. Plasma test (C_2N PrecivityAD2) that incorporates Aβ1-42 and Aβ1-40 and their ratio, age and ptau-217 into its predictive model. To qualify participants will have a High Amyloid Probability Score (APS2).
    b. For participants who are clinically symptomatic and who have been treated with anti-amyloid therapy, and no longer meet the threshold for High APS according to the PrecivityAD2 test, a previous positive AD biomarker result, including amyloid Positron-Emission Tomography (PET) scan with an FDA approved agent and a formal read, or AD fluid biomarker result of CSF or plasma consistent with the laboratory-based criteria for that particular assay, are eligible for inclusion.
7. Participants who are treated with FDA-approved acetylcholinesterase inhibitors (AchEI) and/or memantine may participate, but must be on a stable dosage regimen for at least 3 months prior to screening. Participants are expected to remain on a stable dosage regimen of these medications for the duration of the trial.
    a. Participants who are not being treated with FDA-approved AchEI and/or memantine at the time of screening are also eligible for inclusion, if it is expected that they will not be treated with these medications for the duration of the trial. AchEIs and/or memantine may only be initiated as clinically indicated during the study when a participant is not already on these medications at the outset, and will be documented accordingly
    b. Participants that are treatment naïve because AchEIs and memantine are not FDA-approved for MCI will not be excluded on this basis.
8. Participants must have a study partner who has frequent interaction with them (approximately >3-4 times per week), will be available for all clinic visits in person or remotely, and can assist in compliance with study procedures.
9. Female participants must be post-menopausal for at least one year or surgically sterile (bilateral tubal ligation, hysterectomy, or bilateral oophorectomy) for at least 6 months prior to screening.
10. Fluent in English or Spanish to ensure compliance with cognitive testing and study visit procedures.
11. Living in the community (includes assisted living facilities but excludes long-term care nursing facilities).
12. Ambulatory, or able to walk with an assistive device, such as a cane or walker.

13. Provision of informed consent from the participant (or the participant's legally authorized representative (LAR) if lacking decisional capacity), as well as study partner consent.

7.2 Exclusion Criteria

1. Significant neurological disorder other than AD including hypoxia, stroke, traumatic brain injury.
2. Diagnosis of uncontrolled depression or major depression, as determined by DSM-IV, unless successfully treated .
3. Any other major psychiatric disorder that impairs cognition or impacts participation in the study is exclusionary.
    a. Participants with major psychiatric disorders (e.g., severe GAD, bipolar disorder, schizophrenia etc.) where the condition is felt to be the most significant contributor to the cognitive impairment should be excluded.
4. Active medical conditions that impair cognition or impact participation in the study, other than AD should be exclusionary.
    a. Participants with medical conditions (e.g. severe OSA) in whom the condition is felt to be the most significant contributor to the cognitive impairment should be excluded.
5. Significant neurodegenerative diseases, other than AD, and causes of dementias, including Parkinson's disease and Huntington's disease, vascular dementia, CJD (Creutzfeldt-Jakob disease), LBD (Lewy Body dementia), PSP (Progressive Supranuclear Palsy), AIDS (Acquired Immunodeficiency Syndrome), or NPH (normal pressure hydrocephalus).
6. Meeting Diagnostic Criteria for Possible AD according to workgroups of the Diagnostic Guidelines of the NIA-AA (2011).
7. A current diagnosis of uncontrolled Type I or Type II diabetes mellitus, as defined by Hemoglobin A1 C (Hb A1C $\geq$ 8).
8. Current active, uncontrolled seizure disorder.
9. Participation in another clinical trial for an investigational agent and having taken at least one dose of IP, unless confirmed as having been on placebo
    a. For trials of symptomatic medications, participation is excluded within 4 weeks prior to the screening visit.
    b. For trials of disease-modifying medications, participation is excluded within 6 months of the screening visit.
    c. The end of a previous investigational trial is defined as the date of the last dose of an investigational agent.
10. Previous exposure to Benfotiamine within past 3 months.

11. Current serious or unstable illness including cardiovascular disease, resistant hypertension, hepatic, renal, gastroenterologic, respiratory, endocrinologic, neurologic, psychiatric, immunologic, or hematologic disease or other conditions that, in the investigator’s or sponsor’s opinion, could interfere with the interpretation of safety and assessment of efficacy in this study.
12. Diagnosis of cancer, except for those participants who have undergone potentially curative therapy with no evidence of recurrence for >5 years.
13. History of alcoholism or substance abuse, current or within past 5 years.
14. Contraindication to MRI, including but not limited to:
    a. Clinical history or examination finding that, in the judgment of the investigator and/or the local radiologist, would pose a potential safety risk to the participant being considered for an MRI.
    b. Implant devices not compatible in the magnetic resonance environment, such as: Automatic Implanted Cardioverter Defibrillators (AICDs); cochlear implants; cerebral aneurysm clips; implanted infusion pumps; implanted nerve stimulators; metallic splinters in the eye; other magnetic, electronic, or mechanical implants; Pacemakers are permitted only if labeled as conditional cardiac implantable electronic devices (CIEDs), have been approved for MRI use by the FDA, verified by the pacemaker’s manufacturer as approved for MRI use, and determined to be suitable by the site’s radiologist and principal investigator.
15. Cranial MRI at screening shows evidence of infection, tumor, cortical infarction, or multiple lacunes in prefrontal or critical memory regions as determined by the site PI with a local reading; inconclusive findings may be subject to review by the ADCS Imaging Core.
16. Initiation of a monoclonal antibody treatment targeting brain amyloid (including Leqembi, Aduhelm, Solanezumab, Donanemab) within 6 months prior to the screening visit.
    a. For FDA approved monoclonal antibodies targeting brain amyloid, participants must be on stable dose of treatment for at least 6 months prior to screening.
17. A disability that may prevent the patient from completing all study requirements in the opinion of the PI.

7.3 Concomitant Medications

7.3.1 Concomitant AD Medication

Eligible participants being treated with FDA-approved AchEIs and/or memantine at the time of screening are expected to remain on a stable dosage regimen of these medications for the duration of the trial.

AchEIs and/or memantine may only be initiated as clinically indicated during the study when a participant is not already on these medications at the outset, and will be documented accordingly.

For FDA approved monoclonal antibodies targeting brain amyloid, participants must be on stable dose of treatment for at least 6 months prior to screening.

7.3.2 Other Prohibited Concomitant Medications

Use of the following medications is prohibited throughout participation in the study:

- Should not use thiamine or benfotiamine supplements outside of a multivitamin
- Treatment with immunosuppressive medications (e.g. systemic corticosteroids in a dose equivalent to more than 10 mg of prednisone/day) within the last 90 days prior to baseline. Topical and nasal corticosteroids and inhaled corticosteroids for asthma are permitted.
- Treatment with chemotherapeutic agents for malignancy within the last year prior to baseline.
- Any concomitant treatment which may impair cognitive function requires a washout phase of at least 5 half-lives of the treatment prior to screening.

The following requirements apply to all other medications not intended to treat AD:

- Participants must be on stable dose for at least 4 weeks prior to baseline, except for medications which are administered as short courses of treatment (e.g. anti-infective) or which are to be used as needed (PRN).
- Medications which are central nervous system active (e.g. hypnotics) and may affect cognitive function are not permitted during a period of 72 hours prior to neuropsychological testing.
- Participants who initiate treatment or undertake dose adjustment with drugs not intended for treatment of cognitive impairment during the study may continue in the study if in the opinion of the investigator this will not interfere with study procedures or safety.

Prospective participants who can safely discontinue these medications for 4 weeks before screening and remain off these medications throughout the study, can be considered for inclusion in the study.

If, during the clinical study, the administration of a non-permitted concomitant medication becomes necessary, the ADCS Medical Monitor must be contacted to determine if the participant needs to be prematurely discontinued from study treatment (see Section 10).

7.3.3 Prohibited Concomitant Medications for Optional Lumbar Puncture

Participants who are actively being treated with low dose acetylsalicylic acid (ASA) may undergo lumbar puncture, at the discretion of the investigator. Participants who are actively being treated with dual antiplatelet therapy or with any anticoagulation medications (e.g. heparin, warfarin, thrombin inhibitors, Factor Xa inhibitors) should **not** undergo lumbar puncture. If not contraindicated, participants who are being treated with low dose ASA and/or other antiplatelet/anticoagulant medications may have these medications held for an appropriate period

of time (based on the half-life of the medication and/or pharmacodynamic effects on hematological parameters), at the discretion of the investigator, prior to performing lumbar puncture. Stop and start dates must be documented on the appropriate Concomitant Medication case report form (CRF). Platelet counts should be >100,000, and PTT and INR levels must be returned to normal before the lumbar puncture is performed. The ADCS Medical Monitor should be contacted if there are any questions.

8 STUDY PROCEDURES

8.1 Study Visits

The schedule of study visits and procedures to be performed at each visit are outlined below and a table can be found in Appendix I.

8.1.1 Screening (within 42 days prior to baseline)

Participants who the Investigator considers to be appropriate for the study, will have the study explained to them, their LAR if applicable, and their study partner by the Investigator or his/her designee and will be given a copy of the written Informed Consent Form (ICF). When they have had sufficient time to study this information and the opportunity to ask any questions they wish, they will be invited to give their consent to participation by signing the ICF. The study partner may also be required to sign the ICF at the discretion of the responsible IRB reviewing this research.

Once the participant or LAR (and study partner if required) have given consent, the following procedures will be undertaken during the screening period.

- Confirmation of MCI due to AD or Mild dementia due to AD according to workgroups of the Diagnostic Guidelines of the NIA-AA (see Appendix II).
- MMSE
- MoCA
- CDR
- Documentation of demographics, medical history and education
- Modified Hachinski Ischemic Scale (MHIS)
- Neurological exam
- Physical examination
- Vital signs (blood pressure, pulse, temperature, respiration rate)
- Weight
- Height
- Documentation of concurrent pathologies and concomitant treatment
- Documentation of adverse events from time of consent
- Blood sample collection for:
    - C_2N PrecivityAD2 test (see Section 8.3.1)
    - Clinical safety laboratory tests (see Section 8.2)
        - Hematology

        - Chemistry
    - Infectious Disease Serology
        - Human Immunodeficiency Virus (HIV), Hepatitis A Virus (HAV), Hepatitis B Virus (HBV), Hepatitis C Virus (HCV), Rapid Plasma Reagin (RPR, syphilis)
    - Other Screening Tests
        - Thyroid Stimulating Hormone (TSH), hemoglobin A1c (HbA1c)
- Urinalysis (see section 8.2)
- Resting 12-lead ECG
- Cranial MRI per protocol imaging requirements
    - If a participant has not had an MRI performed within 6 months of screening (i.e. within 6 months from the date of informed consent), then an MRI must be performed as part of the screening requirements for this study, per the imaging protocol, and should be one of the last screening procedures performed to determine final eligibility in order to prevent patients from undergoing unnecessary MRIs.
    - If a participant has had an MRI within 6 months of screening (i.e. within 6 months from the date of informed consent) but the MRI does not follow the study-specific imaging protocol, that MRI can be used to help determine eligibility; however, another MRI must be performed per the imaging protocol, and must occur as close to, and prior to, the baseline visit, after all other eligibility criteria have been confirmed.
- Columbia Suicide Severity Rating Scale (C-SSRS)
- CAGE-AID Substance Abuse Screening Tool

Following completion of all screening assessments, results for participants who meet all inclusion and none of the exclusion criteria will be submitted via the study EDC system. The Investigator must confirm all study eligibility criteria are met prior to participant randomization.

8.1.1.1 Re-screening

Re-screening can be undertaken when the reason for screen failure has been identified and corrected. A full re-screen (re-consent participant, assign new Participant ID and perform all screening assessments) may be conducted if more than twelve weeks have passed since the last assessment of screen failure.

If the participant screen fails a second time, any further re-screening must be discussed on a case-by-case basis with ADCS Medical Safety and approved.

For re-screening, vMRI taken at the previous screening (screen failure) can be used if it will be less than 9 months from the time of the scan used for the initial screening period (i.e., a total screening window allowance of 9 months for this procedure from the date of initial informed consent).

8.1.2 Baseline (Week 0)

At baseline, eligible participants will be randomized into the study. Each will undergo the following procedures **prior** to first dose of IP:

- Verification of Inclusion/Exclusion criteria
- Randomization in EDC
- Physical exam
- Vital signs (blood pressure, pulse, temperature, respiration rate)
- Weight
- Documentation of concomitant treatment
- Documentation of adverse events
- Blood samples for:
    - Safety laboratory tests (hematology and serum chemistry, see Section 8.2)
    - PK analysis (thiamine level and its esters ThDP and ThMP, and ThDP activation of transketolase) (see Section 8.3)
        - Blood will be collected once just prior to the participant receiving their morning dose of IP at baseline and again 2 hours (+/- 30 minutes) after this morning dose at baseline.
        - Date and time of PK sampling and of IP intake on the day of visits and day prior should be documented, along with time of last meal
    - PD analysis (AGE) (see Section 8.3)
        - Blood will be collected once just prior to the participant receiving their morning dose of IP at baseline.
        - Date and time of PD sampling and of IP intake on the day of visits and day prior should be documented, along with time of last meal
    - Biomarker analysis (total tau, p-tau-231, NfL, GFAP)
    - Biobanking of specimens for future research
- Urinalysis (see Section 8.2)
- C-SSRS
- ADAS-Cog-13
- NPI
- ADCS-ADL-MCI
- NTB-Remote, 6-item (see Section 9.1.1)
- NTB-In-Person, 4-item (see Section 9.1.1)
- Research Satisfaction Survey
- ***Optional*** lumbar puncture for cerebrospinal fluid (CSF) biobanking
- Post lumbar puncture safety telephone follow-up (1 to 3 days after lumbar puncture)

Participants will receive their first (morning) dose of IP in clinic during the baseline visit. Following the completion of all remaining baseline visit procedures, participants will receive a supply of IP to take home with instructions on how to take it at home until their next clinic visit. Participants will be given an appointment to return at week 4 (± 7 days).

8.1.3 Week 4 (± 7 days)

All participants returning for week 4 will undergo the following procedures:

- Physical exam
- Vital signs (blood pressure, pulse, temperature, respiration rate)

- Weight
- Documentation of concomitant treatment
- Documentation of adverse events
- Blood samples for:
    - Safety laboratory tests (hematology and serum chemistry, see Section 8.2)
- C-SSRS
- Compliance check (pill count)

At this visit, participants will receive a supply of IP to take home with instructions on how to take it at home until their next clinic visit. Participants will be given an appointment to return in 4 weeks (± 7 days).

8.1.4 **Week 8 (± 7 days)**

All participants returning for week 8 will undergo the following procedures:

- Physical exam
- Vital signs (blood pressure, pulse, temperature, respiration rate)
- Weight
- Documentation of concomitant treatment
- Documentation of adverse events
- Blood samples for:
    - Safety laboratory tests (hematology and serum chemistry, see Section 8.2)
    - PK analysis (thiamine and its esters ThDP and ThMP, and ThDP activation of transketolase) (see Section 8.3)
        - Blood will be collected once just prior to the participant receiving their routine morning dose of IP at Week 8 and again approximately 2 hours (+/- 30 minutes) after their morning dose at Week 8.
        - Date and time of PK sampling and of IP intake on the day of visits and day prior should be documented, along with time of last meal.
    - PD analysis (AGE) (see Section 8.3)
        - Blood will be collected once just prior to the participant receiving their routine morning dose of IP at Week 8.
        - Date and time of PD sampling and of IP intake on the day of visits and day prior should be documented, along with time of last meal.
- C-SSRS
- Compliance check (pill count)

At this visit, participants will receive a supply of IP to take home with instructions on how to take it at home until their next clinic visit. Participants will be given an appointment to return in 4 weeks (± 7 days).

8.1.5 **Week 12 (± 7 days)**

All participants returning for week 12 will undergo the following procedures:

- Physical exam
- Vital signs (blood pressure, pulse, temperature, respiration rate)
- Weight
- Documentation of concomitant treatment
- Documentation of adverse events
- Blood samples for:
    - Safety laboratory tests (hematology and serum chemistry, see Section 8.2)
- C-SSRS
- Compliance check (pill count)

At this visit, participants will receive a supply of IP to take home with instructions on how to take it at home until their next clinic visit. Participants will be given an appointment to return in 12 weeks (± 7 days).

8.1.6 Week 24 (± 7 days)

All participants returning for week 24 will undergo the following procedures:

- Physical exam
- Vital signs (blood pressure, pulse, temperature, respiration rate)
- Weight
- Documentation of concomitant treatment
- Documentation of adverse events
- Blood samples for:
    - Safety laboratory tests (hematology and serum chemistry, see Section 8.2)
- Resting 12-lead ECG
- C-SSRS
- MoCA
- CDR-SB
- ADAS-Cog-13
- NPI
- ADCS-ADL-MCI
- NTB-Remote, 6-item (see Section 9.1.1)
- NTB-In-Person, 4-item (see Section 9.1.1)
- Research Satisfaction Survey
- Compliance check (pill count)

At this visit, participants will receive a supply of IP to take home with instructions on how to take it at home until their next clinic visit. Participants will be given an appointment to return in 12 weeks (± 7 days).

8.1.7 Week 36 (± 7 days)

All participants returning for week 36 will undergo the following procedures:

- Physical exam
- Vital signs (blood pressure, pulse, temperature, respiration rate)
- Weight
- Documentation of concomitant treatment
- Documentation of adverse events
- Blood samples for:
    - Safety laboratory tests (hematology and serum chemistry, see Section 8.2)
- C-SSRS
- Compliance check (pill count)

At this visit, participants will receive a supply of IP to take home with instructions on how to take it at home until their next clinic visit. Participants will be given an appointment to return in 12 weeks (± 7 days).

8.1.8 **Week 48 (± 7 days)**

All participants returning for week 48 will undergo the following procedures:

- Physical exam
- Vital signs (blood pressure, pulse, temperature, respiration rate)
- Weight
- Documentation of concomitant treatment
- Documentation of adverse events
- Blood samples for:
    - Safety laboratory tests (hematology and serum chemistry, see Section 8.2)
- C-SSRS
- MoCA
- CDR-SB
- ADAS-Cog-13
- NPI
- ADCS-ADL-MCI
- NTB-Remote, 6-item (see Section 9.1.1)
- NTB-In-Person, 4-item (see Section 9.1.1)
- Research Satisfaction Survey
- Compliance check (pill count)

At this visit, participants will receive a supply of IP to take home with instructions on how to take it at home until their next clinic visit. Participants will be given an appointment to return in 12 weeks (± 7 days).

8.1.9 **Week 60 (± 7 days)**

All participants returning for week 60 will undergo the following procedures:

- Physical exam

- Vital signs (blood pressure, pulse, temperature, respiration rate)
- Weight
- Documentation of concomitant treatment
- Documentation of adverse events
- Blood samples for:
    - o Safety laboratory tests (hematology and serum chemistry, see Section 8.2)
- C-SSRS
- Compliance check (pill count)

At this visit, participants will receive a supply of IP to take home with instructions on how to take it at home until their next clinic visit. Participants will be given an appointment to return in 12 weeks (± 7 days).

8.1.10 Week 72 or Early Termination (± 7 days)

All participants returning for week 72 will undergo the following procedures:

- Physical exam
- Vital signs (blood pressure, pulse, temperature, respiration rate)
- Weight
- Documentation of concomitant treatment
- Documentation of adverse events
- Blood samples for:
    - o Safety laboratory tests (hematology and serum chemistry, see Section 8.2)
    - o PK analysis (thiamine and its esters, ThDP, ThMP, and ThDP activation of transketolase) (see Section 8.3)
        - ▪ Date and time of PK sampling and of IP intake on the day of visits and day prior should be documented, along with time of last meal.
    - o PD analysis (AGE) (see Section 8.3)
        - ▪ Date and time of PD sampling and of IP intake on the day of visits and day prior should be documented, along with time of last meal.
    - o Biomarker analysis (Aβ42, Aβ40, total tau, p-tau-231, NfL, GFAP)
    - o Biobanking of plasma for future research
- Resting 12-lead ECG
- Cranial MRI (± 14 days)
- C-SSRS
- MoCA
- CDR-SB
- ADAS-Cog-13
- NPI
- ADCS-ADL-MCI
- NTB-Remote, 6-item (see Section 9.1.1)
- NTB-In-Person, 4-item (see Section 9.1.1)
- Research Satisfaction Survey
- Optional lumbar puncture* for CSF biobanking

- Post lumbar puncture safety telephone follow-up (1 to 3 days after lumbar puncture)
- Return of IP and compliance check (pill count)
- Treatment Blinding Questionnaire

*NOTE: PK samples should be drawn at the time of optional lumbar puncture. The lumbar puncture should be done ***after*** the MRI scan ***if*** performed on the same day. If the lumbar puncture is performed on a **separate day** from the MRI and occurs **before** the MRI, then there must be at least a 3-day window between the lumbar puncture and the MRI. If the lumbar puncture is performed on a **separate day** from the MRI and occurs **after** the MRI, then there is no window (waiting period) between the MRI and lumbar puncture.

Participants who terminate the study early will undergo a Post-Treatment Safety Follow-Up visit 4 weeks (± 7 days) following their Early Termination visit, and will undergo the same assessments as listed under section 8.1.10.

8.1.11 Week 76 or Post-Treatment Safety Follow-up (± 7 days)

A follow-up telephone call will be conducted for either week 76 or the Post-Treatment Follow-Up visit at 4 weeks (± 7 days) following their Early Termination visit to document adverse events and concomitant medications. This may be scheduled as a clinic visit if the PI deems necessary for the health and welfare of the participant.

8.1.12 Unscheduled Visits

In the event an active study participant returns to the site for medical care related to their participation in the study protocol, and beyond what is specified in the schedule of events, but deemed necessary for the health and welfare of the participant, this should be considered an unscheduled study visit.

During an unscheduled visit, documentation of concomitant medication, adverse events, and vital signs will be collected. Additional safety procedures may be completed at the discretion of the PI or at request of the medical monitor.

In addition, an unscheduled visit at the start of phase 2B may be required to replace IP supply in all active participants once the phase 2A dose selection is made.

8.2 Laboratory Safety Assessments

Clinical Safety Laboratory tests will be performed by the contracted central laboratory for the trial.

It is up to the investigator's discretion to assess the CSF for routine safety measurements, according to the appropriate local medical standards of the site.

Clinical Safety Laboratory assessments include the following:

Hematology: White Blood Cell Count (WBC) and differential count, Red Blood Cell Count (RBC), Hemoglobin (Hb), Hematocrit (HCT), Mean Corpuscular Volume (MCV), Mean Corpuscular Hemoglobin (MCH), Mean Corpuscular Hemoglobin, Concentration (MCHC), Platelet Count (PLT)

Chemistry: Sodium (Na), Potassium (K), Chloride (Cl), Bicarbonate, Blood Urea Nitrogen (BUN), Glucose, Calcium (Ca), Creatinine (Crn), Creatinine Kinase (CPK), total protein, Direct Bilirubin, Indirect Bilirubin, Total Bilirubin, gamma-glutamyl transferase (GGT), Magnesium, Phosphorus, Albumin, Glutamic Oxaloacetic Transferase (AST, SGOT), Glutamic-Pyruvate Transferase (ALT, SGPT), Alkaline Phosphatase NOS (ALP), Lactate Dehydrogenase (LDH), estimated Glomerular Filtration Rate (eGFR), uric acid, total cholesterol, LDL, HDL, triglycerides, B12 (screening), folate (screening)

Other (screening): Thyroid Stimulating Hormone (TSH), hemoglobin A1c (HbA1c)

Urinalysis: Color, Appearance, Specific Gravity, pH, Blood, Glucose, Protein, Ketones, Leukocyte Esterase, Nitrite, Urobilinogen, Bilirubin, Crystals, Casts, Debris.

Serology (screening): Human Immunodeficiency Virus (HIV), Rapid Plasma Reagin (RPR, syphilis)

The central laboratory will provide the investigational sites with all appropriate materials for specimen collection, sample processing, packaging and shipping. Full details of sampling (blood and urine), sample preparation and storage methods will be given in the laboratory manual

8.3 Plasma Assessments for PK, PD and Biomarkers

Plasma, whole blood, and red blood cells for PK, PD and Biomarker analyses will be collected in a uniform fashion into 10mL lavender top EDTA tubes at specified timepoints (see also Appendix I). Once blood is collected, it should be processed according to the process outlined in the National Centralized Repository for Alzheimer's Disease and Related Dementias (NCRAD) Manual of Procedures. Samples will be frozen at -80 ºC until shipment to the central repository biorepository, NCRAD, for storage and distribution to the appropriate teams responsible for carrying out the assays.

Plasma, whole blood, and red blood cells for PK and PD analyses will be collected at two collection timepoints during the baseline and Week 8 visits, once just prior to the morning dose and again approximately 2 hours (+/- 30 minutes) after the morning dose. Plasma, whole blood, and red blood cells for PK and PD analyses will be also collected at a single timepoint during the Week 72 visit.

Measures of thiamine and its esters, ThDP and ThMP, and the ability of ThDP to activate transketolase will be provided as blood markers of efficacy of drug delivery. These measurements will be conducted on whole blood and red blood cells by Drs. Albert Koulman and Kerry Jones at University of Cambridge (Cambridge, UK).

PD of increased advanced glycation end products (AGE) levels will be measured on plasma sample to quantify four AGEs by mass spectrometry to best measure all specific modes of action of benfotiamine. The AGEs N(6)-carboxymethyl-lysine (CML) and pentosidine are primarily derived from glucose, when the glucose environment is not strictly maintained. Two other AGEs, N(6)-carboxymethyl-lysine (CEL) and the abundant methylglyoxal-derived hydroimidazolone (N-(5-hydro-5-methyl-4-imidazolon-2-yl)-ornithine, MG-H1), arise from compounds produced during normal glycolysis and form methylglyoxal, a highly reactive AGE precursor that must be effectively detoxified. Dr. Sarah Flowers, at Georgetown University (Washington, DC) will provide an analysis of these four different measures of AGE.

Plasma biomarkers of neuroinflammation (GFAP), neurodegeneration (Neurofilament Light [NfL]), and changes in tau (total tau and p-tau 231) will be quantified by Simoa assay at the fluid biomarker unit at the University of Gothenberg, in the laboratory of Henrik Zetterberg.

Any unused portion of these samples will be stored at the central biorepository, NCRAD, for future research. DNA samples will also be stored at NCRAD for additional sample sharing that may include further genetic testing. Access to samples will be granted according to established SOPs.

Samples will be de-identified to preserve the confidentiality of participants. Participants can request in writing any time to have their samples destroyed.

Results of this testing are for research purposes only and will not be disclosed to the participant or study partner.

8.3.1 C$_2$N PrecivityAD2™ Test

C$_2$N PrecivityAD2™ is a plasma test that quantifies specific plasma amyloid beta and tau peptide concentrations to calculate the Aβ42/40 concentration ratio and p-tau217/np-tau217(p-tau217 concentration ratio) into its predictive model [68-71]. C2N's plasma Aβ42/40 measures have been analytically and clinically validated to identify brain amyloid pathology. These biomarker analyses will be conducted in C$_2$N Diagnostics' CLIA-certified, CAP-accredited, and ISO 13485 compliant laboratory (St. Louis, MO; C$_2$N Diagnostics) using their liquid chromatography tandem mass spectrometry (LC-MS/MS) based assays. The PrecivityAD2™ Test Results and Interpretation report includes the Amyloid Probability Score (APS2), a numerical value ranging from 0-100 that indicates whether a patient is Positive (high likelihood) or Negative (low likelihood) for the presence of brain amyloid plaques by amyloid PET imaging. For the purposes of this study, participants must have a high (>47.5) APS score.

8.4 Optional Lumbar Puncture and CSF Samples

An optional lumbar puncture (LP) will be performed at baseline and week 72 (End of Study) for collection of CSF. CSF samples should be collected at approximately the same time of day at each collection timepoint, and should occur between 2-4 hours after dosing on days where the participant receives IP. Within 1 to 3 days prior to the LP, a normal coagulation panel must be analyzed by the local lab to rule out a clotting disorder. Participants taking an anti-platelet agent (e.g., Plavix) must be discontinued from that agent for a minimum of 5 days prior to the LP.

These participants may continue with the agent after a minimum of 24 hours post LP. Participants who are taking anticoagulants (e.g., warfarin [Coumadin] and/or dabigatran [Pradaxa] may not undergo an LP and are not suitable to participate in this study.

It is up to the investigator's discretion to assess the CSF for routine safety measurements, according to the appropriate local medical standards of the site.

LP via image-guided techniques and/or collection of fluid via aspiration will be permitted, following approval from Clinical Operations. Site personnel should advise the participant that use of certain agents (e.g., fluoroscopy, x-rays) involves exposure to radiation. If these agents are used, details of the procedure and the risks associated with the additional use of radiation may need to be included in the site's Informed Consent.

Details of the CSF sampling are contained in the NCRAD Manual of Procedures.

Each study participant will be contacted by phone 1 to 3 days after the LP to confirm participant well-being and to discuss any adverse events.

An approximate CSF volume of 15 (preferred minimum) to 20 mL will be collected.

The collected CSF will be stored at the central biorepository, NCRAD, for future research and future analysis of CSF biomarkers. Access to samples will be granted according to established SOPs.

Samples will be de-identified to preserve the confidentiality of participants. Participants can request in writing any time to have their samples destroyed.

8.5 Physical and Neurological Examination

A physical examination will be performed by a medically qualified professional at every study visit. A review of the major body systems will be performed, and vital signs will be assessed. A neurological examination will only be performed at screening and will include assessment of motor strength, sensory, deep tendon, tremor, cerebellar, cranial, and mental status.

8.5.1 Vital Signs

Systolic and diastolic blood pressure, pulse, temperature, and respiration will be collected at screening and every scheduled study visit (see Appendix I).

8.6 Electrocardiogram

A standard 12-lead resting ECG will be performed during screening, week 24, and week 72. The Investigator or designee will review the 12-lead ECG and findings will be recorded in the electronic Case Report Form (eCRF) as normal, abnormal but not clinically significant, or abnormal and clinically significant. Any clinically significant abnormalities on ECGs recorded after administration of the investigational product will also be documented as AEs and entered

on the AE page of the eCRF. Clinically significant abnormalities on ECGs prior to administration of the investigational product, will be recorded as medical history.

8.7 Cranial MRI Assessments

Brain structural change is seen in normal aging, but is accelerated in neurodegenerative disease, including AD. Atrophy in AD arises from neuron and synapse loss that begins in the entorhinal cortex. The pathology then spreads throughout the limbic regions of the temporal lobe, including the hippocampal formation. Subsequently, neuron loss and atrophy are observed throughout neocortical association areas in temporal, parietal and frontal lobes.

Participants will undergo cranial MRI scans of the brain at screening and week 72. Neuroimaging assessments with cranial MRI will include volumetric measures of hippocampal, whole brain, and ventricles as well as regional cortical thickness.

Alzheimer's Disease Neuroimaging Initiative (ADNI)-quality, three dimensional T1-weighted structural images are acquired using MRI to assess for cortical thickness and volumetric change. These measures will all be undertaken with the same imaging protocol that will include a localizer scan, 3D T1-weighted sagittal acquisition (MPRGAE or IR-SPGR), a 3D T2-weighted FLAIR axial acquisition, a T2 gradient recalled echo axial acquisition and a diffusion weighted axial acquisition to assess for restricted diffusion. We will undertake a quantitative anatomic regional change (QUARC) that uses a nonlinear registration of longitudinal images to measure change in regions of interest including cortical thickness in those grey matter regions most sensitive to change in this disease stage. Detail for the statistical computations is given in the Statistical Analytical Plan (SAP).

Images will be checked for image quality and adherence to scanning protocols. 3D T1-weighted datasets passing quality checks will be corrected for spatial distortion and for intensity variation. Screening and follow-up datasets for each participant will be spatially registered to one another using rigid-body registration followed by nonlinear registration and neuroanatomic parcellation to quantify whole-brain and subregional volumetric change on a patient-by patient basis.

If performed on the same day as a lumbar puncture, the MRI should be conducted before the lumbar puncture. Otherwise, at least a 3-day window between MRI and the lumbar puncture is required. Scanners that have passed the study’s qualification procedures will be used. Participants must be scanned by the same scanner throughout the study.

Participants with a contraindication to MRI at the time of screening are deemed ineligible to participate in this study. Participants may continue to participate on the study if they have already been randomized but develop a contraindication to MRI during the course of the study.

9 STUDY-SPECIFIC INSTRUMENTS

9.1 Cognitive Measures

9.1.1 Neuropsychological Test Battery (NTB)

The (NTB) is comprised of the 10 measures listed below. A subset of 6 of these measures (NTB-Remote) will be administered remotely to allow for comparison against in-person cognitive testing with the ADAS-Cog 13. The NTB-Remote also represents a contingency for remote telehealth assessments if this accommodation is needed due to unforeseen circumstances (e.g., the COVID-19 pandemic). The remaining 4 measures will be conducted in-person (NTB-In-Person). NTB-Remote and NTB-In-Person assessments will be conducted at baseline, week 24, week 48 and week 72 (End of Study). The NTB-Remote will be administered over videoconference within 10 calendar days after each in-person visit (baseline, week 24, week 48 and week 72). Participants will use their own interactive video capable device for the NTB-Remote assessment. An interactive video capable device will be provided to participants who do not have one. Participants will be instructed to use the same device for all remote assessments.

NTB-Remote

- Rey Auditory Verbal Learning Immediate Recall
- Rey Auditory Verbal Learning Delayed Recall
- Number Span Forward
- Number Span Backward
- Category Fluency (average of Animal and Vegetable Fluency)
- Letter Fluency (F & L)

NTB-In-Person

- Trail Making A
- Trail Making B
- Digit Symbol Substitution
- Boston Naming Test (30 item)

9.1.1.1 NTB-Remote Assessments

9.1.1.1.1 Rey Auditory Verbal Learning Test Immediate and Delayed Recall

The Rey Auditory Verbal Learning Test is a 15-item list learning test that will be used to assess verbal learning and memory. [72] Testing consists of five learning trials, an interference list (single trial), and delayed recall and recognition memory of the initial word.

9.1.1.1.2 Number Span Forward and Backward

Number Span assesses two different working memory constructs: Forward Number Span measures the capacity for retaining information very briefly for the purpose of repeating it exactly, while Backward Number Span measures the ability not only to retain the information

but also to mentally manipulate the numbers and recite them in reverse sequence. [73] Numbers for both forward and backward span tests are presented with sequences ranging from 2 to 9 numbers. Two trials are administered at each sequence length. Two scores are reported for each task: number of correct trials and longest sequence repeated correctly prior to failing two consecutive trials of the same length.

9.1.1.1.3 Verbal Fluency – Category Fluency

Category fluency assesses semantic memory and language fluency. [74] Participants are asked to name as many different exemplars as possible within 60 seconds in each of two semantic categories: animals and vegetables.

9.1.1.1.4 Verbal Fluency – Letter Fluency

Letter Fluency is a measure of word generation that may be sensitive to dysfunction in the dominant frontal lobe. [74] Participants will be given 60 seconds to name exemplars that begin with each of two letters: F and L.

9.1.1.2 NTB-In-Person Assessments

9.1.1.2.1 Boston Naming Test (30-item)

An abbreviated version of the Boston Naming Test will be administered to assess visual confrontation naming. [75] Participants are asked to identify (i.e., name) 30 line drawings of objects (odd-numbered items from the 60-item Boston Naming Test).

9.1.1.2.2 Trail Making Test (Trails A and B)

The Trail Making Test is a test of processing speed and executive function. [76] Trail Making A requires participants to draw a line to connect 25 numbered circles in ascending numerical order as quickly as possible. Trail Making B requires participants to draw a line to connect 25 circles containing either numbers (1 through 13) or letters (A through L) in alternating and ascending order (e.g., 1 to A; 2 to B) as quickly as possible.

9.1.1.2.3 Digit Symbol Substitution

The Digit Symbol Substitution test assesses executive function and psychomotor speed. [77] Participants are asked to complete an array of symbol-digit pairings based upon a presented key as quickly as possible for 90 seconds.

9.1.2 ADAS Cognitive Subscale-13 (ADAS-Cog-13)

The ADAS-Cog-13 is a structured scale that evaluates memory (word recall, word recognition), reasoning (following commands), language (naming, comprehension), orientation, ideational praxis (placing letter in envelope) and constructional praxis (copying geometric designs). Ratings of spoken language, language comprehension, word finding difficulty, and ability to remember test instructions are also obtained. [78] The test is scored in terms of errors, with higher scores

reflecting poorer performance and greater impairment. Scores can range from 0 (best) to 70 (worst). The ADAS-Cog-13-item scale [79] includes all original ADAS-Cog items with the addition of a number cancellation task and a delayed free recall task, for a total of 85 points. The purpose of these additional items is to increase the number of cognitive domains and range of symptom severity without a substantial increase in the time required for administration.

9.1.3 Mini-Mental State Examination (MMSE)

The MMSE is a frequently used screening instrument for AD drug trials. It evaluates orientation, memory, attention, concentration, naming, repetition, comprehension, and ability to create a sentence and to copy two intersecting pentagons. [80] A lower score indicates more cognitive impairment. The highest (best) score is 30. The MMSE will be administered at screening only as part of the participant inclusion criteria.

9.1.4 Montreal Cognitive Assessment (MoCA)

The MoCA is a brief mental status exam which was designed to be more sensitive to MCI and early dementia than the MMSE. [81] It assesses numerous cognitive domains, including attention and concentration, executive functions, memory, language, visuoconstructional skills, conceptual thinking, calculations, and orientation. Like the MMSE, the highest (best) score is 30.

9.1.5 ADAS-Cog-Exec

The ADAS-Cog-Exec is a cognitive composite outcome that was designed to improve the performance characteristics of the ADAS-Cog for MCI and early AD clinical trials and to enhance measurement sensitivity to detect changes in executive functioning [82]. It is an optimally-weighted composite of scores on ADAS-Cog13 Word Recall, Delayed Word Recall, Orientation, and Number Cancellation subtests; Trail-Making A & B, Digit Symbol Substitution and Category Fluency; and cognitive components of the CDR (Memory, Orientation, Judgement & Problem Solving). Modeling based on data from ADNI-1 demonstrated that the ADAS-Cog-Exec is superior to the ADAS-Cog13 in detecting cognitive change over 12 months in participants with MCI, resulting in improved statistical power.

9.2 Behavioral and Functional Measures

9.2.1 Clinical Dementia Rating (CDR) Scale – Sum of Boxes (SOB)

The CDR-SB [83] is a validated composite rating of cognition and everyday functioning used in longitudinal AD research which incorporates both informant input and direct assessment of performance. It assesses through semi structured interview 3 cognitive domains including memory, orientation, and judgement/problem solving and 3 everyday functional domains including community affairs, home and hobbies and personal care. There are 5 levels of impairment from none CDR=0 to severe CDR=3. The individual domain scores are added to create a sum of the box scores.

9.2.2 The Alzheimer's Disease Cooperative Study – Activities of Daily Living Scale for use in Mild Cognitive Impairment (ADCS-ADL-MCI)

The ADCS-ADL-MCI is an adapted version of the ADCS-ADL [84] that is intended for use in patients with MCI and mild dementia [85]. It is a structured questionnaire completed with the informant to assess the participant's ability to perform basic and instrumental activities of daily living. Activities assessed include dressing; social and occupational functioning; household chores and use of tools; interest in and ability to carry out hobbies; shopping and meal preparation; managing appointments; using a phone and computer/tablet.

9.2.3 Neuropsychiatric Inventory (NPI)

The NPI is a well-validated, reliable, multi-item instrument to assess psychiatric symptoms in AD dementia based on the results of a structured interview with the study partner. [86] The NPI evaluates both the frequency and severity of 10 neuropsychiatric features, including delusions, hallucinations, agitation/aggression, dysphoria, anxiety, euphoria, apathy, disinhibition, irritability and lability, and aberrant motor behavior, as well as evaluates sleep and appetite/eating disorders. Frequency assessments range from 1 (occasionally, less than once per week) to 4 (very frequently, once or more per day or continuously). Severity assessments range from 1 (mild) to 3 (severe). The score for each subscale is the product of severity and frequency and the total score is the sum of all subscales.

9.2.4 Modified Hachinski Ischemic Scale (MHIS)

This brief questionnaire, conducted by a clinician, incorporates information regarding medical history, cognitive symptoms and features of stroke, reported by a study partner as well as the neurological examination, and neuroimaging studies. [87]

9.2.5 Columbia-Suicide Severity Rating Scale (C-SSRS)

Consistent with FDA guidance [88], the C-SSRS will be implemented throughout the study. The C-SSRS captures the occurrence, severity, and frequency of suicide-related thoughts and behaviors during the corresponding assessment period. [89] The scale includes suggested questions to elicit the type of information needed to determine if a suicide-related thought or behavior occurred. The first time the scale is administered in this study, the C-SSRS "Screening/Baseline" version will be used, and the findings will constitute the baseline assessment. The C-SSRS "Since Last Visit" scale will be used for all subsequent assessments. If the investigator determines that a participant is at risk of suicide or self-harm, appropriate measures to ensure the participant's safety and to obtain mental health evaluation must be implemented. The event should be recorded as either an AE or SAE as determined by the investigator and reported within 24 hours to the Sponsor.

9.2.6 CAGE-AID Substance Abuse Screening Tool

The CAGE Adapted to Include Drugs (CAGE-AID) questionnaire is a valid screening instrument for alcohol and abuse of other drugs. It includes 4 brief "yes" or "no" questions that have been

adapted to include both alcohol and other drug abuse. The CAGE has demonstrated high test-retest reliability and adequate correlations with other screening instruments. [90]

9.3 Research Satisfaction Survey

A Research Satisfaction Survey will be administered to the participant and study partner to evaluate satisfaction with the study. The survey may reveal specific aspects of the study that participants dislike which can inform efforts to improve their experiences when participating in future studies. Past studies show that participant input and feedback is important for retention. [91]

9.4 Treatment Blinding Questionnaire

Following the week 72 clinic assessment (or early termination visit if participant completes study before week 72), the site Investigator and Raters will each complete a Treatment Blinding Questionnaire to document knowledge of intervention group assignment per participant.

10 EARLY DISCONTINUATION/WITHDRAWAL PROCEDURES

The entire study may be discontinued at the discretion of ADCS or the project directors. In these circumstances the Investigator will arrange for all ongoing participants to be seen and for their IP to be discontinued as soon as is safely possible.

Participants are free to withdraw from study participation at any time, for any reason, and without prejudice.

Discontinuation of study treatment and/or the participation of an individual patient in the study will be terminated in the following circumstances:

1. Withdrawal of informed consent by the participant or LAR. If the study partner withdraws his/her consent to participate then attempts will be made to find a replacement. In any event the patient will be continued in the study in so far as possible. Participants who withdraw consent will be advised by the Investigator regarding subsequent treatment and investigation.

2. Treatment of a participant with a non-permitted concomitant medication may necessitate discontinuation from IP and will be determined by the Investigator in conjunction with the ADCS Medical Monitor.

3. Adverse event or other significant medical condition which, in the opinion of the Investigator render it necessary to discontinue IP.

4. The participant experiences a medical emergency that necessitates unblinding their treatment assignment. (see Section 6.6)

5. Any other occurrence that, in the Investigator's opinion, makes continued participation contrary to the participant's best interests.

6. Movement of participant into a long-term care nursing facility. Movement into an assisted living facility is not cause for discontinuation from the study.

Participants who discontinue study treatment for any reason will have the opportunity to continue on the protocol with further visits per protocol to the end of the study with their ongoing consent. Their continued participation will be encouraged.

The Investigators at each site will make every reasonable effort to maximize participant retention, even if the study treatment is discontinued before week 72. However, if an investigator removes a participant from study, or if a participant declines all further study participation during the study, an Early Termination Visit will be completed as close as possible to the time of study discontinuation. The Early Termination Visit may contain the same assessments as week 72, to allow collection of the main outcome measures. The Post-Treatment Safety Telephone Follow-Up visit (4 weeks after the early termination/end of treatment) should also be conducted. For further detail please refer to the Study Procedures Manual.

11 DEFINITION OF ADVERSE EVENTS

An adverse event (AE) or adverse experience is any untoward medical occurrence in a study participant who is administered a medicinal product, that does not necessarily have a causal relationship with the study treatment, and that occurs after informed consent is signed and up to 30 days after the IP has been discontinued. An AE can therefore be any unfavorable and unintended sign, symptom, or disease temporally associated with the use of a medicinal product, whether or not considered related to the medicinal product. Pre-existing conditions, which increase in frequency or severity or worsen in nature during, or as a consequence of, use of a drug in human clinical trials, will also be considered adverse experiences. Adverse events may also include pre- or post-treatment complications that occur as a result of protocol-mandated procedures (e.g., invasive procedures such as a lumbar puncture). Adverse events that occur prior to first dose of IP will be documented as medical history.

An AE **does not** include:

- Medical or surgical procedures (e.g., surgery, endoscopy, tooth extraction, transfusion). The condition that leads to the procedure is the AE.
- Situations where an untoward medical occurrence has not occurred (e.g., hospitalization for pre-planned elective surgery (planned prior to baseline) in which the underlying condition did not worsen during study participation, social and/or convenience admissions).
- Overdose of either IP or concomitant medication without any signs or symptoms unless the participant is hospitalized for observation. Overdoses should be reported as outlined in Section 11.4.

A treatment emergent AE (TEAE) is defined as any AE that developed, worsened, or became serious after first dose of IP and prior to 30 days after the last dose of IP.

11.1 Evaluation and Reporting of Adverse Events

All AEs (i.e. a new event or an exacerbation of a pre-existing condition) that occur from the time of consent and up to 30 days after the IP has been discontinued must be recorded as an AE on the AE eCRF within the EDC. Adverse events that occur prior to initiation of IP will be documented as medical history. The Investigator must follow all AEs until the AE resolves, or until the Investigator and/or the Medical Monitor determine the event is chronic or clinically stable. If an AE remains unresolved at the conclusion of the study, the Investigator and Medical Monitor will make a clinical assessment to determine whether continued follow-up of the AE is warranted. All participants who have received at least one exposure to study therapy will be evaluated for safety of study treatment.

The Investigator should attempt to establish a diagnosis of the event based on signs, symptoms and/or other clinical information. In such cases, the diagnosis should be documented as the AE and not the individual signs/symptoms.

11.2 Assessment of Adverse Events

All AEs must be promptly documented on the Adverse Event eCRF and assessed by the Investigator. Details of the event must include the dates of onset and resolution, severity, relationship to IP, seriousness, and whether the event caused the participant to withdraw from the study, outcome and timing with regard to administration of the IP.

Severity: Severity should be graded and recorded according to the table below.

Severity	Definition
Mild	Awareness of event but easily tolerated
Moderate	Discomfort enough to cause interference with usual activity
Severe	Inability to carry out usual activity, incapacitating, requires medical intervention

Relationship: The relationship of the Adverse Event to the IP will be determined by the Principal Investigator, and assessed using the following definitions:

Relatedness	Description
Not Related	There is no evidence of a causal relationship and a causal relationship cannot be reasonably attributed to the study treatment or procedures. The event is clearly due to extraneous causes.
Unlikely Related	A poor temporal relationship exists between the event onset and administration of intervention. The event could easily be explained by the participant's clinical state, intercurrent illness, or concomitant therapies.

Possibly Related	A relationship cannot be ruled out with certainty and the event may be related. There is some evidence to suggest a causal relationship, but the influence of other factors may have contributed to the event, such as the participant's clinical condition or concomitant treatment.
Probably Related	The event is likely related to the intervention. There is evidence to suggest a causal relationship, such as reasonable temporal sequence from treatment administration or procedure. The influence of other factors is unlikely.
Definitely Related	The event is clearly related to the intervention. There is clear evidence to suggest a causal relationship. The influence of other factors can be ruled out.

These criteria, in addition to good clinical judgment, should be used as a guide for determining the causal assessment. If it is felt that the event is not related to IP therapy, then an alternative explanation should be provided.

11.3 Collection and Reporting of Serious Adverse Events

Following written consent to participate in the study, all SAEs, whether related or not related to IP, must be collected, including those thought to be associated with protocol-specific procedures. The reporting period ends 30 days after discontinuation of dosing. In addition, the investigator should report any SAE occurring after this time period that is believed to be related to IP or protocol-specific procedures. Serious adverse events that occur prior to initiation of IP will be documented in the EDC and reported to ADCS on an SAE form as described below.

An SAE is an AE from this study that results in any of the following outcomes:

- Death
- Life-threatening situation (participant is at immediate risk of death)
- Inpatient hospitalization or prolongation of existing hospitalization
- Persistent or significant disability/incapacity
- Congenital anomaly/birth defect in the offspring of a patient who received IP
- Considered significant by the investigator for any other reason

An SAE report should be completed for any event where doubt exists regarding its status of seriousness.

If the investigator believes that an SAE is not related to the IP but is potentially related to the conditions of the study (such as a withdrawal of previous therapy or a complication related to study procedure), the relationship should be specified in the narrative section of the SAE Report Form.

The Site Investigator, or designated staff, is responsible for reporting all SAEs and all Other Important Medical Events, whether related or not related to IP, overdose, and potential drug induced liver injury, to the ADCS, immediately or no later than 24 hours after the Investigator

becoming aware of the event. For this study we will be capturing SAE information through the study EDC system (TrialMaster) (i.e., event term, start stop dates, causality, and severity) according to study-specific eCRF Completion Guidelines. The ADCS Medical Monitor and ADCS Project Director will be notified via email upon entry of an SAE into the EDC system. The DSMB may at any time request additional information from the ADCS in relation to a reported event.

In addition to reporting to the ADCS via the EDC system, all applicable SAEs must be reported by the Site Investigator to the central IRB (Advarra) promptly, but no later than 10 business days of the site PI becoming aware of the event, according to Advarra's reporting requirements for such events (https://www.advarra.com/reporting-to-the-irb-serious-adverse-events-saes-in-drug-studies/). SAEs that are determined to be **possibly, probably, or definitely related to IP** must be reported **within 24 hours** to the central IRB.

Finally, SAEs should be reported to local site IRBs, as applicable, according to local IRB reporting requirements.

If only limited information is initially available, follow-up reports are required. If an ongoing SAE changes in its intensity or relationship to IP or if new information becomes available, a follow-up SAE report should be sent using the same procedure used for the transmission of the initial SAE and the same event term should be used. In addition, new or revised event information must be entered into the EDC at the same time.

All SAEs should be followed to resolution or stabilization. For any questions relating to SAEs, please contact the ADCS Medical Monitor via telephone or email at the number listed on the protocol face page.

11.3.1 Suspected Unexpected Serious Adverse Reactions

Suspected unexpected serious adverse reactions (SUSARs) are SAEs that are not listed in the Investigator's Brochure as an expected AE and that the Investigator assesses as possibly related or probably related or definitely related to IP or study procedure. United States 21 CFR 312.32 requires the reporting of SUSARs to regulatory authorities within 7 calendar days for fatal SUSARs and 15 calendar days for non-fatal SUSARs. The IND holder is responsible for, and has procedures consistent with, regulations that will be followed for expedited reporting of SUSARs.

11.4 Overdose Reporting Requirements

For the purposes of this study, an overdose is the accidental or intentional administration of any dose of IP greater than the highest daily dose within a calendar day. Any overdose must be reported on the eCRF. An overdose is not an AE unless it results in untoward effects. Any AEs related to the overdose must be reported on the AE eCRF. Signs and symptoms of overdose should be treated according to standard of care.

11.5 Clinical Laboratory Abnormalities and Other Abnormal Assessments

Laboratory abnormalities are usually not recorded as AEs unless considered to be clinically significant by the site clinician. An abnormal laboratory result will be considered an AE if it is associated with clinical signs or symptoms, if the abnormality is of a degree that requires active management (e.g. discontinuation of the IP, dose modification) or when the event is requiring treatment or other therapeutic intervention (e.g. iron supplements, blood transfusion, etc.).

The Investigator will evaluate the relationship of any significantly abnormal result to protocol treatment and clinical condition, if possible. All clinically significant abnormal laboratory results will be followed until they return to normal or become stabilized.

11.6 Pregnancy and Breast Feeding

Benfotiamine should not be used during pregnancy or while breast-feeding. The lower age limit for this study is 50 years, and women must be post-menopausal for at least 1 year or surgically sterile (bilateral tubal ligation, hysterectomy or bilateral oophorectomy) for at least 6 months prior to screening to be eligible for this study.

Male participants with partners of child bearing potential must agree to effective contraception or abstinence for the duration of the study.

12 STATISTICAL METHODS

This is a randomized double-blind placebo-controlled phase 2 A/B trial of 18 months of treatment with benfotiamine in participants with early AD. Phase 2A of the trial will select the highest well-tolerated dose from among 1200 or 600 mg/day of benfotiamine, followed by a test of the efficacy of approximately 18 months of treatment with the selected dose of benfotiamine in phase 2B. Throughout the trial, comprehensive safety monitoring will be provided quarterly by the DSMB. The study is designed with dual primary endpoints, including a cognitive and a functional measure, with strict type I error control over the primary and secondary endpoints

12.1 Analysis Populations and sensitivity analyses

Analysis populations are defined as follows:

- Enrolled Participants: Participants who signed an informed consent form and were assigned a Participant Identification number (PID).
- Randomized Participants (ITT Population): Enrolled participants who received a treatment assignment.
- Treated Participants (Safety Population): Enrolled and randomized participants who received at least 1 dose of blinded study therapy. This will be the primary analysis population used for safety analyses.

- The modified Intent-to-Treat (mITT) population will include all randomized participants who took at least one dose of the study medication, who have a baseline assessment of the co-primary efficacy endpoints, and who have at least one efficacy evaluation following baseline. This will be the primary analysis population used for the efficacy analyses.

The primary population for all efficacy analyses is the mITT population, without imputation for missing values. Planned sensitivity analyses will use the mITT population with multiple imputation for missing values. The Treated Participants will be used for analyses of safety endpoints. An exploratory dose response analysis will use serum thiamine levels as the received dose indicator, in the mITT population, for the co-primary endpoints only.

12.2 Phase 2A

12.2.1 Overview

Phase 2A of the trial will randomize approximately 150 participants total 1:1:1 to treatment with 1200 mg/day benfotiamine, 600 mg/day benfotiamine or placebo. The randomization stratification factor will be site. At the end of phase 2A, the highest safe and well-tolerated dose of benfotiamine will be recommended to be carried forward to the phase 2B efficacy studies.

The Phase 2A safety and dose selection analysis will be conducted when 160 person-months of exposure have accumulated in each of the high dose arm and the placebo arm, or alternatively when 21 total TEs have been observed across both the high dose arm and the placebo arm, whichever is earlier. At the expected time of the dose selection analysis, approximately 30 participants will have been on treatment for 12 weeks or more according to our enrollment projections.

12.2.1 Phase 2A safety and tolerability analysis.

The phase 2A primary outcome will be the rate of tolerability events (TEs). A TE will be defined as either discontinuation of treatment related to intolerability or a post-randomization moderate or severe AE that is possibly, probably or definitely related to IP.

Phase 2A secondary outcomes will include all adverse events, physical exam, vital signs, health status, 12-lead ECG, laboratory determinations and participant withdrawals. There will be no efficacy outcomes in phase 2A.

12.2.1.1 Primary analysis

The phase 2A primary analysis will compare the rate of tolerability events (TEs) between the 1200 mg day arm and the placebo arm. The analysis will test whether the rate of TEs is unacceptably high in this high-dose arm compared to placebo, using a formal one-sided hypothesis test of equal rates, against the one-sided alternative that the high dose arm TE rate is greater than the placebo arm TE rate. The conditional one-sided test of Przyborowski and Wilenski (1940) [92] will be used, at 5% significance level. Briefly, the test statistic will be the proportion of TEs that occur in the active arm, out of the total number of accumulated TEs in

both arms; under the null hypothesis of equal event rates, this test statistic has an approximately binomial distribution with probability ½. The test will be conducted when 160 person-months of exposure have accumulated in each arm, or alternatively when 21 total TEs have been observed across both the high dose arm and the placebo arm, whichever is earlier.

From accrual patterns and preliminary data from a recently completed RCT evaluating the safety and efficacy of edonerpic in 300 participants with mild to moderate AD [93], under the null hypothesis of equal tolerability at the event rate seen in the placebo arm of that trial (0.66 events/person/month), is expected to occur at about calendar month 9 of the trial. The test is expected to have 80% power to detect an elevated TE rate in the active arm which is greater than the placebo arm by a factor of 3.2, which is the rate ratio observed in the edonerpic trial comparing the highest dose of edonerpic to placebo [93]. The highest dose of edonerpic was judged to be very safe and well tolerated, thus a factor of 3.2 is expected to provide an acceptable safety and tolerability margin. Statistical details and further justification of this threshold are given below.

Type I error rate: The hypothesis test will have 5% probability to declare that the high dose has an unacceptable safety and tolerability profile, if the TE rate is actually the same between the high dose and placebo arms.

Power to drop the high dose in case of unacceptable tolerability: The hypothesis test will declare the high dose arm to be unacceptable with 80% probability if the rate of TEs in the high dose arm is more than 3.2 times that of the placebo arm.

If the high dose arm is found to have a significantly elevated rate of TEs compared to the placebo arm at the 5% significance level, then the lower dose arm will be taken forward and tested against placebo, for the duration of the study. This step-down procedure will control the Type I error rate across the two comparisons.

12.2.1.2 Dose decision

The unblinded statistician will inform study leadership that criteria for performing the phase 2A analysis has been reached. At this time, data will be locked and the unblinded safety statistician will prepare a report giving the details of the analysis and providing a full unblinded safety report. The final recommendation on dose decision will be made by the DSMB.

12.2.1.3 Additional Phase 2A safety analyses

An exact upper-one sided 95% confidence interval for the TE rate in each arm will be computed. Safety and tolerability data as well as demographic data will be summarized in tabular and/or graphical format for each treatment group. The incidence of all laboratory test abnormalities and the median changes from baseline will be tabulated by treatment regimen and time point. The safety population will be used for these analyses.

12.3 Transition from Phase 2A to Phase 2B

To inform the bounds of an acceptable adverse event rate, we compared data from the high dose arm to the placebo to the recently completed double-blind randomized trial, which tested 18

months' treatment with 300 participants with mild to moderate AD [93]. Over the first 8 months of the trial, with 20 participants and about 100 person-months of exposure in each arm, the TE rate in the high dose arm was found to be about 0.21 events per subject per month, a factor of 3.2 times higher than the TE rate in the placebo (0.067 events per subject per month). Most of these events were moderate AEs and none were serious, with a few dropouts, providing an example of a well-tolerated therapeutic agent. Thus, we will take the highest acceptable rate of TEs in the high dose benfotiamine arm to be 3.2 times higher than the placebo arm rate, reasoning that this should provide an ample safety and tolerability margin.

Table 1 shows the number of events needed for the conditional binomial test to have 80% power to detect a given rate ratio, for two assumed event rates in the placebo arm. Note that the number of events needed depends on the rate ratio, but not the baseline rate of TEs in the placebo arm. To detect an elevated rate ratio of 3.2 times or higher we will need to see about 21 total events before we conduct the test. Note that the number of person months needed to accumulate the desired number of events is less at higher event rates. Thus, we will lock the data and conduct the primary analysis when either 21 events have occurred, or when 160 person-months of exposure have occurred in each of the high dose and placebo arms, whichever is sooner. This assures that, in case the event rate is higher than expected, the analysis will be conducted as soon as it is adequately powered. However, if event rates are lower than expected, so that by 160 person-months of exposure in each arm fewer than 21 total events have occurred, no safety concerns are expected. Thus, it will be appropriate to conduct the test even with fewer than 21 events, and to send a full safety and tolerability report forward for DSMB review. From our accrual models, we expect to accumulate the needed exposure time by about calendar month 9 of the trial or earlier.

Table 1. Number of events needed for adequate power, and expected timing of the analysis.

				person-months needed, per arm	
rate ratio active/ placebo	active arm rate	placebo arm rate	total # events needed	alternative hypothesis	null hypothesis
2.8	0.186	0.066	25	98	189
**3.2**	**0.207**	**0.066**	**21**	**76**	**158**
3.5	0.227	0.066	18	61	137
2.8	0.279	0.100	26	67	128
**3.2**	**0.321**	**0.100**	**21**	**48**	**102**
3.5	0.352	0.100	18	39	89

12.4 Phase 2B

12.4.1 Overview

The highest tolerated dose of benfotiamine will be carried forward from phase 2A. Phase 2B is a double-blind placebo-controlled trial of benfotiamine vs placebo in early AD over a treatment period of 18 months. The dual co-primary endpoints are within-subject change in CDR-SB and ADAS-Cog13, chosen because of their good sensitivity to show change over 2 years in similar study populations [94]. Type I error is controlled by a gatekeeper strategy. The final accrued

sample size is 406 participants (n=203 active, n=203 placebo). No more than 25% drop out is anticipated, and a drop-in rate of 5% is assumed (i.e., 5% of placebo arm participants are assumed to use benfotiamine dietary supplement).

At the start of phase 2B, all participants enrolled in the two phase 2A active dose arms continue with the selected phase 2B dose. All phase 2A patients will be included in the phase 2 ITT efficacy population, as assigned to active or placebo treatment. Newly enrolled patients will be randomized 1:2 active arm to placebo arm until parity is achieved between numbers of active and placebo arm participants (approximately 150 participants total), and randomized 1:1 thereafter (approximately 100 participants).

The two co-primary efficacy endpoints are the CDR-SB and the ADAS-Cog13, tested jointly using a gatekeeper strategy at familywise 5% significance level. Only if both endpoints are significant will the primary endpoint of Phase 2B have been met. In this gatekeeper strategy, each endpoint will be tested at 5% significance without adjustment of alpha, however in hierarchical order. If the CDR-SB is significant then the ADAS-Cog13 will be tested. If the CDR-SB is not significant, results on the ADAS-Cog13 will not be tested and will be presented as exploratory only. This strategy allows the overall alpha for the joint hypothesis to be controlled, while using a 5% significance level for each endpoint. The rationale for prioritizing the CDR-SB is the guidance from the Food and Drug Administration (FDA) suggesting that this measure be the primary outcome in clinical trials of early AD [95].

12.4.2 Sample Size, Power and Effect Sizes

At the end of phase 2A, all participants on active treatment will move to the selected dose for the remainder of the trial, and will be included in the final efficacy mITT population for phase 2B. It is anticipated that approximately 50 of 406 total participants in the active arm will have received a median of 4 months of exposure (out of 18) at a different dose, given our accrual models, before moving to the selected dose for the remainder of the 18- month study period. Given the extensive safety record of benfotiamine, it is further anticipated that the 1200 mg dose will be selected. Thus, this cohort of patients exposed at an anticipated lower cumulative dose is not expected to cast doubt on the efficacy of 1200 mg benfotiamine in case of a successful phase 2 trial at this dose, given that the efficacy of benfotiamine is expected to only increase at higher doses. We also note that preliminary data support the efficacy of the lower 600 mg dose of benfotiamine; thus power for phase 2B is expected to be adequate, as further demonstrated below.

As seen in Table 2, with a total sample size of n=406, 20% dropout and 5% drop-in from the placebo arm to active treatment, the trial will be powered at 80% overall, 90% on each endpoint separately, to detect a difference between arms corresponding to a Cohen's D effect size of 0.38, which is a small to moderate effect size in statistical terms. As shown below, this effect size corresponds to reasonable and clinically relevant difference between arms on each co-primary endpoint, corresponding to a 35% to 40% amelioration of the decline seen in the placebo arm in the recently completed phase 2 trial of donanemab in patients with early AD [96], and corresponding to an absolute difference between arms on the CDR-SB of 0.7 points (assumed

SD = 1.74), and on the ADAS-Cog13 of 1.7 points (assumed SD = 4.35). Thus, it was decided that 406 participants is a reasonable sample size for the trial.

Table 2. Sample sizes which achieve 90% power for given effect sizes (Cohen's D; difference between the 2 arms), assuming 20% dropout and assuming drop-in rates of 5% and 10%.

drop-in rate	effect size	n / arm
5%	0.35	239
**5%**	**0.38**	**203**
5%	0.40	183
10%	0.35	266
10%	0.38	226
10%	0.40	204

The sample size is computed in order to have 80% power to detect a significant effect on both primary endpoints. Thus, the power for each endpoint will be set to 90%. If the joint alternative is true (if both the CDR-SB and the ADAS-Cog13 are impacted in a beneficial direction) then overall power will be at least 0.90 x 0.90 = 0.81 (as these two tests are positively correlated), attaining the desired 80% power for the joint endpoint.

The recently completed donanemab trial of 18 months' treatment in participants with early AD was used as a reference to provide preliminary data on detectable effect sizes for the joint alternative hypothesis in the co-primary endpoints [96]. With 406 participants, we are powered to detect a mean difference between which is about 41% of the observed change in the donanemab trial [96] placebo group on the CDR-SB and about 35% of the observed change on the ADAS- Cog13. In absolute terms, this would be a difference between arms of about 0.7 points on the CDR-SB and about 1.7 points on the ADAS-Cog13, which are a modest but clinically significant differences. Details supporting these numbers are given below.

These power calculations for phase 2B are based on a somewhat conservative approximation using a two-sided Student's two-sample t-test at 5% alpha. The actual primary analysis will use a mixed-effects repeated measures model, described further below. A drop-out rate of 20 percent at 18 months is assumed, considering the low dropout rate of 11% over 1 year observed in the pilot study of benfotiamine [7] , the similar 20 percent dropout rate over 2 years that was seen in the placebo group in a 24 month trial of a multinutrient (LipiDiDiet) in participants with prodromal AD [97], the higher 25% dropout over 1 year seen in the high dose arm of the recently completed edonerpic RCT [93] and the dropout rate of 27% seen in the recently completed donanemab study of 18 months' treatment in early AD [96]. A drop-in rate of 5 percent is projected based on the preliminary data from the pilot study (i.e., 5% of placebo arm participants are assumed to use out of study benfotiamine dietary supplement). To adjust for these factors, sample sizes were inflated by 25% to accommodate the dropout, and effect sizes attenuated by 5% to accommodate the drop in, as recommended [98].

12.4.2.1 Effect size for CDR-SB

To translate this Cohen's D to a clinically interpretable difference for the CDR-SB, we reviewed the literature for data on observed changes in similar populations. Considering recent trials of 18 months' duration, the standardized mean change in the placebo arm of the Donanemab study is estimated to be 0.92 (Fig 2b: estimated mean change 1.6, estimated SD of change (0.2 *sqrt(76)) = 1.74). This seems reasonable, as it compares to effect sizes for 24 months of an unexpectedly low 0.65 in the LipiDiDiet study placebo arm [97](mean change= 1.12, SD =1.72) and a somewhat larger 1.03 (mean change = 2.39, SD of 2.32) over 2 years for the early AD group enrolled in ADNI [99]

Thus, taking the donanemab study as reference [96], this study is powered to detect a mean difference between arms which is about 41% of the observed change in the donanemab placebo group (0.38/0.92 = 0.41). In absolute terms, this would be a difference between arms of about 0.7 points on the CDR-SB (0.38 * 1.74 = 0.7), which is a modest but clinically significant difference.

12.4.2.2 Effect sizes for the ADAS-Cog13

In the event of a significant result on the primary CDR-SB assessment, the next measure to be tested will be the ADAS-Cog13. In the Donanemab study, standardized mean change of the ADAS-Cog13 in the placebo arm at the 18-month endpoint is estimated to be 1.10 (estimated mean change 4.8, estimated SD of change (0.5 *sqrt(76)) = 4.35. Thus, for the ADAS-Cog13 we are powered to detect a mean difference between arms which is about 35% of the observed change in the placebo group (0.38/1.10 = 0.35). In absolute terms, this would be a difference between arms of about 1.7 points on the ADAS-Cog13, which is again a modest but clinically significant difference.

12.4.3 Analysis Plan

12.4.3.1 Co-Primary Endpoints

The primary study hypothesis is that treatment with benfotiamine will result in a reduction in participant change on both the total CDR-SB score and the ADAS-Cog13, relative to the placebo group at week 72 in the mITT population.

The primary analysis will be the same for each co-primary endpoint. It will test the within subject change in the measure as the outcome in a mixed effects repeated measures model, using all available outcomes in the mITT population, with no imputation for missing data. Fixed effects in the model will be APOE status, baseline value of the outcome measure, baseline x visit-week interaction term, treatment group, visit-week, and visit-week x treatment group interaction. The approach will be maximum likelihood. Visit is treated as a categorical variable.

The covariance structure will be specified as follows: the random effects will include site and subject. The within-subject covariance will be unstructured. If needed, sites will be pooled (in order of enrollment, starting from minimum enrollment) so that there is a minimum of 5 participants per site. If the model fails to converge at the default setting for the software used, then site will be changed to a fixed effect, and if this model does not converge site will be

removed from the model. If the model does not converge, the following structures for the within-subject covariance will be fit, sequentially, until the structure is found that results in convergence of the model: Huynh-Feldt, Toeplitz, Autoregressive (1), and Compound Symmetry. A sandwich estimator will be utilized to estimate the variance estimates and degrees of freedom will be calculated using the between-within method.

The primary endpoint will be tested using model-adjusted least squares means at the week 72 visit. Point estimates, standard errors, two-sided 95% confidence intervals, and p-values will be presented.

12.4.3.2 **Secondary Endpoints**

Secondary endpoints are:

- Serum Thiamine and its esters, ThDP and ThMP
- ADCS-ADL-MCI
- MoCA

Mean changes in the secondary endpoints from baseline to week 72 will be compared between arms using the analysis strategy outlined above. To control alpha over the primary and all secondary endpoints, an overall hierarchical gatekeeper strategy will be used, similar to the primary analysis. First, if the primary analysis is not significant, then the secondary endpoints will not be formally tested, and will be presented using exploratory summary statistics only (means and uncorrected 95% confidence intervals by arm and for the difference between arms). No claims of statistical significance will be made. However, if the primary analysis is significant, the secondary endpoints will be tested in the listed order in hierarchical manner, each using an alpha of 5%. Once a secondary endpoint has failed to attain the prespecified 5% significance level, the remaining secondary endpoints will be presented as exploratory only. This strategy will maintain the overall familywise significance level for the primary and all secondary endpoints at 5%.

Descriptive statistics will also be presented. Categorical variables will be summarized by treatment arm using frequency distributions: the number and percentage of non-missing observations will be given.

Continuous variables will be summarized using standard quantitative statistics: the number of non-missing observations, mean, standard deviation, median and range (minimum and maximum observed values).

12.4.3.3 **Exploratory Endpoints**

Exploratory endpoints are:

- Additional neurocognitive measures (NTB-Remote, NTB-In-Person)
- Additional composite scores (ADAS-Cog Exec)
- Neuropsychiatric symptoms (NPI)
- Regional cortical thickness and volumetric changes measured by cranial MRI
- Pharmacodynamic endpoints: Advanced glycation end products (AGE)

- Downstream biological markers including plasma Neurofilament Light (NfL), Glial Fibrillary Acid Protein (GFAP), Plasma total tau (t-tau)
- AD biological markers including plasma p-tau 231, plasma Abeta 42/40

Exploratory analyses will be detailed in the statistical analysis plan, and will generally be similar to the primary and secondary analyses.

Additional exploratory analyses. Sensitivity analyses will be conducted on the primary and secondary endpoints using the per-protocol population. In addition, a dose response analysis will be conducted for each of the co-primary endpoints, using the mITT population and serum thiamine levels as the received dose indicator. There will be no subgroup analyses.

12.5 Safety Analysis

Safety endpoints include AEs, physical exam abnormalities, vital signs, 12-lead ECG, safety laboratory determinations and participant withdrawals. Safety and tolerability data as well as demographic data will be summarized in tabular and/or graphical format for each treatment group. The incidence of all safety laboratory test abnormalities and the median changes from baseline will be tabulated by treatment regimen and time point. The safety population will be used for these analyses.

12.5.1 Adverse Events

AEs occurring after the start of IP dosing at baseline (week 0) will be summarized descriptively for the safety population. All AEs will be coded according to system organ class (SOC) and preferred term (PT) using a Medical Dictionary for Regulatory Activities (MedDRA) dictionary. Summary tables showing the number of participants and percent within each category will be generated for each of the following types of AEs and its relationship to study treatment (related to study treatment):

- All events
- Serious events
- Deaths
- Events leading to withdrawal
- Severe events

12.5.2 Laboratory Parameters

Laboratory parameters will be summarized by visit. Frequencies of high and low values with respect to the normal range will be displayed, as will shift tables comparing each treatment visit and baseline visit by time point and treatment group.

12.5.3 Other Safety Parameters

Vital signs and ECG parameters will be summarized across groups by visit using descriptive statistics, and at each outcome visit and at end of study.

Physical examination findings and number of participants will be summarized as the count and percentage of participants by eCRF pre-defined categories at last visit. Concomitant medications will be summarized by treatment group, drug class and PT. Vital signs will be summarized by visit using descriptive statistics.

Overall interpretation results for ECGs and the Investigator interpretation results are collected as normal, abnormal not clinically significant, and abnormal clinically significant. Participants whose interpretation shifts from normal to abnormal will be listed separately including description of the abnormality and any associated comments.

12.6 **Protocol Deviations, Data Blind Review, and Unblinding**

Protocol deviations will not be prospectively granted by the sponsor. If deviations occur, such as a visit or sampling window being missed, the investigator must decide whether to complete the visit or sample collection outside of the protocol-defined window or not. The medical monitor must be notified immediately when protocol deviations are discovered so that a decision about whether to retain the participant in the study can be made. Classification of deviations from the protocol as important or not important and decisions regarding exclusion of participants will be decided on a case-by-case basis without knowledge of the treatment assigned and before the database lock (Data Blind Review).

Only when an emergency occurs that requires a departure from the protocol for an individual participant will there be such a departure without the sponsor’s pre-approval. The nature and reasons for the protocol deviation will be recorded in the participant’s eCRF, and the Site Principal Investigator must notify the Medical Monitor. Protocol deviations will be reported in the final study report.

13 RECORDING AND COLLECTION OF DATA

13.1 **Case Report Form**

The Site Principal Investigator or designee will record all data collected on the eCRF provided for that purpose. The site will be suitably trained on the use of the eCRF and appropriate site personnel will be provided electronic signature privileges.

All site entries will be made in a secured web site and the Site Principal Investigator will review the record for completeness. Upon completion of the review, the site PI will sign electronically in the signature page of the eCRF.

The Site Investigator or designee will make necessary eCRF corrections. This investigator must authorize the corrections to the entered data on eCRF.

Completed eCRFs will be submitted according to the ADCS's eCRF Completion Guidelines and reviewed by the ADCS to determine their acceptability. If necessary, data correction requests will be generated for resolution by the study site.

13.2 Study Files and Patient Source Documents

Participant confidentiality is strictly held in trust by the participating investigators, research staff, and the sponsoring institution and their agents. This confidentiality is extended to cover testing of biological samples and genetic tests in addition to the clinical information relating to participants. Authorized representatives of the sponsoring institution may inspect all documents and records required to be maintained by the Investigator, including but not limited to, medical records (office, clinic or hospital) and pharmacy records for the participants in this study. The clinical study site will permit access to such records. Any data, specimens, forms, reports, video recordings, and other records that leave the site will be identified only by a participant identification number to maintain confidentiality.

The Investigator must maintain adequate and accurate records to enable the conduct of the study to be fully documented and the study data to be subsequently verified. These documents include Investigators' Study Files, original participant clinical source documents generated at the study site or completed source document worksheets provided by ADCS. The term "original" means the first recording of the data.

The Investigator will ensure the site master files are maintained, including the study protocol and its amendments, IRB and regulatory approvals with associated correspondence, informed consents, IP records, staff curriculum vitae, all correspondence, and other appropriate documents.

Participant clinical source documents may include, but are not limited to, participant hospital/clinic records, physicians' and nurses' notes, appointment books, laboratory reports, ECGs, magnetic resonance imaging (MRI) images, pathology and special assessment reports. The Investigator must assure that all original source documents are available to support monitoring activities. The Investigator must also ensure that redacted source documents are uploaded to the EDC at the time of eCRF data entry.

13.3 Rater Training

Site staff will be trained on the assessments and study specific cognitive and behavioral measures as described in Section 9. The sites will also have training at the Investigator meeting. Scales will be collected on paper and entered via the EDC. Details will be provided with the Cognitive Assessments Manual for Administration and Scoring and in the Study Procedures Manual.

Each site will have at least two rater personnel for BenfoTeam: one cognitive rater to administer the cognitive scales, and one CDR rater, who will be blinded to cognitive results, to administer the CDR.

13.4 Monitoring

During the study each site will be monitored at regular intervals by an ADCS clinical research monitor, through a combination of on-site visits and remote monitoring procedures. The monitoring visits must be conducted according to the applicable ICH and GCP guidelines to ensure protocol adherence, quality of data, drug accountability, compliance with

regulatory requirements and continued adequacy of the investigational site and its facilities. The Investigator will co-operate in the monitoring process by ensuring the availability of the eCRFs, source documents (uploaded to the EDC) and other necessary documents at the time of monitoring activities and by prompt attention to any matters brought to his/her attention by the monitor.

13.5 Audit

ICH guidelines for GCP require independent inspection of clinical program activities. Such inspections may be performed at any time before, during and/or after the study. The Investigator and study staff are responsible for maintaining the Investigator Site File containing all study-related regulatory documentation as outlined by ADCS Regulatory Affairs that will be suitable for inspection at any time by ADCS, its designees, and/or regulatory agencies. The Investigator understands and agrees to give access to the necessary documentation and files.

13.6 Retention of Data

All records connected with this clinical study will be retained for at least two years following the date of an approved marketing application [21 CFR 312.62(c)] for the IP for the indication for which it is being investigated; or if no application is to be filed or if the application is not approved for such indication, until 2 years after the investigation is discontinued and FDA is notified; or for at least 3 years following study termination, whichever is longer. Prior to record disposal, ADCS may elect to extend the retention period. To ensure that these standards are applied, written permission must be granted from ADCS before record disposal. All local laws regarding retention of records must also be followed. Study sites are required to retain all records until written notification allowing destruction is received from the ADCS.

13.7 Reporting of Study Results

The Project Directors, in cooperation with ADCS and the ADCS Principal Investigator, will provide annual reports and a final report on the trial to the National Institutes of Health as a responsibility of holding its grant funding. Dr. Gibson or designated representatives at Burke Neurological Institute and Weill Cornell Medicine will produce an integrated Clinical Study Report for the development of benfotiamine and the regulatory responsibility as the IND holder.

13.8 Quality Assurance/Quality Control

ADCS Standard Operating Procedures (SOPs) will be adhered to for all activities relevant to the quality of the study. Documentation of all quality control procedures will be outlined in the Data Management Plan (DMP). Edit checks and listings will be run and used in conjunction with the eCRF pages to support a clinical review of the data. Documentation of all quality control procedures will be outlined in the DMP.

14 DATA SAFETY MONITORING BOARD

The DSMB, which is independent from the ADCS, and which has reporting to the NIA through its project scientist, will provide safety oversight of the trial. It has a standing membership of a chair, and 4 other experts in the field, who have the appropriate background for these responsibilities and have been approved by the NIA. Other members may be added depending on the needs of the trial.

The DSMB will meet quarterly as well as ad-hoc in the face of any important safety matters arising. The DSMB will also be informed of SUSARs as they are being reported to regulatory authorities and Investigators. In this trial, they will provide recommendations following each of their meetings. These may include continuing the study as designed, amending safety monitoring procedures, modifying the protocol or the ICF, or recommending the termination of the study. There will be a study-specific appendix to the DSMB charter developed for this trial detailing additional DSMB roles and responsibilities for this protocol.

14.1 Additional Phase 2A Safety Monitoring

A formal phase 2A dose decision analysis is described throughout this protocol (see Sections 6.4 and 12.1.1.1). During phase 2A, the DSMB will review the status of this dose decision analysis at each quarterly meeting, which will be included in the regular DSMB report. At the time of the phase 2A analysis, data will be locked and the unblinded safety statistician will prepare a report giving the details of the analysis and providing a full unblinded safety report. The final recommendation on dose decision will be made by the DSMB.

Additional details of their responsibilities and review are detailed in the DSMB Charter.

14.2 Additional Phase 2B Safety Monitoring

DSMB will continue to meet regularly, at quarterly meetings during phase 2B to review safety and concerns arising. Additional details of their responsibilities and review are detailed in the DSMB Charter.

15 PUBLICATIONS POLICY AND SHARING OF DATA

ADCS, in collaboration with the Project Directors, will publish the study results in accordance with the 2010 Consolidated Standards of Reporting Trials (CONSORT) guidelines [100]. See Appendix II for the CONSORT Checklist and Flowchart.

As there are expected to be too few participants studied at each site for individual site's results to be statistically valid, the results of this study will be disclosed or published only in combined form based upon the statistical analysis performed by ADCS and will be coordinated by ADCS. No disclosure of study results will be permitted except as specified in a separate, written agreement between ADCS and the Investigator. This study will be registered at www.ClinicalTrials.gov after approval of the designated IRB and prior to enrollment of the first participant, as required for publication by the International Committee of Medical Journal Editors (ICMJE).

Results will be posted on clinicaltrials.gov in accordance with requirements.

References

1. *2017 Alzheimer's disease facts and figures.* Alzheimer's Association; The Journal of the Alzheimer's Association, 2017.
2. Pan, X., et al., *Powerful beneficial effects of benfotiamine on cognitive impairment and beta-amyloid deposition in amyloid precursor protein/presenilin-1 transgenic mice.* Brain, 2010. **133**(Pt 5): p. 1342-51.
3. Tapias, V., et al., *Benfotiamine treatment activates the Nrf2/ARE pathway and is neuroprotective in a transgenic mouse model of tauopathy.* Hum Mol Genet, 2018. **27**(16): p. 2874-2892.
4. Anandam, K.Y., et al., *Proinflammatory cytokines inhibit thiamin uptake by human and mouse pancreatic acinar cells: involvement of transcriptional mechanism(s).* Am J Physiol Gastrointest Liver Physiol, 2021. **320**(1): p. G108-G116.
5. He, Y., et al., *Glyoxalase system: A systematic review of its biological activity, related-diseases, screening methods and small molecule regulators.* Biomed Pharmacother, 2020. **131**: p. 110663.
6. Yang, Y., et al., *Succinylation Links Metabolic Reductions to Amyloid and Tau Pathology*. 2019, bioRxiv.
7. Gibson, G.E., et al., *Benfotiamine and Cognitive Decline in Alzheimer's Disease: Results of a Randomized Placebo-Controlled Phase IIa Clinical Trial.* J Alzheimers Dis, 2020. **78**(3): p. 989-1010.
8. Fargo, K.N., et al., *2014 Report on the Milestones for the US National Plan to Address Alzheimer's Disease.* Alzheimers Dement, 2014. **10**(5 Suppl): p. S430-52.
9. Greenwood, J., E. Love, and O. Pratt, *Kinetics of thiamine transport across the blood-brain barrier in the rat.* The Journal of Physiology, 1982. **327**(1): p. 95-103.
10. Reggiani, C., C. Patrini, and G. Rindi, *Nervous tissue thiamine metabolism in vivo. I. Transport of thiamine and thiamine monophosphate from plasma to different brain regions of the rat.* Brain research, 1984. **293**(2): p. 319-327.
11. Gibson, G.E., et al., *Reduced activities of thiamine-dependent enzymes in the brains and peripheral tissues of patients with Alzheimer's disease.* Arch Neurol, 1988. **45**(8): p. 836-40.
12. Gold, M., R.A. Hauser, and M.F. Chen, *Plasma thiamine deficiency associated with Alzheimer's disease but not Parkinson's disease.* Metab Brain Dis, 1998. **13**(1): p. 43-53.
13. Bubber, P., et al., *Mitochondrial abnormalities in Alzheimer brain: mechanistic implications.* Ann Neurol, 2005. **57**(5): p. 695-703.
14. Gibson, G.E., et al., *Mitochondrial damage in Alzheimer's disease varies with apolipoprotein E genotype.* Annals of neurology, 2000. **48**(3): p. 297-303.
15. Butterworth, R.F. and A.-M. Besnard, *Thiamine-dependent enzyme changes in temporal cortex of patients with Alzheimer's disease.* Metabolic Brain Disease, 1990. **5**(4): p. 179-184.
16. Morris, J.C., *The Clinical Dementia Rating (CDR): current version and scoring rules.* Neurology, 1993. **43**(11): p. 2412-4.
17. Mastrogiacomo, F., C. Bergeron, and S.J. Kish, *Brain alpha-ketoglutarate dehydrogenase complex activity in Alzheimer's disease.* J Neurochem, 1993. **61**(6): p. 2007-14.
18. Gejl, M., et al., *Blood-Brain Glucose Transfer in Alzheimer's disease: Effect of GLP-1 Analog Treatment.* Sci Rep, 2017. **7**(1): p. 17490.
19. Gejl, M., et al., *In Alzheimer's Disease, 6-Month Treatment with GLP-1 Analog Prevents Decline of Brain Glucose Metabolism: Randomized, Placebo-Controlled, Double-Blind Clinical Trial.* Front Aging Neurosci, 2016. **8**: p. 108.
20. Xie, F., et al., *Pharmacokinetic study of benfotiamine and the bioavailability assessment compared to thiamine hydrochloride.* J Clin Pharmacol, 2014. **54**(6): p. 688-95.

21. Hilbig, R. and H. Rahmann, *Comparative autoradiographic investigations on the tissue distribution of benfotiamine versus thiamine in mice.* Arzneimittelforschung, 1998. **48**(5): p. 461-8.
22. Sheng, L., et al., *Safety, Tolerability and Pharmacokinetics of Single and Multiple Ascending Doses of Benfotiamine in Healthy Subjects.* Drug Des Devel Ther, 2021. **15**: p. 1101-1110.
23. Blass, J.P. and G.E. Gibson, *Abnormality of a thiamine-requiring enzyme in patients with Wernicke-Korsakoff syndrome.* N Engl J Med, 1977. **297**(25): p. 1367-70.
24. Gallant, J., et al., *Low-dose thiamine supplementation of lactating Cambodian mothers improves human milk thiamine concentrations: a randomized controlled trial.* Am J Clin Nutr, 2021.
25. Jones, K.S., et al., *Erythrocyte transketolase activity coefficient (ETKAC) assay protocol for the assessment of thiamine status.* Ann N Y Acad Sci, 2020.
26. Whitfield, K.C., et al., *Thiamine deficiency disorders: diagnosis, prevalence, and a roadmap for global control programs.* Ann N Y Acad Sci, 2018. **1430**(1): p. 3-43.
27. Gomes, F., et al., *Thiamine deficiency unrelated to alcohol consumption in high-income countries: a literature review.* Ann N Y Acad Sci, 2021.
28. Hammes, H.P., et al., *Benfotiamine blocks three major pathways of hyperglycemic damage and prevents experimental diabetic retinopathy.* Nat Med, 2003. **9**(3): p. 294-9.
29. Stracke, H., et al., *Efficacy of benfotiamine versus thiamine on function and glycation products of peripheral nerves in diabetic rats.* Exp Clin Endocrinol Diabetes, 2001. **109**(6): p. 330-6.
30. Acosta, D.M., D. Eliezer, and G.E. Gibson, *Post Translational Modifications by Succinylation and Acetylation*, in *Reference Module in Life Sciences*. 2020, Elsevier.
31. Jack, C.R., et al., *NIA-AA Research Framework: Toward a biological definition of Alzheimer's disease.* Alzheimers Dement, 2018. **14**(4): p. 535-562.
32. Calingasan, N.Y., et al., *Accumulation of amyloid precursor protein-like immunoreactivity in rat brain in response to thiamine deficiency.* Brain Res, 1995. **677**(1): p. 50-60.
33. Karuppagounder, S.S., et al., *Thiamine deficiency induces oxidative stress and exacerbates the plaque pathology in Alzheimer's mouse model.* Neurobiol Aging, 2009. **30**(10): p. 1587-600.
34. Dumont, M., et al., *Mitochondrial dihydrolipoyl succinyltransferase deficiency accelerates amyloid pathology and memory deficit in a transgenic mouse model of amyloid deposition.* Free Radic Biol Med, 2009. **47**(7): p. 1019-27.
35. Cullen, K.M. and G.M. Halliday, *Neurofibrillary tangles in chronic alcoholics.* Neuropathol Appl Neurobiol, 1995. **21**(4): p. 312-8.
36. Cullen, K.M., et al., *The nucleus basalis (Ch4) in the alcoholic Wernicke-Korsakoff syndrome: reduced cell number in both amnesic and non-amnesic patients.* J Neurol Neurosurg Psychiatry, 1997. **63**(3): p. 315-20.
37. de Moraes, R.C.M., et al., *Oral benfotiamine reverts cognitive deficit and increase thiamine diphosphate levels in the brain of a rat model of neurodegeneration.* Exp Gerontol, 2020. **141**: p. 111097.
38. Derk, J., et al., *The Receptor for Advanced Glycation Endproducts (RAGE) and Mediation of Inflammatory Neurodegeneration.* J Alzheimers Dis Parkinsonism, 2018. **8**(1).
39. Chou, P.S., et al., *Effect of Advanced Glycation End Products on the Progression of Alzheimer's Disease.* J Alzheimers Dis, 2019. **72**(1): p. 191-197.
40. Prasad, K., *AGE-RAGE stress: a changing landscape in pathology and treatment of Alzheimer's disease.* Mol Cell Biochem, 2019. **459**(1-2): p. 95-112.
41. Haddad, M., et al., *Circulating and Extracellular Vesicles Levels of N-(1-Carboxymethyl)-L-Lysine (CML) Differentiate Early to Moderate Alzheimer's Disease.* J Alzheimers Dis, 2019. **69**(3): p. 751-762.

42. Wang, G.J., et al., *Intersubject variability of brain glucose metabolic measurements in young normal males.* J Nucl Med, 1994. **35**(9): p. 1457-66.
43. Bär, K.J., et al., *Pentosidine and N(epsilon)-(carboxymethyl)-lysine in Alzheimer's disease and vascular dementia.* Neurobiol Aging, 2003. **24**(2): p. 333-8.
44. Sharma, A., et al., *Advanced glycation end products and protein carbonyl levels in plasma reveal sex-specific differences in Parkinson's and Alzheimer's disease.* Redox Biol, 2020. **34**: p. 101546.
45. Ataç, Z.S., et al., *G82S polymorphism of receptor for advanced glycation end products gene and serum soluble RAGE levels in mild cognitive impairment and dementia of Alzheimer's type patients in Turkish population.* J Clin Neurosci, 2019. **59**: p. 197-201.
46. Spauwen, P.J., et al., *Associations of advanced glycation end-products with cognitive functions in individuals with and without type 2 diabetes: the maastricht study.* J Clin Endocrinol Metab, 2015. **100**(3): p. 951-60.
47. Wang, C., et al., *High thiamine diphosphate level as a protective factor for Alzheimer's disease.* Neurol Res, 2018. **40**(8): p. 658-665.
48. Lambert, J.C., et al., *Meta-analysis of 74,046 individuals identifies 11 new susceptibility loci for Alzheimer's disease.* Nat Genet, 2013. **45**(12): p. 1452-8.
49. Wolters, F.J., et al., *The impact of APOE genotype on survival: Results of 38,537 participants from six population-based cohorts (E2-CHARGE).* PLoS One, 2019. **14**(7): p. e0219668.
50. Deo, P., et al., *APOE ε4 Carriers Have a Greater Propensity to Glycation and sRAGE Which Is Further Influenced by RAGE G82S Polymorphism.* J Gerontol A Biol Sci Med Sci, 2020. **75**(10): p. 1899-1905.
51. Mooldijk, S.S., et al., *Letter to the Editor, Reacting to: "APOE ε4 Carriers Have a Greater Propensity to Glycation and sRAGE Which Is Further Influenced by RAGE G82S Polymorphism".* J Gerontol A Biol Sci Med Sci, 2020. **75**(10): p. 1906-1907.
52. Li, Y.M. and D.W. Dickson, *Enhanced binding of advanced glycation endproducts (AGE) by the ApoE4 isoform links the mechanism of plaque deposition in Alzheimer's disease.* Neurosci Lett, 1997. **226**(3): p. 155-8.
53. Alkhalaf, A., et al., *Effect of benfotiamine on advanced glycation endproducts and markers of endothelial dysfunction and inflammation in diabetic nephropathy.* PLoS One, 2012. **7**(7): p. e40427.
54. Depeint, F., et al., *Marginal thiamine deficiency increases oxidative markers in the plasma and selected tissues in F344 rats.* Nutrition Research, 2007. **27**(11): p. 698-704.
55. Pekovich, S.R., P.R. Martin, and C.K. Singleton, *Thiamine deficiency decreases steady-state transketolase and pyruvate dehydrogenase but not alpha-ketoglutarate dehydrogenase mRNA levels in three human cell types.* J Nutr, 1998. **128**(4): p. 683-7.
56. Calingasan, N.Y., et al., *Oxidative stress is associated with region-specific neuronal death during thiamine deficiency.* J Neuropathol Exp Neurol, 1999. **58**(9): p. 946-58.
57. Hazell, A.S., et al., *Selective down-regulation of the astrocyte glutamate transporters GLT-1 and GLAST within the medial thalamus in experimental Wernicke's encephalopathy.* J Neurochem, 2001. **78**(3): p. 560-8.
58. Manzetti, S., J. Zhang, and D. van der Spoel, *Thiamin function, metabolism, uptake, and transport.* Biochemistry, 2014. **53**(5): p. 821-35.
59. Calingasan, N.Y., et al., *Novel neuritic clusters with accumulations of amyloid precursor protein and amyloid precursor-like protein 2 immunoreactivity in brain regions damaged by thiamine deficiency.* Am J Pathol, 1996. **149**(3): p. 1063-71.
60. Peña, C.E. and R. Felter, *Ultrastructural changes of the lateral vestibular nucleus in acute experimental thiamine deficiency.* Z Neurol, 1973. **204**(4): p. 263-80.

61. Tellez, I. and R.D. Terry, *Fine structure of the early changes in the vestibular nuclei of the thiamine-deficient rat.* Am J Pathol, 1968. **52**(4): p. 777-94.
62. Haupt, E., H. Ledermann, and W. Kopcke, *Therapeutics-Benfotiamine in the treatment of diabetic polyneuropathy--A three-week randomized, controlled pilot study (BEDIP Study).* International Journal of Clinical Pharmacology and Therapeutics, 2005. **43**(2): p. 71-77.
63. Stracke, H., et al., *Benfotiamine in diabetic polyneuropathy (BENDIP): results of a randomised, double blind, placebo-controlled clinical study.* Exp Clin Endocrinol Diabetes, 2008. **116**(10): p. 600-5.
64. Available from: http://benfotiamine.org/www.benfotiamine.org/index.html.
65. Stirban, A., A. Pop, and D. Tschoepe, *A randomized, double-blind, crossover, placebo-controlled trial of 6 weeks benfotiamine treatment on postprandial vascular function and variables of autonomic nerve function in Type 2 diabetes.* Diabet Med, 2013. **30**(10): p. 1204-8.
66. Contreras, C.L., et al. *Advanced glycation end products (AGEs) and sRAGE levels after benfotiamine treatment in diabetes mellitus type 2*. Special Issue: Experimental Biology 2017 Meeting Abstracts 2018. **31**, DOI: 10.1096/fasebj.31.1_supplement.646.32.
67. Alkhalaf, A., et al., *A double-blind, randomized, placebo-controlled clinical trial on benfotiamine treatment in patients with diabetic nephropathy.* Diabetes Care, 2010. **33**(7): p. 1598-1601.
68. Kirmess, K.M., et al., *The PrecivityAD™ test: Accurate and reliable LC-MS/MS assays for quantifying plasma amyloid beta 40 and 42 and apolipoprotein E proteotype for the assessment of brain amyloidosis.* Clinica Chimica Acta, 2021. **519**: p. 267-275.
69. Hu, Y., et al., *Assessment of a plasma amyloid probability score to estimate amyloid positron emission tomography findings among adults with cognitive impairment.* JAMA network open, 2022. **5**(4): p. e228392-e228392.
70. Fogelman, I., et al., *Independent study demonstrates amyloid probability score accurately indicates amyloid pathology.* Annals of Clinical and Translational Neurology, 2023.
71. Rissman, R.A., et al., *Plasma Aβ42/Aβ40 and phospho-tau217 ratios increase the accuracy of amyloid PET classification in preclinical Alzheimer's Disease.* Alzheimer’s & Dementia [accepted and in press], 2023.
72. Rey, A., *L’examen Clinique en psychologie.* Paris: Presses Universitaires de France, 1964.
73. Besser, L., et al., *Version 3 of the National Alzheimer's Coordinating Center's Uniform Data Set.* Alzheimer Dis Assoc Disord, 2018.
74. Morris, J.C., et al., *The Consortium to Establish a Registry for Alzheimer's Disease (CERAD). Part I. Clinical and neuropsychological assessment of Alzheimer's disease.* Neurology, 1989. **39**(9): p. 1159-65.
75. Kaplan, E., H. Goodglass, and S. Weintraub, *The Boston Naming Test.* Philadelphia: Lea and Febiger, 1983.
76. Office, A.G.s., *Manual of Directions and Scoring. In: Army Individual Test Battery.* Washington DC: War Department, 1944.
77. Wechsler, D., *Wechsler Adult Intelligence Scale-Revised. ; 1981.* The Psychological Corporation, 1981.
78. Rosen, W.G., R.C. Mohs, and K.L. Davis, *A new rating scale for Alzheimer's disease.* Am J Psychiatry, 1984. **141**(11): p. 1356-64.
79. Mohs, R.C., et al., *Development of cognitive instruments for use in clinical trials of antidementia drugs: additions to the Alzheimer's Disease Assessment Scale that broaden its scope.* Alzheimer disease and associated disorders, 1997.
80. Folstein, M.F., S.E. Folstein, and P.R. McHugh, *"Mini-mental state". A practical method for grading the cognitive state of patients for the clinician.* J Psychiatr Res, 1975. **12**(3): p. 189-98.

81. Nasreddine, Z.S., et al., *The Montreal Cognitive Assessment, MoCA: a brief screening tool for mild cognitive impairment.* J Am Geriatr Soc, 2005. **53**(4): p. 695-9.
82. Jacobs, D.M., et al., *The ADAS-Cog-Exec: A Novel Cognitive Composite Outcome to Assess Therapeutic Effects of Exercise in the EXERT Trial for Adults with MCI.* J Prev Alzheimers Dis, 2019. **6**: p. S117-118.
83. Hughes, C.P., et al., *A new clinical scale for the staging of dementia.* Br J Psychiatry, 1982. **140**: p. 566-72.
84. Teng, E., et al., *Utility of the functional activities questionnaire for distinguishing mild cognitive impairment from very mild Alzheimer disease.* Alzheimer Dis Assoc Disord, 2010. **24**(4): p. 348-53.
85. Galasko, D., et al., *ADCS Prevention Instrument Project: assessment of instrumental activities of daily living for community-dwelling elderly individuals in dementia prevention clinical trials.* Alzheimer Disease & Associated Disorders, 2006. **20**: p. S152-S169.
86. Cummings, J.L., *The Neuropsychiatric Inventory: assessing psychopathology in dementia patients.* Neurology, 1997. **48**(5 Suppl 6): p. S10-16.
87. Rosen, W.G., et al., *Pathological verification of ischemic score in differentiation of dementias.* Ann Neurol, 1980. **7**(5): p. 486-8.
88. Administration, U.S.F.a.D., *Guidance for Industry: Suicidality: Prospective Assessment of Occurrence in Clinical Trials, Draft Guidance* 2012.
89. Posner, K., et al., *The Columbia–Suicide Severity Rating Scale: initial validity and internal consistency findings from three multisite studies with adolescents and adults.* American journal of psychiatry, 2011. **168**(12): p. 1266-1277.
90. Ewing, J.A., *Detecting alcoholism: the CAGE questionnaire.* Jama, 1984. **252**(14): p. 1905-1907.
91. Miller, S.D., et al., *The partners for change outcome management system.* J Clin Psychol, 2005. **61**(2): p. 199-208.
92. Przyborowski, J. and H. Wilenski, *Homogeneity of results in testing samples from Poisson series: With an application to testing clover seed for dodder.* Biometrika, 1940. **31**(3-4): p. 313-323.
93. Schneider, L.S., et al., *Safety and Efficacy of Edonerpic Maleate for Patients With Mild to Moderate Alzheimer Disease: A Phase 2 Randomized Clinical Trial.* JAMA Neurol, 2019.
94. Raghavan, N., et al., *The ADAS-Cog revisited: novel composite scales based on ADAS-Cog to improve efficiency in MCI and early AD trials.* Alzheimers Dement, 2013. **9**(1 Suppl): p. S21-31.
95. Schneider, L.S., *Rethinking the Food and Drug Administration's 2013 guidance on developing drugs for early-stage Alzheimer's disease.* Alzheimers Dement, 2014. **10**(2): p. 247-50.
96. Mintun, M.A., et al., *Donanemab in Early Alzheimer's Disease.* N Engl J Med, 2021. **384**(18): p. 1691-1704.
97. Soininen, H., et al., *24-month intervention with a specific multinutrient in people with prodromal Alzheimer's disease (LipiDiDiet): a randomised, double-blind, controlled trial.* Lancet Neurol, 2017. **16**(12): p. 965-975.
98. Wittes, J., *Sample size calculations for randomized controlled trials.* Epidemiol Rev, 2002. **24**(1): p. 39-53.
99. Cedarbaum, J.M., et al., *Rationale for use of the Clinical Dementia Rating Sum of Boxes as a primary outcome measure for Alzheimer's disease clinical trials.* Alzheimers Dement, 2013. **9**(1 Suppl): p. S45-55.
100. Schulz, K.F., et al., *CONSORT 2010 Statement: Updated guidelines for reporting parallel group randomised trials.* J Clin Epidemiol, 2010. **63**(8): p. 834-40.

101. Albert, M.S., et al., *The diagnosis of mild cognitive impairment due to Alzheimer's disease: recommendations from the National Institute on Aging-Alzheimer's Association workgroups on diagnostic guidelines for Alzheimer's disease.* Alzheimers Dement, 2011. **7**(3): p. 270-9.
102. McKhann, G.M., et al., *The diagnosis of dementia due to Alzheimer’s disease: Recommendations from the National Institute on Aging-Alzheimer’s Association workgroups on diagnostic guidelines for Alzheimer's disease.* Alzheimer's & Dementia, 2011. **7**(3): p. 263-269.

16 APPENDICES

16.1 APPENDIX I: STUDY PLAN AND PROCEDURES AT EACH VISIT

**Visit Number**	1	2	3	4	5	6	7	8	9	10 EOT/Early Term	11 Post Tx Safety FU
**Study Visit Time Point**	Screening (-42 d)	Baseline (Week 0)	Wk 4 (±7 d)	Wk 8 (±7 d)	Wk 12 (±7 d)	Wk 24 (±7 d)	Wk 36 (±7 d)	Wk 48 (±7 d)	Wk 60 (±7 d)	Wk 72 (±7 d)	Wk 76 (±7 d)
Informed Consent	X										
Eligibility Review	X	X									
Randomization[1]		X									
Med History/Demographics	X										
Modified Hachinski Ischemic Scale	X										
Weight & Height[2]	X	X	X	X	X	X	X	X	X	X	
Physical Examination	X	X	X	X	X	X	X	X	X	X	
Neurological Examination	X										
Vital Signs[3]	X	X	X	X	X	X	X	X	X	X	
Concomitant Medication	X	X	X	X	X	X	X	X	X	X	X
Adverse Events[4]		X	X	X	X	X	X	X	X	X	X
12-lead ECG (resting)	X					X				X	
Safety Blood Tests[5]	X	X	X	X	X	X	X	X	X	X	
TSH	X										
Hemoglobin A1c	X										
Urinalysis[6]	X										
Infectious Disease Serology[7]	X										
Blood Sampling for PK/PD[8]		X		X						X	
Blood Sampling for PrecivityAD2 test (Aβ42/Aβ40 ratio, p-tau217/np-tau217(p-tau217 Ratio))	X									X	
Blood Sampling for Biomarkers (total tau, p-tau-231, NfL, GFAP)		X								X	
Blood Collection for Biobanking		X						X		X	
Cranial MRI[9]	X									X	

**Visit Number**	1	2	3	4	5	6	7	8	9	10 EOT/Early Term	11 Post Tx Safety FU
**Study Visit Time Point**	Screening (-42 d)	Baseline (Week 0)	Wk 4 (±7 d)	Wk 8 (±7 d)	Wk 12 (±7 d)	Wk 24 (±7 d)	Wk 36 (±7 d)	Wk 48 (±7 d)	Wk 60 (±7 d)	Wk 72 (±7 d)	Wk 76 (±7 d)
Optional Lumbar Puncture (LP) for CSF10 biomarkers[10]		X								X	
Post-LP Safety Telephone[11]		X								X	
CAGE-AID	X										
Columbia-Suicidality Severity Rating Scale (C-SSRS)	X	X	X	X	X	X	X	X	X	X	
MoCA	X					X		X		X	
MMSE	X										
CDR	X					X		X		X	
ADAS-Cog-13		X				X		X		X	
ADCS-ADL-MCI		X				X		X		X	
NTB Remote[12]		X				X		X		X	
NTB In-Person[13]		X				X		X		X	
NPI		X				X		X		X	
Research Satisfaction Survey		X				X		X		X	
Dispense IP[14]		X	X	X	X	X	X	X	X		
IP Accountability			X	X	X	X	X	X	X	X	
Treatment Blinding Questionnaire[15]										X	
Telephone Follow-Up											X

1 Randomization must occur at the baseline visit after eligibility is confirmed.

2 Height is done at screening only.

3 Vital signs include systolic and diastolic blood pressure, pulse, temperature, and respiration rate.

4 The reporting period for all AEs and SAEs starts at the screening visit (i.e. when the patient or LAR signs consent). The end of the reporting period for both SAEs and AEs is 30 days after the IP has been discontinued.

5 Clinical Safety Laboratory Tests will be performed by a Central Laboratory. Assessment includes the following: Hematology (hemoglobin, hematocrit, platelets, RBC, WBC, differential count, and absolute neutrophil count), Chemistry (sodium, potassium, magnesium, chloride, calcium, ALT, AST, LDH, alkaline phosphatase, GGT, phosphorus, bicarbonate, CPK, total protein, albumin, indirect bilirubin, direct bilirubin, total bilirubin, glucose, creatinine, BUN,

uric acid , total cholesterol, LDL, HDL, triglycerides, eGFR), B12 (screening only), folate (screening only).

6 Urinalysis to include: color, appearance, pH, specific gravity, protein, glucose, ketones, urobilinogen, bilirubin, blood, leucocytes, esterase, nitrite, crystals, casts, debris.

7 Infectious disease serology includes the following: Human Immunodeficiency Virus (HIV), and Rapid Plasma Reagin (RPR, syphilis).

8 A blood sample to measure thiamine level and its esters thiamine diphosphate (ThDP), thiamine monophosphate (ThMP), and ThDP activationof transketolase (PK) should be collected at baseline, week 8, and week 72 for all participants. A blood sample to measure AGE (PD) should also be collected at baseline, week 8, and week 72 for all participants. At baseline and week 8, blood will be collected once just prior to the morning dose and again approximately 2 hours (+/- 30 minutes) after their routine morning dose. At week 72, blood will be collected at a single timepoint (steady-state sample).

9 All MRIs must be performed per imaging protocol and must use the same scanner throughout study.

a. Screening MRI: if a patient has not had an MRI performed within 6 months of screening (i.e. within 6 months from the date of informed consent), then an MRI must be performed as part of the screening requirements for this study, per the imaging protocol, and should be one of the last screening procedures performed to determine final eligibility in order to prevent patients from undergoing unnecessary MRIs. If a patient has had an MRI within 6 months of screening (i.e. within 6 months from the date of informed consent) but the MRI does not follow the study-specific imaging protocol, another MRI must be performed per the imaging protocol, and must occur as close to, and prior to, the baseline visit, after all other eligibility criteria have been confirmed.
b. Week 72 MRI: the protocol window for MRI at week 72 is 14 days before and up to14 days after the week 72 visit time point. If a participant is terminating early at 36 weeks or after, obtain MRI. If MRI is performed on the same day as a lumbar puncture at week 72, the MRI must be conducted before the lumbar puncture. Otherwise, at least a 3-day window between MRI and the lumbar puncture is required.

10 Visit windows for optional lumbar puncture at baseline for CSF are: up to 42 days prior to first dose of IP and within 7 days of the week 72 study visit. If a patient is terminating early, they do not need to undergo lumbar puncture.

11 Post lumbar puncture safety follow up telephone call must occur 1 to 3 days after the lumbar puncture is performed.

12 NTB-Remote assessments will be administered over videoconference-call within 10 calendar days after each in-person visit (baseline, week 24, week 48 and week 72). Participants will use their own interactive video capable devices for the NTB-Remote assessment. An interactive video capable device may be provided to participants who do not have one. Participants will be instructed to use the same device for all remote assessments. Remote assessments include Rey Auditory Verbal Learning Test Immediate and Delayed Recall, Number Span Forward and Backward, Category Fluency, and Letter Fluency.

13 NTB-In Person assessments include Boston Naming Test (30-item), Trail Making Test (Trails A and B) and Digit Symbol Substitution

14 Participant will take the first dose in person during their baseline clinic visit. Participant will be instructed to take the second dose later that same day.

15 Treatment blinding questionnaire to be administered to site PI and Raters.

16.2 APPENDIX II – NIA/AA DIAGNOSTIC GUIDELINES

<u>Mild Cognitive Impairment (MCI) due to AD [101]</u>

Establish clinical and cognitive criteria:

- Cognitive concern reflecting a change in cognition reported by participant or informant or clinician (i.e., historical or observed evidence of decline over time);
- Objective evidence of impairment in one or more cognitive domains, including memory (i.e., formal or bedside testing to establish level of cognitive function in multiple domains);
- Preservation of independence in functional abilities;
- Not demented.

Examine etiology of MCI consistent with AD pathophysiological process:

- Rule out vascular, traumatic, medical causes of cognitive decline, where possible;
- Provide evidence of longitudinal decline in cognition, when feasible;
- Report history consistent with AD genetic factors, where relevant.

<u>Mild Dementia due to AD [102]</u>

Meets core clinical criteria for all-cause dementia. Dementia is diagnosed when there are cognitive or behavioral (neuropsychiatric) symptoms that:

- Interfere with the ability to function at work or at usual activities; and
- Represent a decline from previous levels of functioning and performing; and
- Are not explained by delirium or major psychiatric disorder;
- Cognitive impairment is detected and diagnosed through a combination of (1) history-taking from the patient and a knowledgeable informant and (2) an objective cognitive assessment, either a “bedside” mental status examination or neuropsychological testing. Neuropsychological testing should be performed when the routine history and bedside mental status examination cannot provide a confident diagnosis.
- The cognitive or behavioral impairment involves a minimum of two of the following domains:
    - Impaired ability to acquire and remember new information—symptoms include: repetitive questions or conversations, misplacing personal belongings, forgetting events or appointments, getting lost on a familiar route.

- Impaired reasoning and handling of complex tasks, poor judgment—symptoms include: poor understanding of safety risks, inability to manage finances, poor decision-making ability, inability to plan complex or sequential activities.
- Impaired visuospatial abilities—symptoms include: inability to recognize faces or common objects or to find objects in direct view despite good acuity, inability to operate simple implements, or orient clothing to the body.
- Impaired language functions (speaking, reading, writing)—symptoms include: difficulty thinking of common words while speaking, hesitations; speech, spelling, and writing errors.
- Changes in personality, behavior, or comportment— symptoms include: uncharacteristic mood fluctuations such as agitation, impaired motivation, initiative, apathy, loss of drive, social withdrawal, decreased interest in previous activities, loss of empathy, compulsive or obsessive behaviors, socially unacceptable behaviors.

The differentiation of dementia from MCI rests on the determination of whether or not there is significant interference in the ability to function at work or in usual daily activities. This is inherently a clinical judgment made by a skilled clinician on the basis of the individual circumstances of the patient and the description of daily affairs of the patient obtained from the patient and from a knowledgeable informant.

The differentiation of mild versus moderate AD is determined by CDR and MMSE. Those with scores of CDR global >1 or MMSE <20 will be considered to have moderate disease and will be ineligible. Those with CDR global of 1 and MMSE $\geq$20 will be considered to be mild AD per protocol.

Meets criteria for dementia as described above, and in addition, has the following characteristics:

- Insidious onset. Symptoms have a gradual onset over months to years, not sudden over hours or days;
- Clear-cut history of worsening of cognition by report or observation;
- The initial and most prominent cognitive deficits are evident on history and examination in one of the following categories:
  - **Amnestic presentation:** Amnestic presentation includes impairment in learning and recall of recently learned information. There should also be evidence of cognitive dysfunction in at least one other cognitive domain, as listed under the criteria for dementia provided above.
  - **Nonamnestic presentations:**
    - i. Language presentation: the most prominent deficits are in word-finding, but deficits in other cognitive domains should be present
    - ii. Visuospatial presentation: the most prominent deficits are in spatial cognition, including object agnosia, impaired face recognition, simultanagnosia, and alexia. Deficits in other cognitive domains should be present
- The diagnosis of probable AD dementia ***should not*** be applied when there is evidence of (a) substantial concomitant cerebrovascular disease, defined by a history of a stroke temporally related to the onset or worsening of cognitive impairment; or the presence of multiple or extensive infarcts or severe white matter hyperintensity burden; or (b) core features of Dementia with Lewy bodies other than

dementia itself; or (c) prominent features of behavioral variant frontotemporal dementia; or (d) prominent features of semantic variant primary progressive aphasia or nonfluent/agrammatic variant primary progressive apahsia; or (e) evidence of another concurrent, active neurological disease, or a non-neurological medical comorbidity or use of medication that could have a substantial effect on cognition.

16.3 APPENDIX III – CONSORT CHECKLIST & DIAGRAM (v2010)

Section/Topic	Item No	Checklist item	Reported on page
**Title and abstract**			
	1a	Identification as a randomized trial in the title	______
	1b	Structured summary of trial design, methods, results, and conclusions (for specific guidance see CONSORT for abstracts)	______
**Introduction**			
Background and objectives	2a	Scientific background and explanation of rationale	______
	2b	Specific objectives or hypotheses	______
**Methods**			
Trial design	3a	Description of trial design (such as parallel, factorial) including allocation ratio	______
	3b	Important changes to methods after trial commencement (such as eligibility criteria), with reasons	______
Participants	4a	Eligibility criteria for participants	______
	4b	Settings and locations where the data were collected	______
Interventions	5	The interventions for each group with sufficient details to allow replication, including how and when they were actually administered	______
Outcomes	6a	Completely defined pre-specified primary and secondary outcome measures, including how and when they were assessed	______
	6b	Any changes to trial outcomes after the trial commenced, with reasons	______

Sample size	7a	How sample size was determined	______
	7b	When applicable, explanation of any interim analyses and stopping guidelines	______
Randomization:			
Sequence generation	8a	Method used to generate the random allocation sequence	______
	8b	Type of randomization; details of any restriction (such as blocking and block size)	______
Allocation concealment mechanism	9	Mechanism used to implement the random allocation sequence (such as sequentially numbered containers), describing any steps taken to conceal the sequence until interventions were assigned	______
Implementation	10	Who generated the random allocation sequence, who enrolled participants, and who assigned participants to interventions	______
Blinding	11a	If done, who was blinded after assignment to interventions (for example, participants, care providers, those assessing outcomes) and how	______
	11b	If relevant, description of the similarity of interventions	______
Statistical methods	12a	Statistical methods used to compare groups for primary and secondary outcomes	______
	12b	Methods for additional analyses, such as subgroup analyses and adjusted analyses	______
**Results**			
Participant flow (a diagram is strongly recommended)	13a	For each group, the numbers of participants who were randomly assigned, received intended treatment, and were analyzed for the primary outcome	______
	13b	For each group, losses and exclusions after randomization, together with reasons	______
Recruitment	14a	Dates defining the periods of recruitment and follow-up	______
	14b	Why the trial ended or was stopped	______
baseline data	15	A table showing baseline demographic and clinical characteristics for each group	______

Numbers analyzed	16	For each group, number of participants (denominator) included in each analysis and whether the analysis was by original assigned groups	
Outcomes and estimation	17a	For each primary and secondary outcome, results for each group, and the estimated effect size and its precision (such as 95% confidence interval)	
	17b	For binary outcomes, presentation of both absolute and relative effect sizes is recommended	
Ancillary analyses	18	Results of any other analyses performed, including subgroup analyses and adjusted analyses, distinguishing pre-specified from exploratory	
Harms	19	All important harms or unintended effects in each group (for specific guidance see CONSORT for harms)	
**Discussion**			
Limitations	20	Trial limitations, addressing sources of potential bias, imprecision, and, if relevant, multiplicity of analyses	
Generalizability	21	Generalizability (external validity, applicability) of the trial findings	
Interpretation	22	Interpretation consistent with results, balancing benefits and harms, and considering other relevant evidence	
**Other information**			
Registration	23	Registration number and name of trial registry	
Protocol	24	Where the full trial protocol can be accessed, if available	
Funding	25	Sources of funding and other support (such as supply of drugs), role of funders	

CONSORT Flow Diagram (v2010)

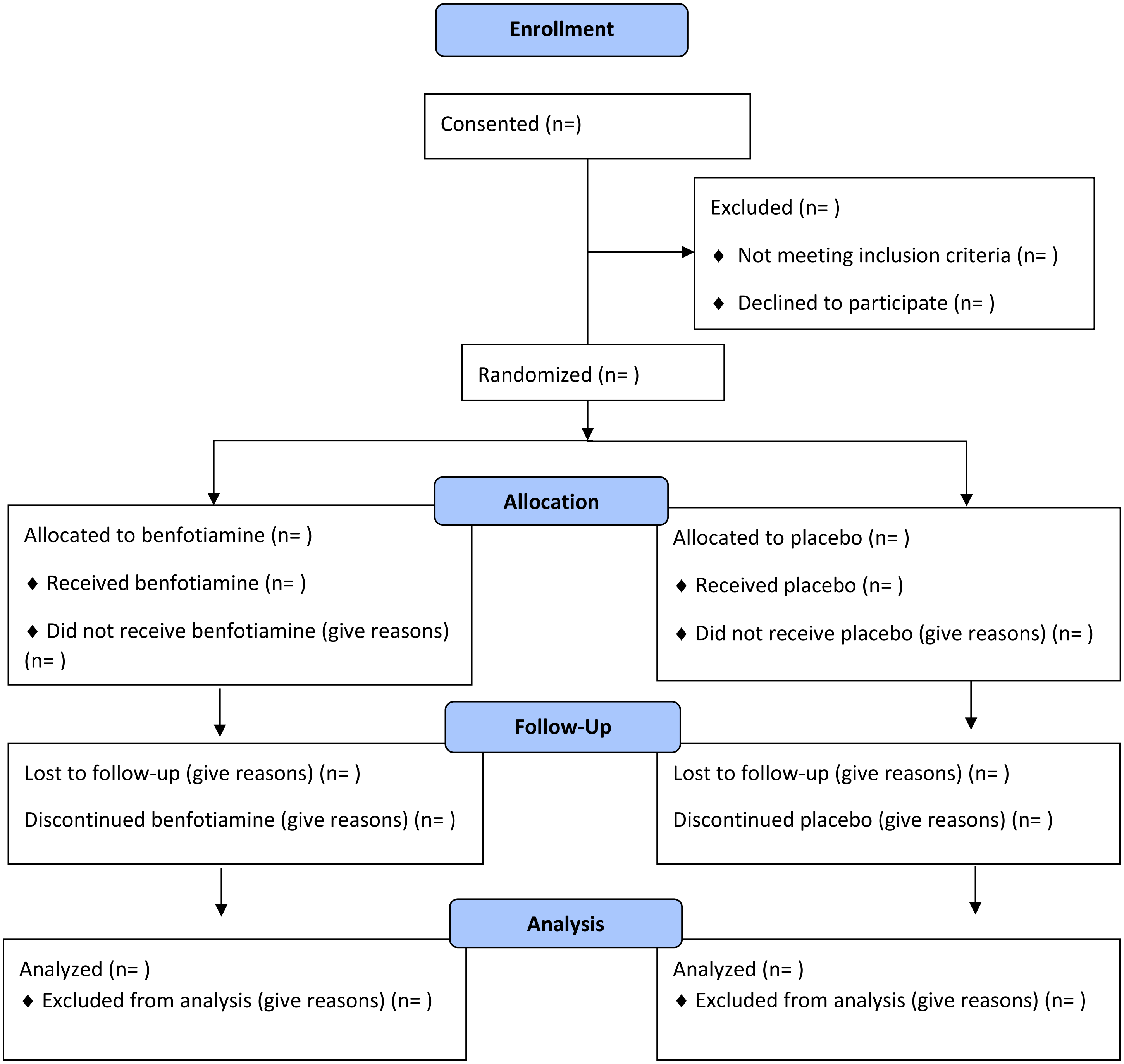

Supplement: S3 File — (PDF) [file pone.0302998.s004.pdf]
