# Supplementary material for: Protocol for a seamless phase 2A-phase 2B randomized double-blind placebo-controlled trial to evaluate the safety and efficacy of benfotiamine in patients with early Alzheimer’s disease (BenfoTeam)
Source: PLoS One. 2024 May 29;19(5):e0302998. doi: 10.1371/journal.pone.0302998 (PMC11135745; doi:10.1371/journal.pone.0302998)
Supplement: S4 File — (PDF) [file pone.0302998.s005.pdf]

INFORMED CONSENT FORM

**Sponsor / Study Title:**	**Winifred Masterson Burke Medical Research Institute / "A Seamless Phase 2A-Phase 2B Randomized Double-Blind Placebo-Controlled Trial to Evaluate the Safety and Efficacy of Benfotiamine in Patients with Early Alzheimer's Disease (BenfoTeam)"**
**Protocol Number:**	**ADC-061-BENFO**
**Principal Investigator: (Study Doctor)**	**«PiFullName»**
**Telephone:**	**«IcfPhoneNumber»**
**Address:**	**«PiLocations»**

INTRODUCTION

You are invited to take part in a research study. The purpose of this research study is to test an investigational drug called Benfotiamine (study drug) as a possible treatment for Alzheimer's disease (AD).

Winifred Masterson Burke Medical Research Institute (Sponsor) (d/b/a Burke Neurological Institute) and The National Institutes of Health (NIH) are sponsoring this research study.

This form is for use in a research study that may involve participants who may or may not have the capacity to consent to take part in the study. When the participant cannot legally consent to take part, pronouns "you" and "your" should be read as referring to the participant rather than the person (legally authorized representative) who is signing this form for the participant. In cases where the participant's representative gives consent, the participant should be informed about the study to the extent possible given his/her understanding. During the course of the study, if the participant regains the capacity to consent, informed consent will be obtained from the participant and the participant offered the ability to leave the study if desired.

KEY INFORMATION ABOUT THIS RESEARCH

This consent form describes a research study and is designed to help you decide if you would like to be a part of it.

This section provides the information we believe is most helpful and important to you in making your decision about participating in this study. Additional information that may help you decide can be found in other sections of the document. The study is meant to define how safe and effective Benfotiamine, a lab-made version of vitamin B1 (thiamine), is in slowing the progression of the symptoms of early Alzheimer's disease in 406 participants during a period of 18 months.

To qualify for this study, there will be questionnaires and medical evaluations performed to see if you are able to participate or not.

Participating in this study may not bring any benefit to you as you may be assigned to take a placebo (inactive substance) instead of Benfotiamine. Neither your study doctor nor you will know what study drug you are receiving.

The research information (data) collected will not identify you and will include the study procedures and collection of how you are doing, including side effects.

This consent describes the purpose, procedures, risks or possible discomforts.

Your participation is entirely voluntary, and you have the right to withdraw at any time from the study.

Please take your time and read this form carefully and discuss any questions you may have with the study doctor or the study staff, as well as family members or friends. If you decide to participate, you must sign your name at the end of this form and date it.

The remaining document will now describe the study in detail.

BACKGROUND AND PURPOSE

You are being asked to participate in this research study because you are an adult with early Alzheimer's disease (AD), supported by the testing that will be done as part of the screening for inclusion.

The purpose of this study is to learn more about the safety, effectiveness and tolerability of the study drug called Benfotiamine, to determine whether it delays or slows the progression of the symptoms of early Alzheimer's disease.

This is a research study to test an investigational drug. An investigational drug is one that is not approved by *the United States Food and Drug Administration (FDA)* for use in the disease under study. The use of Benfotiamine in this study is investigational. Some participants in this study will take placebo, which are capsules that look like the study drug but do not contain any active drug in them. Neither you nor the study personnel will know whether you are receiving the active study drug or placebo.

NUMBER OF PARTICIPANTS / LENGTH OF PARTICIPATION
Approximately 406 participants will be invited to join the study for a duration of 18 months.

WHAT WILL HAPPEN DURING THE STUDY
Your participation in this study will last approximately 18 months (76 weeks) and will include approximately 11 study visits to the study site. You will be asked to bring your study partner to these visits. Your study partner must be someone who will have frequent interaction with you (at least 3-4 times per week), will be available for all clinic visits in person or remotely, and can help you comply with study procedures.

This study will use competitive enrollment. This means that when a target number of participants begins the study, all further enrollment will be closed. Therefore, it is possible that you could be in the screening phase, ready to begin the study, and be discontinued without your consent if the target number of participants has already begun the study.

Screening (Visit 1):
Before any study-related tests and procedures are performed, you will be asked to read and sign this consent document. The following screening tests and procedures will then be performed to determine if you qualify to take part in this study:

- **Demographic Questions:** You will be asked to give personal information about yourself, such as your name, date of birth and education.
- **Health and Medication Questions:** You will be asked to answer questions about your health, your medication history and your current medications.
- **Physical Exam:** You will have a physical exam. You should ask your study doctor about what will happen during the exam.
- **Neurological Exam:** You will have a neurological exam. You should ask your study doctor about what will happen during the exam.
- **Height and Weight:** We will measure how tall you are and how much you weigh.
- **Vital Signs:** Your blood pressure will be checked by putting a cuff around your arm. This will squeeze your arm for about a minute. We will check your pulse, listen to you breathe in and out (respiration rate), and take your temperature. This will be done while you are sitting down.
- **Electrocardiogram (ECG):** ECG stands for electrocardiogram which is a test that measures the electrical signals of your heart.

- **Blood testing:** We will collect between about 3 to 4 tablespoons of blood from your arm.
    - You will have laboratory tests including complete blood count, blood chemistries, blood sugar level, thyroid hormone levels, vitamin B12, folate (a type of vitamin B that helps produce red blood cells), and infectious disease tests including HIV, Hepatitis A, B, C, and syphilis. This is to make sure you have no medical conditions that prevent your participation in this study. Your study doctor may be required by law to report the result of infectious disease tests to the local health authority.
    - To determine your eligibility to participate in this study, you will have a blood test that estimates the likelihood that you have markers of Alzheimer's disease.
        - Only persons with a test result showing a high likelihood of having Alzheimer's disease markers, and who meet all other inclusion criteria, will be eligible to receive study treatment. You will not receive specific detailed results of this blood testing which is being done for research purposes only.
    - We will also store a portion of your blood for future research testing.
- **Urine Testing:** A urine sample will be collected to do laboratory tests.
- **Brain MRI:** MRI stands for magnetic resonance imaging. A brain MRI takes pictures of your brain and measures your brain volume and structure. If you have not had an MRI in the past 6 months, you will need to get one. If you have had an MRI in the past 6 months, you may not need to get one for this study.
- **Memory Testing:** You will be asked questions and will do activities to test your memory and thinking. Your study doctor can tell you more about the tests. Some questions may make you feel uncomfortable, and you are free not to answer those questions. You will also be asked about your daily activities, your mood, and your behavior. These are different rating scales that will be done by your study doctor or study staff.
- **Suicidality questionnaire:** We will ask you if you are feeling irritable or angry, restless, depressed or sad, if you have had problems sleeping, or any strange or unusual experiences, including suicidal thoughts. If you disclose suicidal thoughts, an appropriate referral and care will be discussed with you.
    - If you are having suicidal thoughts or feel in crisis, call the study doctor at the telephone number listed on the first page of this form. You can also call or text the National Suicide & Crisis Lifeline at 9-8-8 or 1-800-273-TALK (8255). The Lifeline numbers are answered 24 hours a day every day of the year by a skilled, trained counselor.
- We will ask you about your history of alcohol and substance abuse within the past five years and any current practices.

If you qualify to take part in this study and go on to receive the study treatment, then the following will happen:

Study Treatment:
You will be randomly assigned by chance (like the flip of a coin) to receive either study drug or placebo (inactive substance). Overall, half the study participants will receive study drug, and half will receive placebo. This is a double-blind study, which means neither you nor the study doctor will know to which of these study drug groups you are assigned. In case of an emergency, however, the study doctor can get this information.

Baseline (Visit 2)

- Health and Medication Questions: You will be asked about any illnesses or changes in your health or medication you are taking since your last visit.
- Physical exam: You should ask your study doctor about what will happen during the exam.
- Vital signs (sitting blood pressure, pulse, temperature, respiration rate)
- Weight
- Blood testing: We will collect approximately 3 to 4 tablespoons of blood from your arm.
    - Laboratory tests including complete blood count and blood chemistries, and research testing.
        - You will have additional blood drawn just prior to your morning dose of study drug. This kind of blood test is called pharmacokinetics (PK test), and the purpose is to show how your body absorbs, distributes, and gets rid of the study drug. Your blood will be drawn again 2 hours (+/- 30 minutes) after the morning dose at this baseline visit to test the PK values again.
    - We will also store a portion of your blood for future research testing.
- Lumbar puncture (Optional): A lumbar puncture is a procedure that involves inserting a needle in the lower back in order to collect a small amount of the spinal fluid that surrounds the brain and spinal cord. During the procedure you will lay on your side curled up into a ball or you will sit on the edge of a chair or bed and lean forward, whichever is easier. The lower part of your back will be cleaned with antiseptic. A local anesthetic (lidocaine, 1%) will be injected into the skin of your lower back at the area of the lumbar puncture. When the area is numb, a very thin needle will be inserted into the spinal canal in the lower back, well below the level where the spinal cord ends. Approximately 2 tablespoons of spinal fluid (that surrounds the brain and spinal cord) will be collected for analysis of markers of Alzheimer’s disease. After the lumbar puncture is complete, you will remain at the study site for about 30 minutes. You will be given something to eat and drink before you leave. You should avoid any strenuous physical activity for the next 24 hours. This includes lifting, bending, doing housework and gardening, or doing exercise such as jogging or bicycle riding. Study staff will call you 1-3 days following your lumbar puncture to discuss how you are feeling.

- If, for technical reasons, a standard lumbar puncture is not possible, you may have fluoroscopy or other image-guided procedure performed to complete the lumbar puncture.
- Fluoroscopy is a continuous X-ray that shows images on a computer monitor. This is used during a lumbar puncture to help guide the needle insertion between two lumbar vertebrae and then into the spinal canal.
- If image-guided techniques other than fluoroscopy is used by your site to conduct a lumbar puncture, the study doctor or study staff will provide you with the description and risks of the techniques.

- Suicidality questionnaire: We will ask you if you are feeling irritable or angry, restless, depressed or sad, if you have had problems sleeping, or any strange or unusual experiences, including suicidal thoughts. If you disclose suicidal thoughts, an appropriate referral and care will be discussed with you.
- Memory Testing: At this visit, a portion of these memory tests will be administered in clinic and a portion of these memory tests will be administered remotely at home over video call with a member of the study staff. You will use your own devices for the remote assessments if they have capabilities for a video call. If you do not have a device with capabilities for a video call, a device may be provided to you by the study staff. You will be instructed to use the same device for all remote assessments.
- We will ask you about your experience in your participation in this research study.

At the completion of the baseline visit procedures, you will be assigned randomly (like the flip of the coin) to one of two groups, either to study drug capsules or to matching placebo capsules that do not contain any active drug. You will take your first dose of study drug in the clinic at the baseline visit. You will then receive instructions on how to take the capsules at home and asked to return to the study site in 4 weeks.

Week 4 (Visit 3)

- Health and Medication Questions: You will be asked about any adverse events (negative changes in health) you may have experienced or any changes in medication you are taking since last visit.
- Physical exam: Ask your study doctor about what will happen during the exam.
- Vital signs
- Weight
- Suicidality questionnaire: We will ask you if you are feeling irritable or angry, restless, depressed or sad, if you have had problems sleeping, or any strange or unusual experiences, including suicidal thoughts. If you disclose suicidal thoughts, an appropriate referral and care will be discussed with you.
- Blood testing: We will collect approximately 2 to 3 tablespoons of blood from your arm for complete blood count and blood chemistries.

You will then receive more study drug, instructions on how to take the capsules, and asked to return to the study site in 4 weeks.

Week 8 (Visit 4)

- Health and Medication Questions: You will be asked about any adverse events (negative changes in health) you may have experienced or any changes in medication you are taking since last visit.
- Physical exam: Ask your study doctor about what will happen during the exam.
- Vital signs
- Weight
- Suicidality questionnaire: We will ask you if you are feeling irritable or angry, restless, depressed or sad, if you have had problems sleeping, or any strange or unusual experiences, including suicidal thoughts. If you disclose suicidal thoughts, an appropriate referral and care will be discussed with you.
- Blood testing: We will collect approximately 3 to 4 tablespoons of blood from your arm.
    - Laboratory tests including complete blood count and blood chemistries.
    - You will have additional blood drawn just prior to your morning dose of study drug, and again 2 hours after taking your morning dose of study drug. This kind of blood test is called pharmacokinetics, and the purpose is to show how your body absorbs, distributes, and gets rid of the study drug.

You will then receive more study drug, instructions on how to take the capsules, and asked to return to the study site in 4 weeks.

Week 12 (Visit 5)

- Health and Medication Questions: You will be asked about any adverse events (negative changes in health) you may have experienced or any changes in medication you are taking since last visit.
- Physical exam: Ask your study doctor about what will happen during the exam.
- Vital signs
- Weight
- Suicidality questionnaire: We will ask you if you are feeling irritable or angry, restless, depressed or sad, if you have had problems sleeping, or any strange or unusual experiences, including suicidal thoughts. If you disclose suicidal thoughts, an appropriate referral and care will be discussed with you.
- Blood testing: We will collect approximately 2 to 3 tablespoons of blood from your arm for complete blood count and blood chemistries.

You will then receive more study drug, instructions on how to take the capsules, and asked to return to the study site in 12 weeks.

Week 24 (Visit 6)

- Health and Medication Questions: You will be asked about any adverse events (negative changes in health) you may have experienced or any changes in medication you are taking since last visit.
- Physical exam: Ask your study doctor about what will happen during the exam.
- Vital signs
- Weight
- ECG: ECG stands for electrocardiogram which is a test that measures the electrical signals of your heart.
- Memory Testing: At this visit, a portion of these memory tests will be administered in clinic and a portion of these memory tests will be administered remotely at home over video call with a member of the study staff. You will use your own devices for the remote assessments if they have capabilities for a video call. If you do not have a device with capabilities for a video call, a device may be provided to you by the study staff. You will be instructed to use the same device for all remote assessments.
- Suicidality questionnaire: We will ask you if you are feeling irritable or angry, restless, depressed or sad, if you have had problems sleeping, or any strange or unusual experiences, including suicidal thoughts. If you disclose suicidal thoughts, an appropriate referral and care will be discussed with you.
- Blood testing: We will collect approximately 2 to 3 tablespoons of blood from your arm for complete blood count and blood chemistries.
- We will ask you about your experience in your participation in this research study.

You will then receive more study drug, instructions on how to take the capsules, and asked to return to the study site in 12 weeks.

Week 36 (Visit 7)

- Health and Medication Questions: You will be asked about any adverse events (negative changes in health) you may have experienced or any changes in medication you are taking since last visit.
- Physical exam: Ask your study doctor about what will happen during the exam.
- Vital signs
- Weight
- Suicidality questionnaire: We will ask you if you are feeling irritable or angry, restless, depressed or sad, if you have had problems sleeping, or any strange or unusual experiences, including suicidal thoughts. If you disclose suicidal thoughts, an appropriate referral and care will be discussed with you.
- Blood testing: We will collect approximately 2 to 3 tablespoons of blood from your arm for complete blood count and blood chemistries.

You will then receive more study drug, instructions on how to take the capsules, and asked to return to the study site in 12 weeks.

Week 48 (Visit 8)

- Health and Medication Questions: You will be asked about any adverse events (negative changes in health) you may have experienced or any changes in medication you are taking since last visit.
- Physical exam: Ask your study doctor about what will happen during the exam.
- Vital signs
- Weight
- Memory Testing: At this visit, a portion of these memory tests will be administered in clinic and a portion of these memory tests will be administered remotely at home over video call with a member of the study staff. You will use your own devices for the remote assessments if they have capabilities for a video call. If you do not have a device with capabilities for a video call, a device may be provided to you by the study staff. You will be instructed to use the same device for all remote assessments.
- Suicidality questionnaire: We will ask you if you are feeling irritable or angry, restless, depressed or sad, if you have had problems sleeping, or any strange or unusual experiences, including suicidal thoughts. If you disclose suicidal thoughts, an appropriate referral and care will be discussed with you.
- Blood testing: We will collect approximately 2-3 tablespoons of blood from your arm.
    - Laboratory tests including complete blood count, blood chemistries, and research testing.
- We will ask you about your experience in your participation in this research study.

You will then receive more study drug, instructions on how to take the capsules, and asked to return to the study site in 12 weeks.

Week 60 (Visit 9)

- Health and Medication Questions: You will be asked about any adverse events (negative changes in health) you may have experienced or any changes in medication you are taking since last visit.
- Physical exam: Ask your study doctor about what will happen during the exam.
- Vital signs
- Weight
- Suicidality questionnaire: We will ask you if you are feeling irritable or angry, restless, depressed or sad, if you have had problems sleeping, or any strange or unusual experiences, including suicidal thoughts. If you disclose suicidal thoughts, an appropriate referral and care will be discussed with you.
- Blood testing: We will collect approximately 2 to 3 tablespoons of blood from your arm for complete blood count and blood chemistries.

You will then receive your last supply of study drug, instructions on how to take the capsules, and asked to return to the study site in 12 weeks.

Week 72 (Visit 10) or Early Termination

- Health and Medication Questions: You will be asked about any adverse events (negative changes in health) you may have experienced or any changes in medication you are taking since last visit.
- Physical exam: Ask your study doctor about what will happen during the exam.
- Vital signs
- Weight
- ECG: ECG stands for electrocardiogram which is a test that measures the electrical signals of your heart.
- Memory Testing: At this visit, a portion of these memory tests will be administered in clinic and a portion of these memory tests will be administered remotely at home over video call with a member of the study staff. You will use your own devices for the remote assessments if they have capabilities for a video call. If you do not have a device with capabilities for a video call, a device may be provided to you by the study staff. You will be instructed to use the same device for all remote assessments.
- Blood testing: We will collect approximately 3 to 4 tablespoons of blood from your arm.
    - Laboratory tests including complete blood count, blood chemistries, and research testing, including:
        - A blood test called pharmacokinetics (PK), to show how your body absorbs, distributes, and gets rid of the study drug. We will also store a portion of your blood samples for future research.
- Suicidality questionnaire: We will ask you if you are feeling irritable or angry, restless, depressed or sad, if you have had problems sleeping, or any strange or unusual experiences, including suicidal thoughts. If you disclose suicidal thoughts, an appropriate referral and care will be discussed with you.
- A brain MRI to take pictures of your brain and to measure your brain volume and structure.
- Lumbar puncture (Optional): approximately 2 tablespoons of spinal fluid (that surrounds the brain and spinal cord) will be collected for analysis of markers of Alzheimer's disease. There will be a telephone call to see how you are, 1 to 3 days after the procedure.
- We will ask you about your experience in your participation in this research study.

You will receive a follow-up telephone call from a member of the study staff 4 weeks after your Week 72 visit to see how you are. As needed, your study doctor may request that this visit be done in clinic.

Week 76 Follow-up (Visit 11)

- Health and Medication Questions: You will be asked about any adverse events (negative changes in health) you may have experienced or any changes in medication you are taking since last visit.

If you discontinue study treatment for any reason during the study period, you will be asked to continue participation in the study and attend the remaining study site visits with your ongoing consent and approval from your study doctor.

After Study Treatment:
Because this is a research study, the study drug will be given to you only during this study and not after the study is over.

EXPECTATIONS:
If you participate in this study, you and your study partner will be expected to attend each study visit, take the study drug as instructed and follow instructions from your study doctor.

RISKS, SIDE EFFECTS, AND/OR DISCOMFORTS
The safety of benfotiamine in humans has already been tested in multiple clinical trials, including in participants with diabetic peripheral neuropathy where it is shown to have some benefit. One study of 40 participants with diabetic peripheral neuropathy treated with benfotiamine 400 mg per day for three weeks reported no side effects. In a second study, benfotiamine was administered to 165 participants in ten different study sites in Germany for six weeks. In both the 300 mg per day and 600 mg per day benfotiamine groups, the number of participants with adverse events was comparable to placebo (inactive substance) demonstrating the good tolerability of a daily dose of 300 mg or 600 mg of benfotiamine.

There have been three clinical trials testing the efficacy and safety of benfotiamine in participants with Alzheimer's disease. One of these trials tested benfotiamine at 600 mg per day for 12 months in persons with early Alzheimer's disease who are similar to those who are participating in the current study. The number of participants with serious side effects were similar between those who received benfotiamine in comparison to those who received placebo, which is an inactive pill. The total number of side effects in those who received placebo were 59 (in 36 participants) whereas the total number of side effects in those treated with benfotiamine were 38 (in 34 participants). These previous studies suggest that benfotiamine has a favorable safety profile when used in doses of up to 600 mg for up to 12 months. The table describes the side effects reported by the 36 participants who received placebo and the 34 participants who received Benfotiamine. None of the participants in this study withdrew due to these side effects and were able to complete the study.

**Symptom/Side effect/Adverse Event**	**Placebo (out of 36 participants)**	**Benfotiamine Treatment (out of 34 participants) 600 mg daily**
Anxiety	4 (11%)	5 (14%)
Bruise	5 (14%)	2 (16%)
Cold symptoms	3 (8%)	3 (18%)
Depression	2 (6%)	1 (3%)

Dizziness	3 (8%)	3 (8%)
Fall	12 (34%)	6 (17%)
Head Injury	2 (6%)	0 (0%)
Heart arrhythmia (irregular heartbeat)	2 (6%)	1 (3%)
Pain	4 (11%)	5 (14%)
Pneumonia	3 (8%)	0 (0%)
Sprain	2 (6%)	0 (0%)
Surgery	3 (8%)	1 (3%)
Allergy	2 (2%)	0 (0%)
Gastrointestinal (stomach or intestines) problem	12 (34%)	9 (26%)
Stroke	0 (0%)	2 (6%)

In this study in which you are being asked to participate, both the same dosage (600 mg per day) as in the previous AD study, and a higher dose of benfotiamine (1200 mg per day) are being tested for a longer time (18 months). Safety will be carefully monitored in this study because we are testing a higher dose for a longer duration. This study will look at whether benfotiamine is as safe as in previous studies compared with placebo.

If you receive placebo (the inactive substance) as part of this study, your symptoms of Alzheimer's disease may or may not improve. You will be followed closely during the study whether you are assigned to placebo, an inactive substance, or benfotiamine to see if your symptoms of Alzheimer's disease improve, remain unchanged or get worse.

RISKS OF STUDY PROCEDURES

- Blood samples: Possible side effects from blood drawing include faintness, inflammation of the vein, pain, bruising, or bleeding at the site of puncture. There is also a slight possibility of infection.
- Electrocardiogram (ECG): Skin irritation is rare but could occur during an ECG from the electrodes or gel that are used.
- Questionnaires: The questionnaires used in this study may be upsetting. You do not need to answer any questions that you are not comfortable with.
- Lumbar Puncture:
    - During the lumbar puncture procedure, you may have temporary pain and discomfort in your back.
    - Headache may occur in about 5% of people who undergo a lumbar puncture. Less commonly, in about 1-4% of participants, a persistent low-pressure headache may develop, probably due to leakage of CSF.
    - Uncommonly, a blood patch (injection of some of your blood into the lumbar puncture site to patch the CSF leak) may be required if you experience a persistent headache after the lumbar puncture. This should rapidly relieve the headache.
    - Although very rare, it is possible that you may have an allergic reaction to the local anesthetic (lidocaine 1%) used for the lumbar puncture. An allergic reaction

would cause swelling and a rash on your skin where the anesthetic was injected. Please tell us if you have ever had a reaction to local anesthetic before (such as when you were visiting the dentist).

  - Potential but rare risks of lumbar puncture include infection, damage to nerves in your back, paralysis and bleeding into the CSF space. The risk of these is much less than 1% (1 person in 100).

- Radiation Risk (if applicable)
  The maximum amount of radiation from the research-related radiation procedures (Fluoroscopy) in this study is equivalent to approximately 18% (that is, 0.18 times) of the annual radiation limit for a medical worker. Long term effects on your health such as cancer cannot be ruled out from this amount of radiation. This dose estimate takes into account only the exposure to procedures in this project. If you have participated in other research studies involving radiation exposure, you should be aware that the risk of effects from radiation is believed to increase with each exposure you receive (including studies performed as part of your medical care).
- Magnetic Resonance Imaging (MRI): There are no known biological risks associated with MR imaging but it may cause possible anxiety for people due to loud banging made by the machine and the confined space of the testing area. There is also a risk of injury if metal is brought into the imaging room, which might be pulled into the MRI magnet. People with pacemakers, aneurysm clips, artificial heart valves, ear implants or metal/foreign objects in their eyes are not permitted to have an MRI.

UNFORESEEN RISKS

Since this is an investigational study, there may be other risks that are unknown. All drugs have a potential risk of an allergic reaction, which if not treated promptly, could become life-threatening. Some symptoms of allergic reactions are:

- Rash
- Wheezing and difficulty breathing
- Dizziness and fainting
- Swelling around the mouth, throat or eyes
- A fast pulse
- Sweating

Please seek treatment immediately and tell the study doctor and study staff if you have any of these symptoms.

BIRTH CONTROL RESTRICTIONS

Taking the study drug may involve risks to a pregnant woman, an embryo, fetus (unborn baby) or nursing infant. Therefore, if you are pregnant, planning to become pregnant, planning to father a child, or are breastfeeding a child, you cannot participate in this study.

Females:
In order to reduce the risk of pregnancy, you must be post-menopausal for at least one year or surgically sterile (bilateral tubal ligation, hysterectomy, or bilateral oophorectomy) for at least 6 months prior to screening.

Males:
In order to reduce the risk of pregnancy, you should use an effective method of birth control (for example, condoms, abstinence, etc.) during study treatment and for the duration of the study.

ALTERNATIVES TO PARTICIPATION
You do not have to be in this study to receive treatment for your early Alzheimer's disease.

Your options may include:

- Donepezil (Aricept®), memantine (Namenda®), rivastigmine (Exelon®), galantamine (Razadyne®) and Namzeric®. Taking these drugs may or may not improve your condition. Your study doctor will discuss with you the risks and benefits of the alternative treatments. Please talk to the study doctor about your options before you decide whether or not you will take part in this study. As an alternative to being in this study, you may ask your doctor about receiving amyloid lowering treatments. Leqembi® (an amyloid lowering antibody also known as lecanemab) has received full approval by the FDA for the treatment of early Alzheimer's disease. Aduhelm® (another amyloid lowering antibody also known as aducanumab) has received accelerated approval by the FDA for the treatment of early Alzheimer's disease. Please talk to the study doctor about your options before you decide whether or not you will take part in this study.

This research study is for research purposes only. The only alternative is to not participate in this study.

NEW FINDINGS
Any new important information that is discovered during the study and which may influence your willingness to continue participation in the study will be provided to you.

BENEFITS
You may benefit as a result of your participation in this study. There is, however, no guarantee that you will benefit from your participation in this study. Information learned from the study may help other people in the future.

COMPENSATION FOR PARTICIPATION
«Compensation»

You will be paid up to a total of $1,100.00 if you complete this study. Your study partner will be paid up to a total of $1,100.00 if you complete this study. You will be paid for the visits you complete according to the following schedule:

- $100.00 participant stipend for visits: Screening, Baseline, Week 4, Week 8, Week 12, Week 24, Week 36, Week 48, Week 60, Week 72, and Week 76.
- $100.00 study partner stipend for visits: Screening, Baseline, Week 4, Week 8, Week 12, Week 24, Week 36, Week 48, Week 60, Week 72, and Week 76.

If you do not complete the study, for any reason, you will be paid for each study visit you do complete.

You will be paid ___________ ***["following each completed visit", "monthly", "quarterly", "at the end of your participation in the research study", "following each completed visit or at the end of your participation in the research study, whichever you prefer"]***.

If you have any questions regarding your compensation for participation, please contact the study staff.

[If applicable:] We will reimburse you for the cost of ***[describe: e.g., traveling to your study visits]***. You will be reimbursed approximately ***[e.g., 2 weeks, one month, etc.]*** after you submit your travel receipts to the study staff.

Site: Please add your Site/Institute required language in reference to taxable income to the Internal Review Service (IRS) if applicable.

In addition to receiving compensation for participating in this study, all enrolled and eligible participants will be offered a handmade quilt by the Alzheimer's Disease Cooperative Study (ADCS). These quilts have been donated to ADCS by nonprofit organizations and have no monetary value.

CONFIDENTIALITY

Records of your participation in this study will be held confidential except when sharing the information is required by law or as described in this informed consent. The study doctor, the sponsor or persons working on behalf of the sponsor, and under certain circumstances, the United States Food and Drug Administration (FDA) and the Institutional Review Board (IRB) will be able to inspect and copy confidential study-related records which identify you by name. This means that absolute confidentiality cannot be guaranteed. If the results of this study are published or presented at meetings, you will not be identified.

A description of this clinical trial will be available on http://www.ClinicalTrials.gov, as required by U.S. Law. This Web site will not include information that can identify you. At most, the Web site will include a summary of the results. You can search this Web site at any time.

Certificate of Confidentiality

This research is covered by a Certificate of Confidentiality from the National Institutes of Health. The researchers with this Certificate may not disclose or use information, documents, or biospecimens that may identify you in any federal, state, or local civil, criminal, administrative, legislative, or other action, suit, or proceeding, or be used as evidence, for example, if there is a court subpoena, unless you have consented for this use. Information, documents, or biospecimens protected by this Certificate cannot be disclosed to anyone else who is not connected with the research except, if there is a federal, state, or local law that requires disclosure (see below); if you have consented to the disclosure, including for your medical treatment; or if it is used for other scientific research, as allowed by federal regulations protecting research participants.

The Certificate cannot be used to refuse a request for information from personnel of the United States federal or state government agency sponsoring the project that is needed for auditing or program evaluation by the agency which is funding this project or for information that must be disclosed in order to meet the requirements of the federal Food and Drug Administration (FDA). You should understand that a Certificate of Confidentiality does not prevent you from voluntarily releasing information about yourself or your involvement in this research. If you want your research information released to an insurer, medical care provider, or any other person not connected with the research, you must provide consent to allow the researchers to release it.

A federal law, called the Genetic Information Nondiscrimination Act (GINA), generally makes it illegal for health insurance companies, group health plans and most employers to discriminate against you based on your genetic information.

This law generally will protect you in the following ways:

- Health insurance companies and group health plans may not request your genetic information that the sponsor will get from this research.
- Health insurance companies and group health plans may not use your genetic information when making decisions regarding your eligibility or premiums.
- Employers with 15 (determined by State's law) or more employees may not use your genetic information that the sponsor will get from this research when making a decision to hire, promote, or fire you or when setting the terms of your employment.

All health insurance companies and group health plans and all employers with 15 (or determined by State's law) or more people must follow this law.

Be aware that this Federal law does not protect you against genetic discrimination by companies that sell life insurance, disability insurance or long-term care insurance.

COMPENSATION FOR INJURY

If you become ill or are injured while you are in the study, get the medical care that you need right away. You should inform the healthcare professional treating you that you are participating in this study. If you tell the study staff that you think you have been injured then they will help you get the care you need.

All forms of medical findings and treatments – whether routine or investigational – involve some risk of injury. In spite of all safety measures, you might develop medical problems from participating in this study.

You must report any suspected illness or injury to the study doctor immediately. If such problems take place, the study site will provide emergency medical treatment and will assist you in getting proper follow-up medical treatment. There are no plans to provide financial compensation nor reimbursement for such things as pre-existing conditions, illness or disease unrelated to study participation, lost wages, property damage, disability, or discomfort is available.

The National Institutes of Health, Winifred Masterson Burke Medical Research Institute, and the Alzheimer's Disease Cooperative Study do not provide compensation for research-related injury.

COSTS

There will be no charge to you for your participation in this study. The study drug, study-related procedures, and study visits will be provided at no charge to you or your insurance company.

FUTURE RESEARCH STUDIES

Identifiers will be removed from your identifiable private information or identifiable biospecimens (samples taken, such as blood and DNA) collected during this study and could then be used for future research studies or distributed to other qualified researchers for future research studies without additional informed consent.

COMMERCIAL PROFIT

Your biospecimens collected during this study may be used for commercial profit (even if identifiers are removed) and you will not share in this profit.

CLINICALLY RELEVANT RESULTS

Those research results which are abnormal and clinically relevant during the study, and which could impact your medical care will be disclosed to you. You will also have the chance to have these results shared with your primary care physician. You will be asked to agree or disagree to sharing these results with your primary care physician.

GENOME SEQUENCING

You will be asked to contribute a sample for DNA testing as part of this research study. You will not receive any results of this DNA testing, but the results will be used in the analysis of the study information. With the sample of DNA that you provide, researchers will be able to look closely at large amounts of your genetic information by sequencing, or "reading", every letter in your DNA (your genome). Reading a person's entire genetic code is called whole genome sequencing. Potential future research on your sample may include whole genome sequencing. You will not receive the results of these investigations.

WHOM TO CONTACT ABOUT THIS STUDY

During the study, if you experience any medical problems, suffer a research-related injury, or have questions, concerns or complaints about the study such as:

- Whom to contact in the case of a research-related injury or illness;
- Payment or compensation for being in the study, if any;
- Your responsibilities as a research participant;
- Eligibility to participate in the study;
- The study doctor or study site's decision to withdraw you from participation;
- Results of tests and/or procedures;

Please contact the study doctor at the telephone number listed on the first page of this consent document.

If you seek emergency care, or hospitalization is required, alert the treating physician that you are participating in this research study.

An institutional review board (IRB) is an independent committee established to help protect the rights of research participants. If you have any questions about your rights as a research participant, contact:

- By **mail**:
  Study Participant Adviser
  Advarra IRB
  6100 Merriweather Dr., Suite 600
  Columbia, MD 21044
- or call **toll free**: 877-992-4724
- or by **email**: adviser@advarra.com

Please reference the following number when contacting the Study Participant Adviser: Pro00071249.

VOLUNTARY PARTICIPATION / WITHDRAWAL

Your decision to participate in this study is voluntary. You may choose to not participate or you may withdraw from the study for any reason without penalty or loss of benefits to which you are otherwise entitled and without any effect on your future medical care. However, please note that the FDA requires that any information collected up to the point of your withdrawal cannot be removed from the study.

The study doctor or the sponsor can stop your participation at any time without your consent for the following reasons:

- If it appears to be medically harmful to you;
- If you fail to follow directions for participating in the study;
- If it is discovered that you do not meet the study requirements;
- If the study is canceled; or
- For administrative reasons.

If you leave the study for any reason, the study doctor may ask you to have some end-of-study tests for your safety.

If you do withdraw from the study, then you may request that your data and any unused sample be destroyed. However, data and samples that have already been distributed to approved researchers will not be retrieved.

LUMBAR PUNCTURE (OPTIONAL)

Please indicate your preference below:

_____ (initials) I **agree** to participate in the optional Lumbar Puncture described at Baseline and week 72 (above).

_____ (initials) I **do not agree** to participate in the optional Lumbar Puncture described at Baseline and week 72 (above).

PRIMARY HEALTH CARE PROVIDER NOTIFICATION OPTION

I consent to having my primary health care provider notified by the study site of my participation in this study and/or any abnormal and clinically relevant findings related to my health (please check yes or no).
❒**YES** (If yes, please complete the information below)
❒**NO**

Name and address of family doctor or primary health care provider:	Name:
	Address:
Telephone and Fax Number:	Tel:
	Fax:

CONSENT

I have read and understand the information in this informed consent document. I have had an opportunity to ask questions and all of my questions have been answered to my satisfaction. I voluntarily agree to participate in this study until I decide otherwise. I do not give up any of my legal rights by signing this consent document. I will receive a copy of this signed consent document.

____

Participant's Printed Name

____ ___________

Participant's Signature Date

____

Printed Name of the Person Conducting the Consent Discussion

____ ___________

Signature of the Person Conducting the Consent Discussion Date

OR

WHEN/IF APPLICABLE:

____

Printed Name of Legally Authorized Representative

____ ___________

Signature of Legally Authorized Representative Date

____

Authority of Legally Authorized Representative to act on behalf of Participant

WITNESS SIGNATURE FOR PARTICIPANTS WHO CANNOT READ

The study participant has indicated that he/she is unable to read. The consent document has been read to the participant by a member of the study staff, discussed with the participant by a member of the study staff, and the participant has been given an opportunity to ask questions of the study staff.

____
Printed Name of Impartial Witness

____ ____________
Signature of Impartial Witness Date
