# Supplementary material for: Protocol for a seamless phase 2A-phase 2B randomized double-blind placebo-controlled trial to evaluate the safety and efficacy of benfotiamine in patients with early Alzheimer’s disease (BenfoTeam)
Source: PLoS One. 2024 May 29;19(5):e0302998. doi: 10.1371/journal.pone.0302998 (PMC11135745; doi:10.1371/journal.pone.0302998)
Supplement: Peer review history

# Editor report: Decision Letter 0

**DOI:** 10.1371/journal.pone.0302998.r001

**Authors:** Emma Campbell

8 May 2024

Protocol for a seamless phase 2A-phase 2B randomized double-blind placebo-controlled trial to evaluate the safety and efficacy of benfotiamine in patients with early Alzheimer's disease (BenfoTeam)

PONE-D-24-14392

Dear Dr. Feldman,

We’re pleased to inform you that your manuscript has been judged scientifically suitable for publication and will be formally accepted for publication once it meets all outstanding technical requirements.

As a Peer-Reviewed Funded Protocol, your submission is eligible for expedited review by in-house editors. Based on our evaluation, we are satisfied that your manuscript meets our publication criteria for Study Protocols, and is therefore considered to be suitable for publication subject to final journal requirements.

Within one week, you’ll receive an e-mail detailing the required amendments. When these have been addressed, you’ll receive a formal acceptance letter and your manuscript will be scheduled for publication.

An invoice will be generated when your article is formally accepted. Please note, if your institution has a publishing partnership with PLOS and your article meets the relevant criteria, all or part of your publication costs will be covered. Please make sure your user information is up-to-date by logging into Editorial Manager at Editorial Manager® and clicking the ‘Update My Information' link at the top of the page. If you have any questions relating to publication charges, please contact our Author Billing department directly at authorbilling@plos.org.

If your institution or institutions have a press office, please notify them about your upcoming paper to help maximize its impact. If they’ll be preparing press materials, please inform our press team as soon as possible -- no later than 48 hours after receiving the formal acceptance. Your manuscript will remain under strict press embargo until 2 pm Eastern Time on the date of publication. For more information, please contact onepress@plos.org.

Journal requirements:

When submitting your revision, we need you to address these additional requirements.

1. Please ensure that your manuscript meets PLOS ONE's style requirements, including those for file naming. The PLOS ONE style templates can be found at

https://journals.plos.org/plosone/s/file?id=wjVg/PLOSOne_formatting_sample_main_body.pdf and

https://journals.plos.org/plosone/s/file?id=ba62/PLOSOne_formatting_sample_title_authors_affiliations.pdf

2. Thank you for stating the following in the Competing Interests section:

“I have read the journal's policy and the authors of this manuscript have the following competing interests: SDE is a Data Safety Monitoring Board statistician for clinical trials performed by Janssen Research & Development LLC and Suven. For this manuscript, HHF reports grant funding from the National Institute of Aging (R01AG076634) with no personal funds received and all payments to UC San Diego. HHF also discloses grant funding from Annovis (QR Pharma), Vivoryon (Probiodrug), Biohaven Pharmaceuticals, AC Immune, and LuMind Foundation; service agreements for consulting activities with LuMind, Genentech (DSMB), Roche/Banner (DMC), Tau Consortium (SAB), Axon Neuroscience, Biosplice Therapeutics, Janssen Research & Development LLC (DMC), Arrowhead Pharmaceuticals, Samus Therapeutics; Advisory Board activities: Translating Research in Elder Care (TREC), Association for Frontotemporal Dementia, Tau Consortium and Rainwater Charitable Foundation; support for travel from Novo Nordisk, Royal Society of Canada, Tau Consortium, Canadian Consortium for Neurodegeneration in Aging (CCNA), Translating Research in Elder Care (TREC), World Events Forum (ADDF); and a philanthropic donation for the Epstein Family Alzheimer Research Collaboration. For these activities, no personal funds have been received with all payments to UC San Diego. HHF personally receives royalties for patent: Feldman HH (filed November 26, 2008). Detecting and Treating Dementia Serial Number 12/3-2691 U.S. Patent No. PCT/US2007/07008. Washington, DC: U.S. Patent and Trademark Office. DMJ reports support from grants to UC San Diego from NIH/NIA, Vivoryon Therapeutics, Biohaven Pharmaceuticals, and the Epstein Family Foundation. SAF reports grant funding from the NIH. GEG reports a pending patent. KJ received funding from BBSRC and the National Institute for Health and Care Research (NIHR) Cambridge Biomedical Research Centre. AK receives funding from the UKRI (MRC & BBSRC), the Wellcome Trust, and the National Institute for Health and Care Research (NIHR) Cambridge Biomedical Research Centre and is a paid consultant for the company Juvenescence. JAL was a consultant for Merck KGaA in 2022 (no longer active); receives drug/placebo from EMD Serono, Merck Subsidiary, for another clinical trial; and receives a stipend from Wolters Kluwer as Editor In Chief of the journal Alzheimer's Disease and Associated Disorders. KEY, PBV, and VV are employed by and have equity interests in C2N Diagnostics. C2N Diagnostics has received support from the NIH (grant No. R44 AG059489), BrightFocus Foundation (grant No. CA2016636), The Gerald and Henrietta Rauenhorst Foundation, and the Alzheimer’s Drug Discovery Foundation (grant No. GC-201711-2013978). HZ has served at scientific advisory boards and/or as a consultant for Abbvie, Acumen, Alector, Alzinova, ALZPath, Amylyx, Annexon, Apellis, Artery Therapeutics, AZTherapies, Cognito Therapeutics, CogRx, Denali, Eisai, Merry Life, Nervgen, Novo Nordisk, Optoceutics, Passage Bio, Pinteon Therapeutics, Prothena, Red Abbey Labs, reMYND, Roche, Samumed, Siemens Healthineers, Triplet Therapeutics, and Wave, has given lectures in symposia sponsored by Alzecure, Biogen, Cellectricon, Fujirebio, Lilly, Novo Nordisk, and Roche, and is a co-founder of Brain Biomarker Solutions in Gothenburg AB (BBS), which is a part of the GU Ventures Incubator Program (outside submitted work).”

3. We note that the original protocol file you uploaded contains a confidentiality notice indicating that the protocol may not be shared publicly or be published. Please note, however, that the PLOS Editorial Policy requires that the original protocol be published alongside your manuscript in the event of acceptance. Please note that should your paper be accepted, all content including the protocol will be published under the Creative Commons Attribution (CC BY) 4.0 license, which means that it will be freely available online, and any third party is permitted to access, download, copy, distribute, and use these materials in any way, even commercially, with proper attribution.

Therefore, we ask that you please seek permission from the study sponsor or body imposing the restriction on sharing this document to publish this protocol under CC BY 4.0 if your work is accepted. We kindly ask that you upload a formal statement signed by an institutional representative clarifying whether you will be able to comply with this policy. Additionally, please upload a clean copy of the protocol with the confidentiality notice (and any copyrighted institutional logos or signatures) removed.

Kind regards,

Emma Campbell, Ph.D

Staff Editor

PLOS ONE

# Editor report: Acceptance letter

**DOI:** 10.1371/journal.pone.0302998.r002

**Authors:** Emma Campbell

16 May 2024

PONE-D-24-14392 

PLOS ONE

Dear Dr. Feldman, 

I'm pleased to inform you that your manuscript has been deemed suitable for publication in PLOS ONE. Congratulations! Your manuscript is now being handed over to our production team.

At this stage, our production department will prepare your paper for publication. This includes ensuring the following:

* All references, tables, and figures are properly cited

* All relevant supporting information is included in the manuscript submission,

* There are no issues that prevent the paper from being properly typeset

If revisions are needed, the production department will contact you directly to resolve them. If no revisions are needed, you will receive an email when the publication date has been set. At this time, we do not offer pre-publication proofs to authors during production of the accepted work. Please keep in mind that we are working through a large volume of accepted articles, so please give us a few weeks to review your paper and let you know the next and final steps. 

Lastly, if your institution or institutions have a press office, please let them know about your upcoming paper now to help maximize its impact. If they'll be preparing press materials, please inform our press team within the next 48 hours. Your manuscript will remain under strict press embargo until 2 pm Eastern Time on the date of publication. For more information, please contact onepress@plos.org.

If we can help with anything else, please email us at customercare@plos.org.

Thank you for submitting your work to PLOS ONE and supporting open access. 

Kind regards, 

PLOS ONE Editorial Office Staff

on behalf of

Dr Emma Campbell 

Staff Editor

PLOS ONE
